# A Non-inertial Model for Particle Aggregation Under Turbulence

**DOI:** 10.1007/s10955-025-03437-6

**Published:** 2025-03-25

**Authors:** Franco Flandoli, Ruojun Huang

**Affiliations:** 1 Scuola Normale Superiore di Pisa, Piazza Dei Cavalieri 7, 56126 Pisa, Italy; 2 Universität Münster, Einsteinstr. 62, 48149 Munster, Germany

**Keywords:** Particle coalescence, Turbulent fluid, Cell equation, Scaling limit, Saffman–Turner formula, 60K35, 82C27, 60H15, 86A08

## Abstract

We consider an abstract non-inertial model of aggregation under the influence of a Gaussian white noise with prescribed space-covariance, and prove a formula for the mean collision rate *R*, per unit of time and volume. Specializing the abstract theory to a non-inertial model obtained by an inertial one, with physical constants, in the limit of infinitesimal relaxation time of the particles, and the white noise obtained as an approximation of a Gaussian noise with correlation time $\tau _{\eta }$, up to approximations the formula reads $R\sim \tau _{\eta }\left\langle \left| \Delta _{a}u\right| ^{2}\right\rangle a\cdot n^{2}$ where *n* is the particle number per unit of volume and $\left\langle \left| \Delta _{a}u\right| ^{2}\right\rangle$ is the square-average of the increment of random velocity field *u* between points at distance *a*, the particle radius. If we choose the Kolmogorov time scale $\tau _{\eta }\sim \left( \frac{\nu }{\varepsilon }\right) ^{1/2}$ and we assume that *a* is in the dissipative range where $\left\langle \left| \Delta _{a}u\right| ^{2}\right\rangle \sim \left( \frac{\varepsilon }{\nu }\right) a^{2}$, we get Saffman–Turner formula for the collision rate *R*.

## Introduction

### The Abstract Mathematical Model and the Main Results

We consider non-inertial particles $x_{1}^{N}\left( t\right) ,...,x_{N} ^{N}\left( t\right)$ in $\mathbb {R}^3$, subject to the dynamics

1 $$\begin{aligned} \frac{dx_{i}^{N}\left( t\right) }{dt}=\sigma \partial _{t}W_{\epsilon /\xi }\left( x_{i}^{N}\left( t\right) ,t\right) +\sigma _{0}\frac{dB_{i}\left( t\right) }{dt} \end{aligned}$$

where $B_{i}\left( t\right)$ are $\mathbb {R}^3$-valued independent Brownian motions, $\xi ,\sigma ,\sigma _{0}$ are positive real numbers, $W\left( x,t\right)$ is a Gaussian $\mathbb {R}^3$-valued random field, Brownian in time

$$ \mathbb {E}\left[ W^{i}\left( x,t\right) W^{j}\left( y,s\right) \right] =C^{i,j}\left( x-y\right) t\wedge s $$

for $i,j=1,2,3$, where the covariance matrix $C\left( x\right)$ is described in Sect. 1.2 below (hence $\partial _{t}W\left( x,t\right)$ is a white noise in time) and $W_{\epsilon /\xi }\left( x,t\right) =W\left( \frac{\xi x}{\epsilon },t\right)$.

The parameter $\epsilon$ is linked to *N* by the relation

2 $$\begin{aligned} N\epsilon =1 \end{aligned}$$

which describes a specific degree of sparseness of the system (finite mean free path, in the Brownian case), described below in Sect. 1.4.1. When we take the limit as $N\rightarrow \infty$, hence $\epsilon \rightarrow 0$, the noise covariance is rescaled with $\epsilon$; hence, mathematically speaking, there would be no reason to rescale the noise by $\epsilon /\xi$ instead of just $\epsilon$ (it is a matter of redefining the covariance). But $\epsilon$, in the example described below in Sects. 1.3–1.4, is linked to the particle radius, hence the viewpoint in the scaling limit (2) is that the particle radius goes to zero meanwhile the number of particles goes to infinity (roughly speaking, particles occupy a bounded region, independently of *N*). The fact that also the noise scaling parameter $\epsilon /\xi$ goes to zero is a subsequent main assumption, not the structural link between the particles and the domain. We believe that for the interpretation of the results it is important to think that we discuss a scaling limit of a particle system, with a link (2) between number of particles and radius of interaction (a local interaction), in which there is an additional element, a turbulent fluid, with certain scaling properties related to the particle size.

When two particles $x_{i}^{N},x_{j}^{N}$, $i\ne j$, are sufficiently close, they may disappear, in a Markovian way, with rate

3 $$\begin{aligned} \frac{R_{0}}{N}\epsilon ^{-d}\theta \left( \epsilon ^{-1}\left( x_{i}^{N} -x_{j}^{N}\right) \right) \end{aligned}$$

where $\theta$ is a smooth compact support probability density function. Therefore $\epsilon$ is linked to the interaction radius of the particles. The positive number $R_{0}$ modulates the pairwise interaction rate; perhaps the most interesting regime is when $R_{0}$ is very large, case where the final average collision rate does not diverge to infinity with $R_{0}$ but converges to finite value [thanks to the assumption (2)].

We prove that the empirical distribution of the particle system converges to a function $f\left( x,t\right)$, weak solution of the equation

4 $$\begin{aligned} \partial _{t}f=\frac{1}{2}\left( \sigma ^{2}+\sigma _{0}^{2}\right) \Delta f-\overline{\mathcal {R}}f^{2}. \end{aligned}$$

The quantity $\overline{\mathcal {R}}$ is the key one to capture the average collision rate but it is important to notice, in connection with real applications, that what is usually called average collision rate, say *R*, in Physics, namely the number of collisions per unit of time and volume, is linked to $\overline{\mathcal {R}}$ by the formula

5 $$\begin{aligned} R=\frac{\overline{\mathcal {R}}}{N}n^{2} \end{aligned}$$

where *N* is the total number of particles in the system and *n* is the average number of particles per unit of volume. Indeed, *f* is the limit of a normalized empirical distribution, hence the *Nf* corresponds to *n*, then Eq. (4) gives us (up to the redistribution term $\frac{1}{2}\left( \sigma ^{2}+\sigma _{0}^{2}\right) \Delta f$), $\partial _{t} n\sim -\frac{\overline{\mathcal {R}}}{N}n^{2}$. In the abstract development of the theory we shall call $\overline{\mathcal {R}}$, sometimes, collision rate, but the correct transformation rule is (5).

In itself, Eq. (4) is a general form of quadratic limit equations, valid also under different scaling limits than 2 and different dimensions. For instance, we get the same equation if we perform a purely mean field limit with long range interaction and then send to zero the range in the final PDE. But the main feature of the scaling limit assumption 2, discovered by [13] (for the model above without the term $\sigma \partial _{t}W_{\epsilon /\xi }\left( x_{i}^{N}\left( t\right) ,t\right)$) is that $\overline{\mathcal {R}}$ is given by a non-trivial formula much different from the mean field case. One has

$$ \overline{\mathcal {R}}=R_{0}\int \theta \left( x\right) \left( 1+u\left( x\right) \right) dx $$

where *u* is the unique vanishing at infinity solution of class $C^{2}$ of the equation

6 $$\begin{aligned} \sigma _{0}^{2}\Delta u\left( x\right) +\sigma ^{2}{\operatorname {div}}\left( \omega \left( \xi x\right) \nabla u\left( x\right) \right) =R_{0}\theta \left( 1+u\right) \end{aligned}$$

where

$$ \omega \left( x\right) =I_3-C\left( x\right) . $$

Getting explicit information from this equation is not easy. But, in the limit as $R_{0}\rightarrow \infty$, one also has

$$ \overline{\mathcal {R}}\rightarrow \overline{\mathcal {R}}_{\infty }=\textrm{Cap}\left( K_{\theta },A\right) $$

where $K_{\theta }$ is the support of $\theta$ and

$$ A\left( x\right) =\sigma _{0}^{2}I_3+\sigma ^{2}\omega \left( \xi x\right) . $$

The capacity $\textrm{Cap}\left( K_{\theta },A\right)$ is defined as the infimum of

$$ \int _{\mathbb {R}^3}\nabla \phi ^{T}\left( x\right) A\left( x\right) \nabla \phi \left( x\right) dx $$

over all smooth functions $\phi \left( x\right)$, vanishing at infinity, that are larger than 1 on a neighbor of $K_{\theta }$. This characterization is more suitable for quantitative information (see Sect. 1.5). The limit as $R_{0} \rightarrow \infty$ is a very natural one, corresponding to ask for a certain coalescence, when particles meet. In the sequel and in Sect. 1.5 we shall always only use this characterization.

It is not easy to summarize in a sentence the intuition behind this result. A key of interpretation could be that Eq. (4) contains the infinitesimal generator of the “1-particle motion”, while the differential operator on the left-hand-side of (6) is like the infinitesimal generator of the difference in position between two particles. Therefore Eq. (6) contains information about the mutual behavior of different particles.

With this remark, we may explain a deep fact that, at the beginning and with the wrong eye, may even look counter-intuitive. First, notice that minimizing a quadratic form like $\int _{\mathbb {R}^3}\nabla \phi ^{T}\lambda ^{2} I_3\nabla \phi dx$ produces a result of the form $C\lambda ^{2}$. Therefore the capacity $\textrm{Cap}\left( K_{\theta },A\right)$ is (roughly speaking) linear in the size of *A*. Therefore, in the case of the matrix function $A\left( x\right) =\sigma _{0}^{2}I_3+\sigma ^{2}\omega \left( \xi x\right)$, it may look strange that it increases linearly with $\sigma _{0}^{2}$, namely with the noise term $\sigma _{0}\frac{dB_{i}\left( t\right) }{dt}$, and not linearly with the covariance $\sigma ^{2}C\left( \xi x\right)$, corresponding to the other noise term; it increases with the opposite, $\omega \left( \xi x\right) =I_3-C\left( \xi x\right)$. The deep reason stands in the “two-particle motion” of the Eq. (1). Two different particles $x_{i}^{N}\left( t\right) ,x_{j}^{N}\left( t\right)$, $i\ne j$, are driven by two independent Brownian motions $\sigma _{0}\frac{dB_{i}\left( t\right) }{dt}$ and $\sigma _{0}\frac{dB_{j}\left( t\right) }{dt}$ and by the “common noise” $\sigma \partial _{t}W_{\epsilon /\xi }\left( x,t\right)$, just evaluated at the two positions $x_{i}^{N}\left( t\right) ,x_{j} ^{N}\left( t\right)$. If the correlation of the common noise at those positions is very small, it also behaves like two independent Brownian motions, hence its effect is similar to the one of $\sigma _{0}\frac{dB_{i}\left( t\right) }{dt}$ and $\sigma _{0}\frac{dB_{j}\left( t\right) }{dt}$. And indeed, $\omega \left( \xi x\right) \sim I_3$. If the space-correlation of the common noise, on the contrary, was very strong, it should not contribute to change the distance of the two particles; and in this case $\omega \left( \xi x\right) \sim 0$. Therefore it is $\omega \left( \xi x\right)$, not $C\left( \xi x\right)$, which should be directly proportional to the collision rate. In a sense, the term $\sigma _{0}^{2}I_3$ in $A\left( x\right)$ is there because of the independence of the “internal” Brownian motions of particles, which is like a common noise with infinitesimal space correlation.

In Eq. (4), on the contrary, we have the infinitesimal generator $\frac{1}{2}\left( \sigma ^{2}+\sigma _{0}^{2}\right) \Delta$ of one-particle motion, where $C\left( \xi x\right) |_{x=0}$ plays a role. Equation (4) describes the particle density, in a sense the probability that a single particle is here or there and alive, hence governed by the one-particle motion.

The motivation of the study of this paper comes from the physical theory of aggregation of particles in turbulent fluids, see for instance [1, 5, 7, 8, 20–23, 26]. However, in such a theory, particles are inertial, inertia being quantified by means of the Stokes number *St* (defined as the ratio between the relaxation time of the particle over the relaxation time of a turbulent eddy). When inertia is large, particles feel turbulence like a light pollutant feels molecules and thus perform a Brownian motion with damping; more inertia, less collisions. On the other side of the “inertial scale of the particle”, when inertia is almost zero, particles move almost adhese to the fluid, which again leads to few collisions. It is only in the intermediate range, when inertia is still small-moderate but starts to show up, that we observe more collisions.

Our results, being based on a non-inertial model (but obtained from an inertial one by an approximation procedure, hence preserving most physical constants), meets necessarily only the case of small Stokes numbers; more precisely, the Stokes number formally disappears from the model and appears a link between two space-scales, the radius of the particle and the Kolmogorov scale. The final results will be expressed in these terms. One of the results, which shows the correctness of some approximations, is that we recover Saffman–Turner formula [25] in the case when the particle radius falls in the dissipation range of the fluid.

We complete the introduction by means of several subsections devoted mostly to physical interpretations of the model and of the results. First, we describe the covariance function $C\left( x\right)$ and the noise *W* above, Sect. 1.2. Then we describe the inertial model often considered in the literature, driven by a certain model of turbulent fluid, Sect. 1.3. In that section, we deviate from our main purpose and show briefly how Abrahamson theory arises, summary that may help to clarify that we have no chance to get such a result when we neglect inertia, see Subsect. 1.3.1. Then we describe how we derive, heuristically, a non-inertial model from the inertial one, Sect. 1.4. Finally, we interpret the main theoretical results of this paper (those summarized above), in the case of the non-inertial model with parameters $\xi ,\sigma ,\sigma _{0}$ coming from the reduction from inertial to non-inertial, which contains some Physics; see Sect. 1.5.

Let us also stress that the limit (4) is deterministic in spite of the presence of an environmental noise. Systems with environmental noise attracted a lot of attention in recent years, in particle systems scaling limit, mean field games and other theories. Usually the macroscopic dynamics is stochastic, the environmental noise is not averaged out by the scaling limit, opposite to the case of independent noise on each particle; see for instance [10] for an example in aggregation phenomena. But here we assume that the environmental noise has a scaling structure in the number of particles such that, in the limit, it behaves like independent noise on each particle—still preserving the covariance structure in the cell equation, since it is an equation for the two-point motion! An example of similar result for point vortices was obtained in [11], inspired by a comment in [12].

### Space Covariance and Noise

Let us start with the main building block (see for instance [19, Section 1.1] for more details). We assume to have a covariance matrix function $C:\mathbb {R} ^{3}\rightarrow \mathbb {R}^{3\times 3}$ having Fourier transform

$$ \widehat{C}\left( z\right) =g\left( z\right) \left( I_{3}-\frac{z\otimes z}{\left| z\right| ^{2}}\right) $$

for a non-negative real valued function *g* bounded, integrable, smooth, depending only on $\left| z\right|$; here $I_{3}$ is the identity matrix in $\mathbb {R}^{3}$. For technical reasons hopefully eliminable in future more advanced works, we assume *C* compact support, a property that can be translated into a property of analyticity and growth of *g* by a Paley–Wiener–Schwartz theorem. To this covariance matrix function *C*, it is associated a Gaussian vector valued process $W\left( x,t\right) \in \mathbb {R}^{3}$, with ${\operatorname {div}}W\left( x,t\right) =0$, that is Brownian in time and has space-covariance *C*: denoting by $\mathbb {E}$ the expected value with respect to the randomness,

$$ \mathbb {E}\left[ W^{i}\left( x,t\right) W^{j}\left( y,s\right) \right] =C^{i,j}\left( x-y\right) t\wedge s. $$

Its time derivative $\partial _{t}W\left( x,t\right)$ is a solenoidal vector valued white noise (formally speaking, the covariance of $\partial _{t}W\left( x,t\right)$ is $C\left( x-y\right) \delta \left( t-s\right)$).

One way to construct $W\left( x,t\right)$ from *C*, relevant for the mathematical analysis below and possibly for numerical simulations, is the following one. To *C* we associate the covariance operator $\mathcal {C}$ in the space $L_{s}^{2}\left( \mathbb {R}^{3},\mathbb {R}^{3}\right)$ of solenoidal vector fields on $\mathbb {R}^{3}$; this operator is positive selfadjoint and compact, hence it has a spectral decomposition $\mathcal {C} v=\sum _{k=0}^{\infty }\lambda _{k}\left\langle v,e_{k}\right\rangle _{L^{2} }e_{k}$, $v\in L_{s}^{2}\left( \mathbb {R}^{3},\mathbb {R}^{3}\right)$, where $\left( e_{k}\right) _{k\in \mathbb {N}}$ is a complete orthonormal system of $L_{s}^{2}\left( \mathbb {R}^{3},\mathbb {R}^{3}\right)$ and $\lambda _{k} \ge 0$ for every $k\in \mathbb {N}$. Taken a sequence $\left( W_{k}\left( t\right) \right) _{k\in \mathbb {N}}$ of independent scalar Brownian motions, we define

7 $$\begin{aligned} W\left( x,t\right) =\sum _{k=0}^{\infty }\lambda _{k}e_{k}\left( x\right) W_{k}\left( t\right) . \end{aligned}$$

From the assumption on the Fourier transform of *C*, one can prove that $x\mapsto W\left( x,t\right)$ is an isotropic random field. In particular $C\left( x\right)$ is a function of $\left| x\right|$ only; and one has $C\left( 0\right) =\frac{1}{3}\left\| g\right\| _{L^{1}}I_{3}$ where $\left\| g\right\| _{L^{1}}=\int _{\mathbb {R}^{3}}g\left( x\right) dx$. We assume $\frac{1}{3}\left\| g\right\| _{L^{1}}=1$, hence

$$ C\left( 0\right) =I_{3}. $$

### Inertial Model

Behind the non-inertial model of this paper there is an inertial one that we describe now. We use new notations, to avoid confusion.

Consider *N* particles in $\mathbb {R}^{3}$, with positions and velocities $\left( x_{1}^{N}\left( t\right) ,v_{1}^{N}\left( t\right) \right)$,..., $\left( x_{N}^{N}\left( t\right) , v_{N}^{N}\left( t\right) \right)$, subject to a turbulent fluid by Stokes law:

$$\begin{aligned} \frac{dx_{i}^{N}\left( t\right) }{dt}&=v_{i}^{N}\left( t\right) \\ \frac{dv_{i}^{N}\left( t\right) }{dt}&=-\frac{1}{\tau _{P}}\left( v_{i}^{N}\left( t\right) u (x_i^N(t), t) \right) +\frac{u_{P}}{\sqrt{\tau _{P}}}\frac{dB_{i}\left( t\right) }{dt} \end{aligned}$$

where $u\left( x,t\right)$ is a space-time stationary Gaussian vector random field, divergence free, with

$$ \mathbb {E}\left[ u_{i}\left( x,t\right) u_{j}\left( y,s\right) \right] =e^{-\frac{\left| t-s\right| }{\tau _{\eta }}}P_{ij}\left( x-y\right) $$

for $i,j=1,2,3$. With this first identity we have specified the relaxation time $\tau _{\eta }$ of the turbulent fluid. Then we want to specify, by a proper choice of $P\left( x\right)$, that the fluid is very correlated when $\left| x-y\right| \le \eta$, interpreted as Kolmogorov scale (below which dissipation dominates and the velocity field $u\left( x,t\right)$ is smooth and mildly varying), then less and less correlated as $\left| x-y\right|$ increases above $\eta$. One way, accepting moreover isotropy and other idealizations, is to assume

$$ P\left( x\right) =u_{\eta }^{2}C\left( \frac{L}{\eta }x\right) $$

where $u_{\eta }^{2}$ is a measure of the turbulent kinetic energy, $C\left( x\right)$, $\left( e_{k},\lambda _{k}\right) _{k\in \mathbb {N}}$ and $W\left( x,t\right)$ are as in the previous section and *L* is a macroscopic length scale. For simplicity (although it is not mandatory) we choose $u_{\eta }=\frac{\eta }{\tau _\eta }$. If Kolmogorov scale is adopted, we may use the formulae $\eta =\left( \frac{\nu ^{3}}{\varepsilon }\right) ^{1/4}$, $\tau _{\eta }=\left( \frac{\nu }{\varepsilon }\right) ^{1/2}$, $u_{\eta }=\left( \nu \varepsilon \right) ^{1/4}$, where $\nu$ is the kinematic viscosity of the fluid and $\varepsilon$ the average dissipation energy rate.

Let us also introduce the matrix

$$ D\left( x\right) =u_{\eta }^{2}\left( I_3-C\left( \frac{L}{\eta }x\right) \right) . $$

One has

$$\begin{aligned} D_{ij}\left( x-y\right)&=u_{\eta }^{2}\delta _{ij}-P_{ij}\left( x-y\right) \\&=\frac{1}{2}\mathbb {E}\left[ \left( u_{i}\left( x,t\right) -u_{i}\left( y,t\right) \right) \left( u_{j}\left( x,t\right) -u_{j}\left( y,t\right) \right) \right] \end{aligned}$$

so it is related to the second order structure function, in particular to the quantity $\left\langle \left| \Delta _{\left| x-y\right| }u\right| ^{2}\right\rangle$ anticipated in the Abstract, defined as the square-average of the increment of random velocity field *u* between points at distance $\left| x-y\right|$:

$$ \left\langle \left| \Delta _{\left| x-y\right| }u\right| ^{2}\right\rangle =2\textrm{Tr}D\left( x-y\right) . $$

One way to “realize” such field $u\left( x,t\right)$ is by taking $u\left( x,t\right) =u_{\eta }Z_{\eta }\left( x,t\right)$ where $Z_{\eta }\left( x,t\right)$ is a stationary solution of

$$ \partial _{t}Z_{\eta }\left( x,t\right) =-\frac{1}{\tau _{\eta }}Z_{\eta }\left( x,t\right) +\frac{1}{\sqrt{\tau _{\eta }}}\partial _{t}W_{\eta /L}\left( x,t\right) . $$

One can easily show that

8 $$\begin{aligned} \mathbb {E}\left[ Z_{\eta }^{i}\left( x,t\right) Z_{\eta }^{j}\left( y,s\right) \right] =e^{-\frac{\left| t-s\right| }{\tau _{\eta }} }C^{i,j}\left( \frac{L}{\eta }\left( x-y\right) \right) . \end{aligned}$$

The process $Z_{\eta }\left( x,t\right)$ has the same space-covariance as $W_{\eta /L}\left( x,t\right)$, but it is stationary with time-correlation $\tau _{\eta }$. We keep the notation $\eta /L$ for $W_{\eta /L}$ for comparison with $W_{\epsilon /\xi }$ of Sect. 1.1, but drop *L* from all other notations like $u_{\eta },\tau _{\eta },Z_{\eta }$ for simplicity of notations.

This completes the description of the fluid velocity field. Concerning the particles, we have assumed in the model above that they have an inertial motion, with relaxation time $\tau _{P}$ and Stokes law $-\frac{1}{\tau _{P} }\left( v_{i}^{N}\left( t\right) -u_{\eta }Z_{\eta }\left( x_{i}^{N}\left( t\right) ,t\right) \right)$ linking them to the fluid motion, plus a very small “internal” noise $\frac{u_{P}}{\sqrt{\tau _{P}}}\frac{dB_{i}\left( t\right) }{dt}$, phenomenologically taking into account particle geometry and internal degrees of freedom interacting with the environment in a complex way. Neglecting the fluid, the standard deviation of $v_{i}^{N}\left( t\right)$ would be proportional to $u_{P}$, providing a phenomenological interpretation of the parameter $u_{P}$; that we shall assume much smaller than all other contributions, just needed to mathematical reasons of “diffusion regularization”.

Particles have an interaction radius *a*: when the centers have distance less or equal to 2*a*, they undergo a collision which may produce coalescence of the particles, with a certain rate of collision (since we do not develop the full inertial theory, we skip the precise description of the rate). We assume to work in a fluid-particle regime where $\eta$ and *a* are roughly comparable, maybe differing multiplicatively by a large or small length-scale ratio

$$ \xi _{0}=\frac{a}{\eta } $$

but not being different infinitesimal orders when we take the limit $a\rightarrow 0$. Therefore we assume $\xi$ finite in the limit $a\rightarrow 0$ and we can write the covariance $C\left( \frac{L}{\eta }x\right)$ of the noise in the form

$$ C\left( \frac{\xi _{0}L}{a}x\right) . $$

This model, heuristically speaking, captures a number of properties of aggregation in turbulent fluids, the we describe in the following three cases $1_{iner},2_{iner},3_{iner}$ (certainly non exhausting the important informations on the problem, but useful for the conceptualizations made later on).

$1_{iner}$: Case $\tau _{P}\ll \tau _{\eta }$. In this case, particles are almost adhese to the fluid and thus do not collide so often. This case is classically covered by Saffman–Turner law $\overline{\mathcal {R}}\sim a^{3}$ (up to constants involving the factor $\left( \frac{\varepsilon }{\nu }\right) ^{1/2}$) [25].

$2_{iner}$: Case $\tau _{P}\sim \tau _{\eta }$ (up to finite constants). In this case, particles are less adhese, with some (but not large) inertia. More collisions are observed.

$3_{iner}$: Case $\tau _{P}\gg \tau _{\eta }$. In this case inertia dominates more and more as *St* increases; the behavior of particles is similar to independent Brownian motions, but with inertia. More inertia produces less collisions, hence the mean collision rate decreases with *St*. See Subsect. 1.3.1 below for additional informations (like Abrahamson law $\frac{u_{\eta }}{\sqrt{St}}$).

In this paper the condition $\tau _{P}\ll 1$ is essential for the non-inertial approximation. Although a priori this could still allow us to consider $\tau _{P}\gg \tau _{\eta }$, mathematically the information about $\tau _{P}$ is lost and our results do not cover such a case. Our paper is restricted to cases $1_{iner}$ and $2_{iner}$.

However, even with the restriction to $1_{iner}$ and $2_{iner}$, we do not fully recover the rapid increase of mean collision rate happening at some Stokes level, when we pass from $1_{iner}$ to $2_{iner}$. Our theory gives only a relatively smooth transition from $1_{iner}$ to $2_{iner}$. We presume it is due to the homogeneous Gaussian approximation of the velocity field, which lacks very important concentration effects of the density of particles.

#### The Case $\tau _{P}\gg \tau _{\eta }$

Let us expand this case for comparison, case unfortunately not covered by the theory of this paper.

In this case, from the observational viewpoint of a particle, $Z_{\eta }\left( x,t\right)$ is like a white noise, say of the form $\sqrt{\tau _{\eta }} d\beta _{i}\left( t\right)$ (see (10) below), hence the velocity $v_{i}^{N}\left( t\right)$ behaves like the solution of a damped Langevin equation with double noise:

$$ \frac{dv_{i}^{N}\left( t\right) }{dt}=-\frac{1}{\tau _{P}}v_{i}^{N}\left( t\right) +\frac{u_{\eta }\sqrt{\tau _{\eta }}}{\tau _{P}}\frac{d\beta _{i}\left( t\right) }{dt}+\frac{u_{P}}{\sqrt{\tau _{P}}}\frac{dB_{i}\left( t\right) }{dt}. $$

For notational and conceptual simplicity, let us neglect the very small contribution $\frac{u_{P}}{\sqrt{\tau _{P}}}\frac{dB_{i}\left( t\right) }{dt}$ and rewrite the main coefficient as

9 $$\begin{aligned} \frac{dv_{i}^{N}\left( t\right) }{dt}=-\frac{1}{\tau _{P}}v_{i}^{N}\left( t\right) +\frac{1}{\sqrt{\tau _{P}}}\frac{u_{\eta }}{\sqrt{St}}d\beta _{i}\left( t\right) \end{aligned}$$

where the Stokes number is defined as

$$ St=\frac{\tau _{P}}{\tau _{\eta }}. $$

For large *St*, the inertia dominates the picture and collisions are not so frequent. To see this from Eq. (9), first let us assume that, for two different particles $i\ne i$, $\beta _{i}\left( t\right)$ and $\beta _{j}\left( t\right)$ are independent Brownian motions; this is a reasonable approximation, using the rough argument that a strong order between the time scales $\tau _{P}\gg \tau _{\eta }$ corresponds also to a strong order between the length scales $a\gg \eta$. Hence, for the difference $v_{ij} ^{N}\left( t\right) =v_{i}^{N}\left( t\right) -v_{j}^{N}\left( t\right)$ we have exactly the same Eq. (9), just with a factor $\sqrt{2}$ in front of the noise. The standard deviation of a stationary solution is proportional to $\frac{u_{\eta }}{\sqrt{St}}$, hence (up to a multiplicative factor) we infer that the average collision rate is $\frac{u_{\eta }}{\sqrt{St}}$. This is the famous Abrahamson law [1]. Unfortunately, this law is lost in the non-inertial approximation below (in spite of several attempts; it seems an intrinsic weakness of the non-inertial model, for the obvious reason that it corresponds to an approximation around $\tau _{P}=0$). Similarly, we lose the change of monotonicity which is known from theories and experiments, namely the decrease of the average collision rate in *St* for large *St*, after its increase for smaller *St*.

### Reduction to a Non-inertial Model

The model above, suitable for numerical simulations, is too difficult at present for a rigorous mathematical investigation of the limit as $N\rightarrow \infty$. We apply two simplifications, based on the fact that both $\tau _{P}$ and $\tau _{\eta }$ are very small in practical examples. We have a first approximation

10 $$\begin{aligned} Z_{\eta }\left( x,t\right) dt\sim \sqrt{\tau _{\eta }}\partial _{t}W_{\eta /L}\left( x,t\right) \end{aligned}$$

coming from the equation

$$ -\tau _{\eta }\partial _{t}Z_{\eta }\left( x,t\right) =Z_{\eta }\left( x,t\right) -\sqrt{\tau _{\eta }}\partial _{t}W_{\eta /L}\left( x,t\right) $$

as $\tau _{\eta }\rightarrow 0$; and a second approximation

$$ v_{i}^{N}\left( t\right) \sim u_{\eta }Z_{\eta }\left( x_{i}^{N}\left( t\right) ,t\right) +u_{P}\sqrt{\tau _{P}}\frac{dB_{i}\left( t\right) }{dt} $$

coming from the equation

$$ -\tau _{P}\frac{dv_{i}^{N}\left( t\right) }{dt}=v_{i}^{N}\left( t\right) -u_{\eta }Z_{\eta }\left( x_{i}^{N}\left( t\right) ,t\right) -u_{P} \sqrt{\tau _{P}}\frac{dB_{i}\left( t\right) }{dt} $$

as $\tau _{P}\rightarrow 0$. Inserting further the first approximation in the second one, we get

$$ v_{i}^{N}\left( t\right) \sim u_{\eta }\sqrt{\tau _{\eta }}\partial _{t} W_{\eta /L}\left( x_{i}^{N}\left( t\right) ,t\right) +u_{P}\sqrt{\tau _{P} }\frac{dB_{i}\left( t\right) }{dt}. $$

Substituting this into the first equation we get

11 $$\begin{aligned} \frac{dx_{i}^{N}\left( t\right) }{dt}=u_{\eta }\sqrt{\tau _{\eta }}\partial _{t}W_{\eta /L}\left( x_{i}^{N}\left( t\right) ,t\right) +u_{P}\sqrt{\tau _{P}}\frac{dB_{i}\left( t\right) }{dt} \end{aligned}$$

which is a closed equation. Understanding the degree of these approximations and their relative importance is very difficult and much beyond the scope of this paper.

Concerning coalescence, first we oversimplify certain aspects of the problem (to focus on difficult others) and therefore assume that particles, after coalescence, “exit the system”. Namely we focus only on the number of particles of radius *a* and neglect not only what happens to the result of coalescence but also the important fact that, in reality, larger particles produced by coalescence usually may coalesce also with the smaller particles of radius *a*, contributing to the decrease of their number. But all these elements may be accounted for by the formalism of Smoluckowski equations, which we omit here. By the way, with the present simplified formalism the model may be applied to fragmentation too.

Concerning the collision rate between two particles $x_{i}^{N}$ and $x_{j} ^{N}$, $i\ne j$, in the case of the non-inertial model a natural choice is the form

12 $$\begin{aligned} \frac{R_{0}}{N}a^{-3}\theta _{0}\left( a^{-1}\left( x_{i}^{N}-x_{j} ^{N}\right) \right) \end{aligned}$$

where $R_{0}$ has dimension $\left[ L\right] ^{3}\left[ T\right] ^{-1}$ to give dimension $\left[ T\right] ^{-1}$ to the rate. Here $\theta _{0}$ is a smooth non negative function, with $\int \theta _{0}\left( x\right) dx=1$, roughly equal to $\frac{3}{4\pi }$ in the ball $B\left( 0,1\right)$, and equal to zero outside.

Before we close this section, let us mention heuristically what the previous non-inertial model may hope to tell us.

We mention this fact, along with the next one, to say that the general formula (14) or the previous ones of this section, more precise and rigorous, are only a first step based on an alternative methodology, still far from providing a good understanding of the small-to-moderate range of particle radius. Particles, just described by their position $x_{i}^{N}\left( t\right)$, are driven only by two Brownian sources, $W_{\eta /L}\left( x_{i}^{N}\left( t\right) ,t\right)$ and $B_{i}\left( t\right)$. Thus $x_{i}^{N}\left( t\right)$ behaves roughly like a Brownian motion (not precisely, since $W_{\eta /L}\left( x_{i}^{N}\left( t\right) ,t\right)$ depends on the particle position). Particles will collide, because of the random motion, with intensity increasing with the coefficient $u_{\eta } \sqrt{\tau _{\eta }}$ (also with $u_{P}\sqrt{\tau _{P}}$, but we assume $u_{P}$ much smaller than all other constants).

$1_{non-iner}$: case $a\ll \eta$. The process $W_{\eta /L}\left( x,dt\right)$ is very correlated below the Kolmogorov scale $\eta$, hence particles which have radius *a* much smaller than the Kolmogorov scale and are very close each other, feel the same random impulses, thus the random strength of $W_{\eta /L}\left( x,t\right)$ on them is very reduced, it does not put them closer; they travel parallely, so to speak. Therefore aggregation, which increases with $u_{\eta }\sqrt{\tau _{\eta }}$, is depleted if particles have a radius *a* much smaller than $\eta$.

$2_{non-iner}$: case $a\uparrow \eta$. Both obstructions discussed in $1_{non-iner}$ are progressively relaxed when *a* increases to $\eta$. Therefore the mean collision rate should monotonically increase, when $a\uparrow \eta$.

Let us now make precise the scaling between *N* and *a* chosen here, which is a critical issue. We separate it into a subsection for the reader’s convenience.

#### The Mean Free Path Regime

We assume the initial positions of the particles located in a unitary volume, approximately, so the same is true in a finite time interval approximately. Therefore, if we increase *N*, particles are more packed together, not just distributed in a larger volume; and their radius *a* is assumed to go to zero, if we increase *N* towards infinity, to get a sharp mathematical result. The link between *N* and *a* will be a finite number (finite in the limit when $N\rightarrow \infty$ and $a\rightarrow 0$).

13 $$\begin{aligned} Na=\rho _{0}. \end{aligned}$$

Here $\rho _{0}$ is a given positive number with dimension $\left[ L\right]$: It is a “volume-occupation-constant”: the volume occupied by the balls of radius *a* centered at the particle positions is

$$ {\text {volume of particles }}\sim \frac{4\pi }{3}Na^{3}=\frac{4\pi }{3} \rho _{0}a^{2} $$

hence the volume of particles linearly increases with $\rho _{0}$.

Since *a* is small, this volume is very small, hence we are talking about a very sparse regime. The intuition then is of a very sparse collection of droplets. However, they perform, roughly speaking, Brownian motions (mostly due to the turbulent contribution $u_{\eta }\sqrt{\tau _{\eta }}\partial _{t}W_{\eta /L}\left( x_{i}^{N}\left( t\right) ,t\right)$), hence each one of their centers explores in unitary time a subset of $\mathbb {R}^{3}$ which has Hausdorff dimension 2 (with 2-dimensional Hausdorff measure zero). Hence, a ball of radius *a* centered at such a point, explores a set $A\subset \mathbb {R}^{3}$ (called the Brownian sausage) having volume roughly of size *a*, up to multiplication by the parameters of the Brownian motion. During the excursion performed in a unit of time, the particle meets all particles that (with their radius) intersect *A*. If the particles are roughly uniformly distributed, then the number of particles which intersect *A* is of order $N\cdot \textrm{Vol}(A)$, namely *Na*, which is a finite number $\rho _{0}$. This implies that each particle has a finite number of encounters in the unit of time. This is the so-called mean free path regime.

This is the only regime where we can find non trivial limit results. Indeed, when $Na\rightarrow \infty$ we have a mean field result, since each particle would meet infinitely many others in a unit of time; one can show that the result is simply

$$ \overline{\mathcal {R}}=R_{0} $$

which is a trivial and physically wrong result. On the contrary, for $Na\rightarrow 0$, we would not observe sufficient interactions in the scaling limit; the average collision rate $\overline{\mathcal {R}}$ would vanish.

Therefore we investigate a *non-mean-field regime*, and the mathematical correction in the final formulae coming from this choice is essential.

### Application of Our Results and Their Physical Interpretation

#### Conversion of the Notations

Let us first summarize the notation of the concrete example of Sect. 1.4 in comparison with the abstract notations of Sect. 1.1. The concrete dynamics of particles is given by equation (11), the rate of collision by (12) and the scaling regime by (13). In the abstract formulation of Sect. 1.1 the dynamics is given by (1), the rate of collision by (3) and the scaling by (2). Therefore the conversion table is (on the left the abstract notations, on the right those of the concrete example:

$$\begin{aligned} \epsilon&=\frac{a}{\rho _{0}}\\ \theta \left( x\right)&=\rho _{0}^{-3}\theta _{0}\left( \rho _{0} ^{-1}x\right) \\ \sigma&=u_{\eta }\sqrt{\tau _{\eta }}\\ \sigma _{0}&=u_{P}\sqrt{\tau _{P}}\\ \xi&=\frac{L}{\rho _{0}}\frac{a}{\eta } \end{aligned}$$

and recall

$$\begin{aligned} P\left( x\right)&=u_{\eta }^{2}C\left( \frac{L}{\eta }x\right) =u_{\eta }^{2}C\left( \frac{\xi _{0}L}{a}x\right) \\ D\left( x\right)&=u_{\eta }^{2}\left( I_3-C\left( \frac{L}{\eta }x\right) \right) \\ R&=\frac{\overline{\mathcal {R}}}{N}n^{2} \end{aligned}$$

(the first identity comes from comparison of the scaling limit assumption; then the second identity from comparison of the collision rates; the last identity is chosen in order to have $W_{\epsilon /\xi }=W_{\eta /L}$, recall the definition $W_{\epsilon /\xi }\left( x,t\right) =W\left( \frac{\xi }{\epsilon }x,t\right)$ from Sect. 1.1 and $W_{\eta /L}\left( x,t\right) =W\left( \frac{L}{\eta }x,t\right)$ from Sect. 1.4).

#### The Result in the Notations of Sect. 1.4

The normalized empirical measure of the particle system converges to a density *f* satisfying

$$ \partial _{t}f=\frac{1}{2}\left( u_{\eta }^{2}\tau _{\eta }+u_{P}^{2}\tau _{P}\right) \Delta f-\overline{\mathcal {R}}f^{2} $$

where

$$ \overline{\mathcal {R}}=R_{0}\int \rho _{0}^{-3}\theta _{0}\left( \rho _{0} ^{-1}x\right) \left( 1+u\left( x\right) \right) dx $$

where *u* is the unique vanishing at infinity solution of class $C^{2}$ of the equation

$$ u_{P}^{2}\tau _{P}\Delta u\left( x\right) +u_{\eta }^{2}\tau _{\eta }{\operatorname {div}}\left( \left( I_3-C\left( \frac{L}{\rho _{0}}\frac{a}{\eta }x\right) \right) \nabla u\left( x\right) \right) =R_{0}\rho _{0}^{-3}\theta _{0}\left( \rho _{0}^{-1}x\right) \left( 1+u\right) $$

namely

$$ u_{P}^{2}\tau _{P}\Delta u\left( x\right) +\tau _{\eta }{\operatorname {div}} \left( D\left( \frac{a}{\rho _{0}}x\right) \nabla u\left( x\right) \right) =R_{0}\rho _{0}^{-3}\theta _{0}\left( \rho _{0}^{-1}x\right) \left( 1+u\right) . $$

In the limit as $R_{0}\rightarrow \infty$, one has

$$ \overline{\mathcal {R}}\rightarrow \overline{\mathcal {R}}_{\infty }= \textrm{Cap}\left( K_{\theta _{0}\left( \rho _{0}^{-1}\cdot \right) },A\right) = \textrm{Cap}\left( B\left( 0,\rho _{0}\right) ,A\right) $$

where $K_{\theta _{0}\left( \rho _{0}^{-1}\cdot \right) }=B\left( 0,\rho _{0}\right)$ is the support of $\theta _{0}\left( \rho _{0}^{-1}\cdot \right)$, *A* is the matrix function $A\left( x\right) =u_{P}^{2}\tau _{P}I_3 +\tau _{\eta }D\left( \frac{a}{\rho _{0}}x\right)$ and the capacity $\textrm{Cap}\left( B\left( 0,\rho _{0}\right) ,A\right)$ is defined as the infimum of $\int _{\mathbb {R}^3}\nabla \phi ^{T}\left( x\right) A\left( x\right) \nabla \phi \left( x\right) dx$ over all smooth functions $\phi \left( x\right)$, vanishing at infinity, that are larger than 1 on a neighbor of $B\left( 0,\rho _{0}\right)$.

A very heuristic computation in spherical coordinates, for the simplified example of minimizing $\int _{B\left( 0,\rho _{0}\right) ^{c}}\nabla \phi ^{T}\left( x\right) I_3\nabla \phi \left( x\right) dx$ over all smooth functions $\phi \left( x\right)$, vanishing at infinity, equal to 1 on $\partial B\left( 0,\rho _{0}\right)$, shows that the minimizer is not so different from the radial function linearly decaying to zero on $r\in \left[ \rho _{0},2\rho _{0}\right]$, with derivative modulus $\frac{1}{\rho _{0}}$. The minimal value is roughly equal to $\rho _{0}$, up to a constant (this is the well known result on the electric capacity of a sphere). If, in place of $A\left( x\right) =I_3$ we take a slowly varying function with radial symmetry, a rough approximation of the infimum is $\frac{1}{3}\textrm{Tr}A\left( \rho _{0}\right) \rho _{0}$, since *A* matters only when $r\in \left[ \rho _{0},2\rho _{0}\right]$. Therefore, the infimum in our case of interest can be approximated by

$$\begin{aligned} \frac{1}{3}\textrm{Tr}A\left( \rho _{0}\right) \rho _{0}&=u_{P}^{2}\tau _{P}\rho _{0}+\tau _{\eta }\frac{1}{3}\textrm{Tr}D\left( a\right) \rho _{0}\\&=u_{P}^{2}\tau _{P}\rho _{0}+\tau _{\eta }\frac{1}{6}\left\langle \left| \Delta _{a}u\right| ^{2}\right\rangle \rho _{0} \end{aligned}$$

that (to simplify the formulae) we replace by

$$ \tau _{\eta }\left\langle \left| \Delta _{a}u\right| ^{2}\right\rangle \rho _{0} $$

since $u_{P}^{2}$ is very small and we have also missed other multiplicative constants like $\frac{1}{6}$.

Therefore, taking into account $R=\frac{\overline{\mathcal {R}}}{N}n^{2}$ and $Na=\rho _{0}$, we get

14 $$\begin{aligned} R\sim \tau _{\eta }\left\langle \left| \Delta _{a}u\right| ^{2} \right\rangle a\cdot n^{2} \end{aligned}$$

as stated in the Abstract. Then, in particular, if we accept Kolmogorov time scale $\tau _{\eta }\sim \left( \frac{\nu }{\varepsilon }\right) ^{1/2}$ and we assume that *a* is in the dissipative range where

$$ \left\langle \left| \Delta _{a}u\right| ^{2}\right\rangle \sim \left\langle \left| \nabla u\right| ^{2}\right\rangle a^{2}=\left( \frac{\varepsilon }{\nu }\right) a^{2} $$

(because of the balance relation $\nu \left\langle \left| \nabla u\right| ^{2}\right\rangle =\varepsilon$), we get Saffman–Turner formula

$$\begin{aligned} R&\sim \left( \frac{\nu }{\varepsilon }\right) ^{1/2}\left( \frac{\varepsilon }{\nu }\right) a^{2}a\cdot n^{2}\\&=\left( \frac{\varepsilon }{\nu }\right) ^{1/2}a^{3}\cdot n^{2}. \end{aligned}$$

The Saffman–Turner regime was already well accepted in the Physical literature, hence we just confirm the result in a more complex way than the original paper [25]. Part of our procedure is rigorous, but many steps were hand waving approximations (very reasonable), hence we cannot claim that we have rigorously proved Saffman–Turner result from first principle. We have only provided an alternative derivation.

The final formula (14), besides being correct in the dissipation range, provides a new view on the behavior for slightly larger *a*. We should however remember that we took a limit as $\tau _{P}\rightarrow 0$, hence larger *a* compared to $\eta$ are meaningful only in few physical examples, precisely when the density of a particle in much less than the density of the fluid, which is not true for rain droplets in air or small dust clusters in star dust; maybe it may find more applications in fragmentation processes. If $\tau _{P}\rightarrow 0$ by *a* is in the inertial range, where

$$ \left\langle \left| \Delta _{a}u\right| ^{2}\right\rangle \sim \varepsilon ^{2/3}a^{2/3} $$

according to K41 theory (otherwise one should include intermittent corrections) one get (with the same arguments above)

$$ R\sim \nu ^{1/2}\varepsilon ^{1/6}a^{5/3}\cdot n^{2} $$

which is not a common result and not of easy interpretation. More generally, even if we do not use the Kolmogorov scaling law of structure function, it is clear that $\left\langle \left| \Delta _{a}u\right| ^{2}\right\rangle$, which starts as $a^{2}$ for very small *a*, tends to saturate for larger *a*, providing an “S-shape”. This is very reasonable. It is a monotone behavior with saturation. There is no chance however to see changes of monotonicity, with the given approximations.

##### Remark 1

Even if limited to small Stokes numbers, in principle it should be possible to incorporate in a precise mathematical model some ingredient which produces particle concentration, a phenomenon that is observed and is among those responsible for a strong increase of collision rate, when certain parameters increase with respect to Saffman–Turner regime. This remains an open problem in our framework, perhaps the most interesting one. See [4] for results in this direction.

## Statement of Main Result

In this section, we start by recapping our model with additional details (that were omitted in the Introduction due to their technical nature), and then we state our main Theorem 6.

About the covariance function *C*(*x*), recall that by construction this function is radially symmetric, $C(0)=I_3$ and $\omega (x) = I_3-C(x)$ is nonnegative definite. The latter property is due to $\omega (x) = \frac{1}{2}\sum _{k\in \mathbb {N}}\big (e_k(0)-e_k(x)\big )^{\otimes 2}$. We also have

15 $$\begin{aligned} C(x-y)=\sum _{k\in \mathbb {N}}\lambda _ke_{k}\left( x\right) \otimes \lambda _ke_{k}\left( y\right) , \end{aligned}$$

where we recall that our Gaussian random field *W*(*x*, *dt*) can be written as a series (7). Henceforth, we will write

$$\begin{aligned} C_\epsilon (x)= C\Big (\frac{x}{\epsilon }\Big ), \quad e_k^\epsilon (x) = e_k\Big (\frac{x}{\epsilon }\Big ). \end{aligned}$$

By the series representation of $W_{\epsilon /\xi }(x, dt)$, we can rewrite the dynamics of the particle system (1) as

16 $$\begin{aligned} dx_i^N(t)&=\sigma \sum _{k\in \mathbb {N}} \lambda _ke_{k}^{\epsilon }\left( \xi x_i^N(t)\right) \circ \, dW_k(t)+\sigma _0\, dB_i(t),\quad i\in \mathcal {N}(t), \end{aligned}$$

where $\circ$ denotes Stratonovich integration. We make this choice in accordance with Wong-Zakai principle. Due to $e_k^\epsilon$ being divergence free, it turns out that in our setting it coincides with Itô integration, since the Stratonovich-to-Itô corrector is zero. $\mathcal {N}(t)\subset \{1,2,...,N\}=\mathcal {N}(0)$ is the (random) index set of active particles at time *t*.

Let us formally state our model: Given a probability space $(\Omega , \mathcal {F}, \mathbb {P})$, for every $N\in \mathbb {N}$, we consider a system of initially *N* particles evolving in $\mathbb {R}^3$, whose positions $x_i(0)$ at time $t=0$ are independent and identically distributed (i.i.d) with probability density $f_0(x)$. Particles are enumerated from $i=1,2,...,N$ at the beginning, and their indices are fixed once and for all. As long as a particle $i\in \{1,2,...,N\}$ is alive, its spatial position $x_i^N(t)\in \mathbb {R}^3$ follows (16). Any pair of distinct particles (*i*, *j*), $i\ne j$, may coalesce and both disappear, with a stochastic rate (3) which we denote by $r_{ij}^N$. When a particle is no longer active, we assign it a “cemetery” state $\emptyset$ which is absorbing, and thus the cardinality of active particles in the system at $t>0$ are less or equal than *N* and non-increasing in time.

We impose the following assumption on the initial density $f_0(x)$ of an individual particle.

### Condition 2

There exist finite constants $L_0$ and $\Gamma$ such that $f_0$ is supported in (the Euclidean ball of radius $L_0$ around the origin) $B(0,L_0)\subset \mathbb {R}^3$, and $f_0(x)\le \Gamma$ for all $x\in \mathbb {R}^3$. In particular, $f_0\in L^1\cap L^\infty (\mathbb {R}^3; \mathbb {R}_+)$.

The probability space is endowed with the canonical filtration

$$\begin{aligned} \mathcal {G}_t:=\sigma \left\{ \{W_k(s)\}_{k\in \mathbb {N}}, \{B_i(s)\}_{i=1}^\infty , s\le t, \{x_i(0)\}_{i=1}^\infty \right\} , \quad t\ge 0. \end{aligned}$$

Denoting by $\zeta$ a generic configuration of the particle system, i.e. $\zeta =(x_1,x_2,...,x_N)\in \left( \mathbb {R}^3\cup \{\emptyset \}\right) ^N$, and by $\zeta _t^N$ the configuration of the particle system at time *t*, $(\zeta _t^N)_{t\ge 0}$ is a Markov process with infinitesimal generator

17 $$\begin{aligned} \mathcal L^NF(\zeta )&=\mathcal {L}^N_DF(\zeta )+\mathcal {L}^N_JF(\zeta ) \end{aligned}$$

18 $$\begin{aligned} \mathcal {L}^N_DF(\zeta )&:=\frac{1}{2}\sigma ^2\sum _{k\in \mathbb {N}}\sum _{i,j\in \mathcal {N}(\zeta )}\lambda _k^2e_k^\epsilon (\xi x_i)\cdot \nabla _{x_i} \big (e_k^\epsilon (\xi x_j)\nabla _{x_j}F(\zeta )\big )+\frac{1}{2}\sigma _0^2\sum _{i\in \mathcal {N}(\zeta )}\Delta _{x_i}F(\zeta )\nonumber \\&=\frac{1}{2}\sigma ^2\sum _{k\in \mathbb {N}}\sum _{i,j\in \mathcal {N}(\zeta )}\lambda _k^2\nabla _{x_i}\cdot \big (e_k^\epsilon (\xi x_i)\otimes e_k^\epsilon (\xi x_j)\nabla _{x_j}F(\zeta )\big )+\frac{1}{2}\sigma _0^2\sum _{i\in \mathcal {N}(\zeta )}\Delta _{x_i}F(\zeta ) \end{aligned}$$

19 $$\begin{aligned}&=\frac{1}{2}\sigma ^2\sum _{i,j\in \mathcal {N}(\zeta )}\nabla _{x_i}\cdot \big (C_\epsilon (\xi (x_i-x_j))\nabla _{x_j}F(\zeta )\big )+\frac{1}{2}\sigma _0^2\sum _{i\in \mathcal {N}(\zeta )}\Delta _{x_i}F(\zeta )\nonumber \\ \mathcal {L}^N_JF(\zeta )&:=\frac{1}{2}\sum _{i\in \mathcal {N}(\zeta )}\sum _{j\in \mathcal {N}(\zeta ): j\ne i}r_{ij}^N\left[ F(\zeta ^{-ij})-F(\zeta )\right] . \end{aligned}$$

Here, $F: \left( \mathbb {R}^3\cup \{\emptyset \}\right) ^N\rightarrow \mathbb {R}$ is a functional on the configuration space, $\nabla _{x_i}$ denotes partial gradient with respect to variable $x_i\in \mathbb {R}^3$ and similarly $\nabla _{x_i}\cdot$ denotes partial divergence. We denote the index set of active particles in $\zeta$ by

$$\begin{aligned} \mathcal {N}(\zeta )&:=\{i: x_i\in \zeta , x_i\ne \emptyset \}\subset \{1,2,...,N\}, \end{aligned}$$

whereas $\zeta ^{-ij}$ is a configuration obtained from $\zeta$ by setting its *i*-th and *j*-th slots to $\emptyset$, and keeping other slots unchanged. The 1/2 in (19) is due to double counting in the double sum of an annihilation event. We have used the fact that $e_k^\epsilon (x)$ are divergence free when calculating $\mathcal {L}^N_D$ at step (18). Our assumptions actually imply [see (49)] that $\mathcal {L}^N_DF(\zeta )$ can be equivalently written as non-divergence form

$$\begin{aligned} \mathcal {L}^N_DF(\zeta )=\frac{1}{2}\sigma ^2\sum _{i,j\in \mathcal {N}(\zeta )}{\text {Tr}} \left( C_\epsilon (\xi (x_i-x_j))\nabla ^2_{x_ix_j}F(\zeta )\right) +\frac{1}{2}\sigma _0^2\sum _{i\in \mathcal {N}(\zeta )}\Delta _{x_i}F(\zeta ). \end{aligned}$$

### Remark 3

$\mathcal {L}_D^N$ accounts for the diffusion part of the dynamics, describing the diffusive movement of the particles between two consecutive annihilation events. The annihilation events happen at isolated times, which are sudden jumps in the particle configuration $\{\zeta _t^N\}_{t\ge 0}$, and are described by the jump part of the generator $\mathcal {L}_J^N$. While the form of $\mathcal {L}_D^N$ follows by an application of Itô’s formula to (16), the jump operator (19) can be written in terms of the jump rates $r_{ij}^N$ in a way that is familiar in interacting particle systems [16]. The total generator $\mathcal {L}^N$ is then the sum of the two. The Itô–Dynkin formula states that $t\mapsto F(\zeta _t^N)-F(\zeta _0^N)-\int _0^t \mathcal {L}^NF(\zeta _s^N)ds$ is a $\mathcal {G}_t$-martinagle. Naturally this martingale can be decomposed into two parts, one due to diffusion and the other one due to jump.

For the kernel function $\theta : \mathbb {R}^3\rightarrow \mathbb {R}_+$ that regulates the coalescence rate (3), it is enough to assume that it is Hölder continuous $\mathsf C^\alpha (\mathbb {R}^3)$ for some $\alpha \in (0,1)$, compactly supported in the unit ball $B(0,1)\subset \mathbb {R}^3$ with $\theta (x)=\theta (-x)$, $\int _{\mathbb {R}^3} \theta (x) dx =1$, and $\theta (0)=0$. Throughout, we denote its rescaled version by

$$ \theta ^\epsilon (x):=\epsilon ^{-3}\theta (\epsilon ^{-1}x), $$

which has compact support in a ball $B(0,\epsilon )$ of radius $\epsilon$. With this notation, the coalescence rate can be written as

20 $$\begin{aligned} r^N_{ij}=\frac{R_{0}}{N}\theta ^\epsilon (x_i-x_j). \end{aligned}$$

Denote the process of empirical measure of the *active* particles by

21 $$\begin{aligned} \mu ^N_t(dx):=\frac{1}{N}\sum _{i\in \mathcal {N}(\zeta _t^N)}\delta _{x_i^N(t)}(dx) \quad \in \mathcal {D}\left( [0,\infty ); \mathcal {M}_{+,1}(\mathbb {R}^3)\right) ,\quad t\ge 0, \end{aligned}$$

where $\mathcal {M}_{+,1}(\mathbb {R}^3)$ denotes the space of sub-probability measures over $\mathbb {R}^3$ endowed with the weak topology, and $\mathcal {D}\left( [0,\infty ); \mathcal {M}_{+,1}(\mathbb {R}^3)\right)$ denotes the Skorohod space of càdlàg processes taking values in $\mathcal {M}_{+,1}(\mathbb {R}^3)$ endowed with the Skorohod topology.

We introduce the limiting pde with unknown $f(t,x): [0,T]\times \mathbb {R}^3\rightarrow \mathbb {R}_+$:

22 $$\begin{aligned} {\left\{ \begin{array}{ll} \partial _tf(t,x)& =\frac{1}{2}\left( \sigma _0^2+\sigma ^2\right) \Delta f(t,x)-\overline{\mathcal {R}} f(t,x)^2,\\ f(0,x)& =f_0(x), \end{array}\right. } \end{aligned}$$

where $\overline{\mathcal {R}}\in \mathbb {R}_+$ is given by

23 $$\begin{aligned} \overline{\mathcal {R}}=\int _{\mathbb {R}^3}R_0\theta (x)\left( 1+u(x)\right) dx. \end{aligned}$$

and $u:\mathbb {R}^3\rightarrow \mathbb {R}$ solves the auxiliary pde

24 $$\begin{aligned} \sigma _0^2\Delta u(x)+\sigma ^{2}\nabla \cdot \big (\omega (\xi x)\nabla u(x)\big ) =R_{0} \theta (x)\big ( 1+u(x) \big ). \end{aligned}$$

We will comment later that (24) has a $\mathsf C^{2, \alpha }(\mathbb {R}^3)$ solution that satisfies $u(x)\in [-1,0]$ for all $x\in \mathbb {R}^3$, see (74) and Lemma 15, and is unique under the constraint that $|u(x)|\rightarrow 0$ as $|x|\rightarrow \infty$.

### Remark 4

An alternative expression for $\overline{\mathcal {R}}$ is

25 $$\begin{aligned} \overline{\mathcal {R}}:=\left( \sigma _0^2+\sigma ^2\right) \int _{\mathbb {R}^3}\Delta u(x)dx, \end{aligned}$$

and it is in this form that our proof will yield. It can be seen from integration by parts in (24); indeed since $\omega (x)=I_3-C (x)$ and *C* has compact support, we have

$$\begin{aligned} \big (\sigma _0^2+\sigma ^2\big )\int _{\mathbb {R}^3}\Delta u(x)dx&= \sigma ^2\int _{\mathbb {R}^3}\nabla \cdot \Big (C (\xi x)\nabla u(x)\Big )dx+\int _{\mathbb {R}^3}R_0\theta (x)(1+u(x))dx\\&=\int _{\mathbb {R}^3}R_0\theta (x)\big (1+u(x)\big )dx. \end{aligned}$$

Solutions to (22) are understood in the following weak sense.

### Definition 5

Assume $f_0\in L^1\cap L^\infty (\mathbb {R}^3;\mathbb {R}_+)$. We say that $f\in L^\infty \left( [0,T]; L^1(\mathbb {R}^3;\mathbb {R}_+)\right)$ is a weak solution to (22) if

$$\begin{aligned} \int _{\mathbb {R}^3} f(t,x)\phi (x)dx&=\int _{\mathbb {R}^3} f_0(x)\phi (x)dx + \frac{1}{2}\big (\sigma _0^2+\sigma ^2\big )\int _0^t\int _{\mathbb {R}^3} f(s,x)\Delta \phi (x)dxds\\&\quad -\overline{\mathcal {R}}\int _0^t\int _{\mathbb {R}^3} \phi (x)f(s,x)^2dxds \end{aligned}$$

holds for any $t\in [0,T]$ and all test function $\phi \in C^2_c(\mathbb {R}^3)$.

One can show that such weak solutions are unique, for a proof in a very similar context see [9, Section 3]. The main result of this article is as follows.

### Theorem 6

Assume that the initial density $f_0$ satisfies Condition 2 and the scaling relation (2) holds. Then for every $T<\infty$, the empirical measure $(\mu ^N_t(dx))_{t\in [0,T]}$ (21) of the particle system converges in probability as $N\rightarrow \infty$, in the space $\mathcal {D}\big ([0,T]; \mathcal {M}_{+,1}(\mathbb {R}^3)\big )$, to a limit $\{\overline{\mu }_t(dx)\}_{t\in [0,T]}$. The limit is deterministic, absolutely continuous with respect to Lebesgue measure for every *t*, i.e. $\overline{\mu }_t(dx)=f(t,x)dx$, where the density *f*(*t*, *x*) is the unique weak solution of the pde (22), in the sense of Definition 5.

This result, in particular the form of the average coalescence rate $\overline{\mathcal {R}}$, is a variant of [13]. Compared to that work, we have simplified the model by focusing only on particles of the same mass, but we have introduced a random environment to model the turbulent fluid. In our previous model [10] in a random environment, we have chosen the dense particle scaling $\epsilon ^3 N=1$ which, in the framework studied here, would lead to $\overline{\mathcal {R}}=R_0$.

### Sketch of Proof

Since the proof of our main result is somewhat technical, we will first provide a general sketch in this section.

Our goal is to show that the empirical measure process, $(\mu _t^N(dx))_{t\in [0,T]}$, which is a measure-valued stochastic process, converges to the unique weak solution of the pde (22). Granted that we have relative compactness of this sequence, we need to show that any subsequential limit is a weak solution of the pde. Since weak formulation of the pde is in terms of integration against test functions $\phi$, it is natural and standard to look at the real-valued stochastic process

$$ \langle \mu _t^N, \phi \rangle $$

for any fixed $\phi$, and show that asymptotically it satisfies the weak formulation of the pde with test function $\phi$.

At the level of fixed *N*, by applying Itô–Dynkin formula to $\langle \mu _t^N, \phi \rangle$, we can readily get that it satisfies a so-called semimartingale equation. Such equation is still random, with fluctuation terms present, and it only partially looks like the limit pde. In Sect. 3, we will show that the fluctuation terms (they are martingales) vanish in the limit $N\rightarrow \infty$ in a probabilistic sense, so that the limit is deterministic and not a stochastic pde. We will also see that all the other terms, but one of them (31), are in the form of the empirical measure $\mu _t^N$ integrated against certain test function. These are terms linear in the empirical measure, and will converge to corresponding linear terms of the pde, just because the empirical measure converges weakly (along subsequences).

There is however one term (31) which is not linear in the empirical measure. The desired limit for this term is quadratic in the pde solution, with a particular multiplicative constant $\overline{\mathcal {R}}$, but it is not at all clear if and how this stochastic term will converge. This difficulty is common in the theory of interacting particle systems, see [16], namely the main work is always concentrated on proving that the nonlinear terms converge. For particle systems evolving on lattices, there are methods bearing the name of “replacement lemma” that deal with such issues, but that requires detailed knowledge of invariant measures of the particle system. Lacking such knowledge in our setting of interacting diffusions in the continuum, we instead rely on a very special technique due to Hammond–Rezakhanlou [13]. We call it Itô–Tanaka trick, with roots in Tanaka’s formula in stochastic calculus; this is developed in Sects. 4–6 of our paper. The key statement to prove here is Proposition 11, which directly dictates the form of the multiplicative constant $\overline{\mathcal {R}}$ in the limit pde, see the argument following it, (43). However, we do not have enough intuitive or physical understanding of what this proposition means. Vaguely, we may say that it is also a sort of replacement lemma. From Proposition 11, it is relatively easy to deduce the desired convergence of the nonlinear stochastic term, because it allows to disentangle $\epsilon$ and *N* by introducing the auxiliary variable *z*.

The remaining effort is thus concentrated on proving Proposition 11. Let us look back at the nonlinear stochastic term (31), which is in the form of an averaged double-sum involving $\theta ^\epsilon (x_i^N(t)-x_j^N(t))$. Recall that $\epsilon \rightarrow 0$ very fast as $N\rightarrow \infty$, and $\theta ^\epsilon$ is approaching the Dirac delta. Furthermore, the argument of $\theta ^\epsilon$ is the two-point motion that is responsible for coalesence. Due to the singular nature of $\theta ^\epsilon$, this expression is highly unstable. The idea of Itô–Tanaka trick is that instead of working with $\theta ^\epsilon$, we can equivalently work with a much more regular function. Note that regularity here should be understood as not for fixed $\epsilon$, but as $\epsilon \rightarrow 0$. In Sect. 4, we construct an auxiliary function $u^\epsilon$, solution of a carefully-chosen, uniformly elliptic, second-order, divergence form pde (35) (the “cell equation”), whose right-hand side contains $\theta ^\epsilon$. As a result, $u^\epsilon$ is “two derivatives” more regular than $\theta ^\epsilon$ and not a singular function at all. By rescaling the cell equation, we can obtain an $\epsilon$-independent elliptic equation (36) that can be solved using Green’s function. Here, the capacity scaling relation (2) between $\epsilon$ and *N* plays an important role in correctly rescaling the cell equation. Then, in Sect. 5 we consider a new averaged double-sum process (48), similar to the nonlinear term (31) we started with, but now involving the more regular function $u^\epsilon$. By applying the Itô–Dynkin formula to this new process, and performing substitution using the cell equation, we obtain an identity in which the pre-limit of Proposition 11 appears, together with many other (presumably minor) terms. Now, to show Proposition 11, it is enough to control all these minor terms, since we have an identity in this manouvre. More precisely, we need to prove that all the minor terms are negligible in suitable probabilistic sense. Since they all involve $u^\epsilon$ and $\nabla u^\epsilon$, for which we have enough control through Green’s functions, we can prove their negligibility which is the content of Sect. 6.

The rest of the article is organized as follows. In Sect. 3 we write the equation satisfied by the empirical measure of the particle system, and identify the nonlinear term whose convergence is the main issue. In Sects. 4 and 5 we introduce the cell equation and the Itô–Tanaka trick, main ingredients in the proof of Theorem 6. In Sect. 6 we show the negligibility of all the minor terms coming out of Itô–Tanaka trick. We omit the proof of tightness of the particle system, the existence of limit density and the uniqueness of the limit pde, as they are similar to arguments detailed in our previous works [9, 10, 14]. In Sect. 7 we tie our main theorem with certain facts in potential theory, so as to deduce the dependence of coalesence efficiency on $\xi$.

## Equation Satisfied by the Empirical Measure

Now, we are ready to derive a semimartingale equation for the empirical measure process (21). Fixing $T<\infty$ and any $\phi \in C^2_c(\mathbb {R}^3)$, we apply the Itô–Dynkin formula (with infinitesimal generator given in (17)) to the functional of configurations

$$\begin{aligned} F_1(\zeta _t^N):=\langle \mu _t^N, \phi \rangle = \frac{1}{N}\sum _{i\in \mathcal {N}(\zeta _t^N)}\phi \left( x_i^N(t)\right) , \quad t\ge 0, \end{aligned}$$

where we use the shorthand $\langle \mu , f\rangle :=\int f d\mu$ for pairings between a function and a measure, and we obtain that

26 $$\begin{aligned}&\big \langle \mu _T^N, \phi \big \rangle - \big \langle \mu _0^N, \phi \big \rangle \end{aligned}$$

27 $$\begin{aligned}&\quad =\int _0^T\frac{1}{N}\frac{\sigma ^2}{2}\sum _{i\in \mathcal {N}\big (\zeta _t^N\big )}\nabla \cdot \big (C_\epsilon \big (\xi \big (x_i^N(t)-x_i^N(t)\big )\big )\nabla \phi \big (x_i^N(t)\big )\big )dt \end{aligned}$$

28 $$\begin{aligned}&\qquad +\int _0^T\frac{1}{N}\frac{\sigma ^2_0}{2}\sum _{i\in \mathcal {N}\big (\zeta _t^N\big )}\Delta \phi \big (x_i^N(t)\big )dt \end{aligned}$$

29 $$\begin{aligned}&\qquad +\int _0^T\frac{1}{N}\sigma \sum _{i\in \mathcal {N}\big (\zeta _t^N\big )}\nabla \phi \left( x_i^N(t)\right) \cdot \sum _{k\in \mathbb {N}}\lambda _ke^\epsilon _k\big (\xi x_i^N(t)\big )dW_k(t) \end{aligned}$$

30 $$\begin{aligned}&\qquad +\int _0^T\frac{1}{N}\sigma _0\sum _{i\in \mathcal {N}\big (\zeta _t^N\big )}\nabla \phi \big (x_i^N(t)\big )\cdot dB_i(t) \end{aligned}$$

31 $$\begin{aligned}&\qquad -\int _0^T\frac{1}{2}\frac{R_0}{N^2}\sum _{i\in \mathcal {N}\big (\zeta _t^N\big )}\sum _{j\in \mathcal {N}\big (\zeta _t^N\big ): j\ne i}\theta ^\epsilon \big (x_i^N(t)-x_j^N(t)\big )\big [\phi \big (x_i^N(t)\big )+\phi \big (x^N_j(t)\big )\big ]dt \nonumber \\&\qquad +M_J^N(T), \end{aligned}$$

where, while (29) and (30) are martinagles associated with diffusion dynamics, $M_J^N(t)$ is a martingale associated with jumps, that we do not write out explicitly. Since $C_\epsilon (0)=I_3$, we have

$$\begin{aligned} \frac{1}{N}\frac{\sigma ^2}{2}\sum _{i\in \mathcal {N}\big (\zeta _t^N\big )}\nabla \cdot \big (C_\epsilon \big (\xi \big (x_i^N(t)-x_i^N(t)\big )\big )\nabla \phi \big (x_i^N(t)\big )\big )=\frac{\sigma ^2}{2}\big \langle \mu _t^N, \Delta \phi \big \rangle . \end{aligned}$$

Also, since we assumed that $\theta$ is symmetric, we have

$$\begin{aligned}&\frac{1}{2}\frac{R_0}{N^2}\sum _{i\in \mathcal {N}\big (\zeta _t^N\big )}\sum _{j\in \mathcal {N}\big (\zeta _t^N\big ): j\ne i}\theta ^\epsilon \big (x_i^N(t)-x_j^N(t)\big )\big [\phi \big (x_i^N(t)\big )+\phi \big (x^N_j(t)\big )\big ]\\&=\frac{R_0}{N^2}\sum _{i\in \mathcal {N}\big (\zeta _t^N\big )}\sum _{j\in \mathcal {N}\big (\zeta _t^N\big ): j\ne i}\theta ^\epsilon \big (x_i^N(t)-x_j^N(t)\big )\phi \big (x_i^N(t)\big ). \end{aligned}$$

It turns out that the convergence of the linear terms (26)–(28) is not an issue (see e.g. [10, 14]), and we will prove in Lemmas 9 and 10 that the martingale terms vanish, as $N\rightarrow \infty$. The only difficulty is the convergence of the nonlinear term (31).

We introduce a tool that will be useful also for later sections. Consider on the same filtered probability space $\big (\Omega , \mathcal {F}, \{\mathcal {G}_t\}_{t\ge 0}, \mathbb {P}\big )$ an anxiliary “free” particle system $y_i^\epsilon (t)$, $i\in \mathbb {N}$ which start at $t=0$ with the same initial condition as the true system $y_i^\epsilon (0)=x_i(0)$, perform the same sde (16) driven by the same collections of Brownian motions $\{W_k(t)\}_{k\in \mathbb {N}}$ and $\{B_i(t)\}_{i=1}^\infty$, but here the particles do not interact/annihilate, i.e.

32 $$\begin{aligned} dy_i^\epsilon (t)=\sigma \sum _{k\in \mathbb {N}}\lambda _k e_{k}^{\epsilon }\left( \xi y_i^\epsilon (t)\right) dW_k(t)+\sigma _0\, dB_i(t), \quad t\ge 0, \;i=1,2,... \end{aligned}$$

with infinite life time. Note that $y_i^\epsilon$ still depends on $\epsilon$.

Since the initial conditions and dynamics of the true and auxiliary systems agree, there is an obvious coupling between them under which whenever $x_i^N(t)$ is alive at time *t*, its trajectory on the time interval [0, *t*] coincides with that of $y_i^\epsilon (t)$, for every $i=1,2,..,N$. By construction, the auxiliary system is exchangeable, meaning that the law of $\big (y_{i_1}^\epsilon (t), y_{i_2}^\epsilon (t),..., y_{i_L}^\epsilon (t)\big )_{t\ge 0}$ is unchanged under any permutation of the indices $(i_1,i_2,...,i_L)$ and any finite $L\in \mathbb {N}$. Now consider a pair $\big (y_1^\epsilon (t), y^\epsilon _2(t)\big )$ and it is known classically that it has a joint density $f_t^{12,\epsilon }(y_1,y_2)$ that evolves by the forward Kolmogorov equation (or Fokker–Planck equation)

$$\begin{aligned} {\left\{ \begin{array}{ll} \partial _tf_t^{12,\epsilon }(\underline{y})& =\mathcal {L}_\epsilon ^*f_t^{12,\epsilon }(\underline{y}), \\ f_0^{12,\epsilon }(\underline{y})& =f_0(y_1)f_0(y_2), \quad \quad \underline{y}:=(y_1,y_2)\in (\mathbb {R}^3)^2, \end{array}\right. } \end{aligned}$$

where $\mathcal {L}^*_\epsilon$ is a uniformly negative divergence-form operator that acts on functions $g:(\mathbb {R}^3)^2\rightarrow \mathbb {R}$ by

$$\begin{aligned} \mathcal {L}_\epsilon ^*g\big (\underline{y}\big )=\nabla _{\underline{y}}\cdot \big (A_\epsilon \big (\underline{y}\big )\nabla _{\underline{y}}\big ) \end{aligned}$$

where

$$\begin{aligned} \nabla _{\underline{y}}&:=\big (\nabla _{y_1}, \nabla _{y_2}\big ), \\ A_\epsilon (\underline{y})&:=\frac{1}{2}\sigma _0^2I_6+\frac{1}{2}\sigma ^2\begin{bmatrix} I_3 & C_\epsilon (\xi (y_2-y_1))\\ C_\epsilon (\xi (y_1-y_2)) &  I_3\end{bmatrix}\in \mathbb {M}_6. \end{aligned}$$

Although $\mathcal {L}_\epsilon ^*$ depends on $\epsilon$, its matrix coefficient $A_\epsilon$ are measurable and bounded uniformly in $\epsilon$. Indeed, $C_\epsilon (x)=C(x/\epsilon )$ and *C*(*x*) is assumed smooth and compactly supported. By Aronson’s estimate [3, Theorem 1] for fundamental solutions of uniformly parabolic second-order divergence-form operator with measurable bounded coefficients, there exist some finite constants $\mathsf K_i=\mathsf K_i \left( \sigma _0, \sigma , \Vert C\Vert _{L^\infty }\right)$ (depending only on the bounds on the coefficients, hence independent of $\epsilon$), $i=1,2$, such that

$$\begin{aligned} q^{\epsilon }\big (t; \underline{y}_0, \underline{y}\big )\le \frac{\mathsf K_1}{(2\pi \mathsf K_2t)^3}e^{-\frac{|\underline{y}-\underline{y}_0|^2}{2\mathsf K_2t}}, \end{aligned}$$

where $q^{\epsilon }\big (t; \underline{y}_0, \underline{y}\big )$ denotes the fundamental solution of $\partial _t-\mathcal {L}_\epsilon ^*$, and $\Vert C\Vert _{L^\infty }:=\max _{\alpha ,\beta =1}^3 \Vert C^{\alpha \beta }\Vert _{L^\infty (\mathbb {R}^3)}$.

This allows to derive exponential tail bounds on $f_t^{12,\epsilon }$. Indeed, let $Y_t$ denote a Gaussian random vector on $(\mathbb {R}^3)^2$ with density $\frac{1}{(2\pi \mathsf K_2t)^3}e^{-\frac{|\underline{y}|^2}{2\mathsf K_2t}}$, then

$$\begin{aligned} f_t^{12,\epsilon }\big (\underline{y}\big )&=\int _{(\mathbb {R}^3)^2} f_0^{12,\epsilon }\big (\underline{y}_0\big )q^{\epsilon }\big (t; \underline{y}_0, \underline{y}\big )d\underline{y}_0\\&\le \int _{(\mathbb {R}^3)^2} f_0^{12,\epsilon }\big (\underline{y}_0\big )\frac{\mathsf K_1}{(2\pi \mathsf K_2t)^3}e^{-\frac{|\underline{y}-\underline{y}_0|^2}{2\mathsf K_2t}} d\underline{y}_0\\&=\mathsf {K_1}\mathbb {E}\left[ f_0^{12,\epsilon }(\underline{y}-Y_t)\right] , \end{aligned}$$

where $\mathbb {E}$ acts on the random variable $Y_t$. Since $f_0^{12,\epsilon }(\underline{y})=f_0(y_1)f_0(y_2)$ which is uniformly bounded by $\Gamma ^2$ and compactly supported in $B(0, \sqrt{2}L_0)$ by Condition 2, we have

$$\begin{aligned} f_t^{12,\epsilon }\big (\underline{y}\big )\le \mathsf {K_1}\mathbb {E}\left[ f_0^{12,\epsilon }(\underline{y}-Y_t)\right]&\le \mathsf {K_1}\Gamma ^2\mathbb {P}\left( |\underline{y}-Y_t|\le \sqrt{2}L_0\right) \\&\le \mathsf {K_1} \Gamma ^2\mathbb {P}\left( |Y_t|\ge |\underline{y}|-\sqrt{2}L_0\right) \\&\le 2\mathsf {K_1} \Gamma ^2 e^{-\frac{\left( |\underline{y}|-\sqrt{2}L_0\right) _+^2}{4d\mathsf {K_2} t}}\le \textsf{K}'e^{-|\underline{y}|^2/\textsf{K}'}, \end{aligned}$$

for any $t\in [0,T]$ and some finite constant $\textsf{K}'$ that depends only on $\mathsf {K_1}, \mathsf {K_2}, \Gamma , T, L_0$, where we used the tail bound for a Gaussian vector.

A similar argument can also show that the joint density $f_t^{12...\ell ,\epsilon }(y_1,...,y_\ell )$ of an $\ell$-tuple of free particles $(y_1^\epsilon (t),..., y_\ell ^\epsilon (t))$, for any fixed $\ell \in \mathbb {N}$, also satisfies an exponential decay, uniformly for $t\in [0,T]$ and $\epsilon$. We will only use $\ell =1,2,3,4$ in the sequel. That is, we have proven that

### Proposition 7

There exists a finite constant $C_0=C_0\left( \sigma _0, \sigma , \Vert C\Vert _{L^\infty }, \Gamma , T, L_0, \ell \right)$ such that for any $t\in [0,T]$ and $\epsilon \in (0,1)$, we have

33 $$\begin{aligned} f_t^{1...\ell ,\epsilon }(y_1,y_2,...,y_\ell )&\le C_0e^{-\frac{|y_1|^2+...+|y_\ell |^2}{C_0}}, \end{aligned}$$

for $$\ell =1,2,3,4$$.

We proceed to show next that the two martingale terms associated with diffusion vanish in $L^2(\mathbb {P})$.

### Lemma 8

$$\begin{aligned} \mathbb {E}\left| \int _0^T\frac{1}{N}\sum _{i\in \mathcal {N}(\zeta _t^N)}\nabla \phi \left( x_i^N(t)\right) \cdot \sum _{k\in \mathbb {N}}\lambda _ke^\epsilon _k(x_i^N(t))dW_k(t)\right| ^2=O\left( N^{-1}\right) +O\left( \epsilon ^3\right) . \end{aligned}$$

### Proof

By Itô isometry and the independence between $W_k(t)$, $k\in \mathbb {N}$,

$$\begin{aligned}&\mathbb {E}\left| \int _0^T\frac{1}{N}\sum _{i\in \mathcal {N}\big (\zeta _t^N\big )}\nabla \phi \left( x_i^N(t)\right) \cdot \sum _{k\in \mathbb {N}}\lambda _ke^\epsilon _k\big (\xi x_i^N(t)\big )dW_k(t)\right| ^2\\&=\mathbb {E}\int _0^T\frac{1}{N^2}\sum _{k\in \mathbb {N}}\left| \sum _{i\in \mathcal {N}\big (\zeta _t^N\big )}\nabla \phi \left( x_i^N(t)\right) \cdot \lambda _ke^\epsilon _k\big (\xi x_i^N(t)\big )\right| ^2dt\\&=\mathbb {E}\int _0^T\frac{1}{N^2}\sum _{k\in \mathbb {N}}\sum _{i,j\in \mathcal {N}\big (\zeta _t^N\big )}\nabla \phi \big (x_i^N(t)\big )^T \lambda _ke^\epsilon _k\big (\xi x_i^N(t)\big )\otimes \lambda _ke^\epsilon _k\big (\xi x_j^N(t)\big )\nabla \phi \big (x_j^N(t)\big )dt\\&=\mathbb {E}\int _0^T\frac{1}{N^2}\sum _{i,j\in \mathcal {N}\big (\zeta _t^N\big )}\nabla \phi \big (x_i^N(t)\big )^TC_\epsilon \big (\xi \big (x_i^N(t)-x_j^N(t)\big )\big )\nabla \phi \big (x_j^N(t)\big )dt\\&\le \frac{T}{N}\Vert \phi \Vert _{C^1}^2+\mathbb {E}\int _0^T\frac{9}{N^2}\sum _{i\ne j\in \mathcal {N}\big (\zeta _t^N\big )}\Vert \phi \Vert _{C^1}^2\Big \Vert C_\epsilon \big (\xi \big (x_i^N(t)-x_j^N(t)\big )\big )\Big \Vert _\infty dt, \end{aligned}$$

where for a $3\times 3$ matrix *A* we denote

34 $$\begin{aligned} \Vert A\Vert _\infty :=\max _{\alpha ,\beta =1}^3|A^{\alpha \beta }|. \end{aligned}$$

Recall the auxiliary free particle system (32) just introduced, which is exchangeable in law and coupled to $x_i^N$ for $i\in \mathcal {N}(\zeta _t^N)$ in a natural way, hence we have the bound

$$\begin{aligned}&\mathbb {E}\int _0^T\frac{1}{N^2}\sum _{i,j\in \mathcal {N}\big (\zeta _t^N\big )}\left\| C_\epsilon \big (\xi \big (x_i^N(t)-x_j^N(t)\big )\big )\right\| _\infty dt\\&\le \mathbb {E}\int _0^T\frac{1}{N^2}\sum _{i\ne j=1}^N\left\| C_\epsilon \big (\xi \big (y_i^\epsilon (t)-y_j^\epsilon (t)\big )\big )\right\| _\infty dt\\&\le \mathbb {E}\int _0^T\left\| C_\epsilon \left( \xi \big (y_1^\epsilon (t)- y_2^\epsilon (t)\big )\right) \right\| _\infty dt. \end{aligned}$$

By the tail bound on the pair density (33),

$$\begin{aligned}&\le T\iint _{(\mathbb {R}^3)^2}\big \Vert C\left( \epsilon ^{-1}\xi (y_1-y_2)\right) \big \Vert _\infty C_0e^{-\frac{|y_1|^2+|y_2|^2}{C_0}} dy_1dy_2dt\\&=C_0T\int _{\mathbb {R}^3}\big \Vert C\left( \epsilon ^{-1}\xi y_1\right) \big \Vert _\infty dy_1\int _{\mathbb {R}^3}e^{-\frac{|y_2|^2}{C_0}} dy_2\\&\lesssim \epsilon ^3 \int _{\mathbb {R}^3} \Vert C\left( \xi y\right) \Vert _\infty dy=O(\epsilon ^3), \end{aligned}$$

where the factor $\epsilon ^3$ comes from change of variables, and the last integral is finite since every component of *C* is smooth with compact support (hence bounded). This completes the proof. $\square$

### Lemma 9

$$\begin{aligned} \mathbb {E}\left| \int _0^T\frac{1}{N}\sigma _0\sum _{i\in \mathcal {N}\big (\zeta _t^N\big )}\nabla \phi \big (x_i^N(t)\big )\cdot dB_i(t)\right| ^2=O\left( N^{-1}\right) . \end{aligned}$$

### Proof

By Itô isometry, we have

$$\begin{aligned}&\mathbb {E}\left| \int _0^T\frac{1}{N}\sigma _0\sum _{i\in \mathcal {N}\big (\zeta _t^N\big )}\nabla \phi \big (x_i^N(t)\big )\cdot dB_i(t)\right| ^2\\&=\mathbb {E}\sum _{i\in \mathcal {N}\big (\zeta _t^N\big )}\int _0^T\frac{1}{N^2}\sigma _0^2 \big |\nabla \phi \big (x_i^N(t)\big )\big |^2dt\\&\le T\sigma _0^2N^{-2}\Vert \phi \Vert _{C^1}^2 \mathbb {E}\big |\mathcal {N}(\eta (t))\big |\le T\sigma _0^2N^{-1}\Vert \phi \Vert _{C^1}^2, \end{aligned}$$

since $$|\mathcal {N}(\eta (t))|\le |\mathcal {N}(\eta (0))|=N$$. $$\square $$

We show next that the martinagle associated with jumps also vanishes in $L^2(\mathbb {P})$.

### Lemma 10

$$\mathbb {E}\big |M_J^N(T)\big |^2=O\left( N^{-1}\right) $$.

### Proof

By the carré-du-champ formula cf. [6, Proposition 8.7], we have

$$\begin{aligned} \mathbb {E}\big |M_J^N(T)\big |^2&\le \mathbb {E}\int _0^T\frac{R_0}{2N}\sum _{i\in \mathcal {N}\big (\zeta _t^N\big )}\sum _{j\in \mathcal {N}\big (\zeta _t^N\big ):j\ne i}\theta ^\epsilon \big (x_i^N(t)\\&\quad -x_j^N(t)\big )\frac{1}{N^2}\big [\phi \big (x_i^N(t)\big )+\phi \big (x_j^N(t)\big )\big ]^2dt \\&\le \mathbb {E}\int _0^T\frac{2R_0\Vert \phi \Vert _{L^\infty }^2}{N^3}\sum _{i\in \mathcal {N}\big (\zeta _t^N\big )}\sum _{j\in \mathcal {N}\big (\zeta _t^N\big ):j\ne i}\theta ^\epsilon \big (x_i^N(t)-x_j^N(t)\big )dt. \end{aligned}$$

On the other hand, we consider the functional $F_2(\zeta _t^N)=|\mathcal {N}(\zeta _t^N)|$, apply the generator (17) to it, and we get

$$\begin{aligned} \mathbb {E}\big |\mathcal {N}\big (\eta _T^N\big )\big |-\mathbb {E}\big |\mathcal {N}\big (\eta _0^N\big )\big |= -\mathbb {E}\, 2\int _0^T\frac{R_0}{2N}\sum _{i\in \mathcal {N}\big (\zeta _t^N\big )}\sum _{j\in \mathcal {N}\big (\zeta _t^N\big ):j\ne i}\theta ^\epsilon \big (x_i^N(t)-x_j^N(t)\big )dt. \end{aligned}$$

Indeed, the diffusion part of the generator does not affect $F_2(\zeta _t^N)$, only the jump part does. It yields that

$$\begin{aligned} \mathbb {E}\int _0^T\frac{R_0}{N}\sum _{i\in \mathcal {N}\big (\zeta _t^N\big )}\sum _{j\in \mathcal {N}\big (\zeta _t^N\big ):j\ne i}\theta ^\epsilon \big (x_i^N(t)-x_j^N(t)\big )dt\le \mathbb {E}|\mathcal {N}(\eta _0)|=N. \end{aligned}$$

Therefore we have $\mathbb {E}|M_J^N(T)|^2\le 2\Vert \phi \Vert _{L^\infty }^2N^{-1}$. $\square$

## The Cell Problem and the Nonlinear Term

The remaining task is to prove the convergence of the nonlinear term (31). We aim to establish a form of local equilibrium as in Proposition 11 below. For this purpose, we introduce for every fixed $\epsilon \in (0,1)$ an auxiliary equation $u^{\epsilon }(x):\mathbb {R}^3\rightarrow \mathbb {R}$ (so-called cell problem in the terminology of homogenization):

35 $$\begin{aligned} \sigma _0^2\Delta u^\epsilon (x)+\sigma ^{2}\nabla \cdot \Big (\omega \Big (\frac{\xi x}{\epsilon }\Big ) \nabla u^{\epsilon }\left( x\right) \Big ) =R_{0} \theta ^\epsilon \left( x\right) \Big ( 1+\frac{u^{\epsilon }\left( x\right) }{N} \Big ) , \end{aligned}$$

which is a rescaling of an $\epsilon$-independent equation (36). Let $u(x):\mathbb {R}^3\rightarrow \mathbb {R}$ be a particular $\mathsf C^{2, \alpha }(\mathbb {R}^3)$-solution given in (74), of

36 $$\begin{aligned} \sigma _0^2\Delta u(x)+\sigma ^{2}\nabla \cdot \big (\omega (\xi x)\nabla u(x)\big ) =R_{0} \theta (x)\big ( 1+u(x) \big ), \end{aligned}$$

then it can be readily checked, using $N=\epsilon ^{-1}$, that the rescaled function

37 $$\begin{aligned} u^\epsilon (x)=\epsilon ^{-1}u\left( \frac{x}{\epsilon }\right) \end{aligned}$$

solves (35). This is the analogous cell equation as used in [13, Eq. (1.7)].

The following proposition will be proved in Sect. 5 using the Itô–Tanaka trick. Note that we have added an extra test function $\psi$, with respect to (31), to have more localization. It is actually without loss of generality. Indeed, $\theta ^\epsilon (x)$ is compactly supported in $B(0,\epsilon )$ and from (35) it can be seen (45) that $\Delta u^\epsilon (x)$ is compactly supported in $B(0,\epsilon (1\vee \xi ^{-1}))$, and we can always assume that $|z|\le 1/2$, which implies that each summand

$$ R_0\theta ^\epsilon \big (x_i^N(t)-x_j^N(t)\big )-\left( \sigma _0^2+\sigma ^2\right) \Delta u^\epsilon \big (x_i^N(t)-x_j^N(t)+z\big ) $$

is zero unless $|x_i^N(t)-x_j^N(t)|\le 1/2+\epsilon (1\vee \xi )$. Since $x_i^N(t)\in {\text {supp}}(\phi )$, this forces $x_j^N(t)$ to be inside some other compact set $\mathbb {K}=\mathbb {K}(\phi ,\xi )\subset \mathbb {R}^3$. Hence we can choose a compactly supported test function $\psi$ that is identically 1 on $\mathbb {K}$ and decays smoothly to 0 outside. Such a $\psi (x_j^N(t))$ does not alter (38).

### Proposition 11

Let $u^{\epsilon }(x)\in \mathsf C^{2, \alpha }(\mathbb {R}^3)$ be given by (37) and $\phi , \psi \in C_c^2(\mathbb {R}^3)$ be two test functions, then we have

38 $$\begin{aligned}&\lim _{|z|\rightarrow 0}\limsup _{N\rightarrow \infty }\nonumber \\&\quad \mathbb {E}\Biggl |\int _0^T\frac{1}{N^2}\sum _{i, j\in \mathcal {N}(\zeta _t^N): j\ne i}\Big [R_0\theta ^\epsilon \big (x_i^N(t)-x_j^N(t)\big )-\left( \sigma _0^2+\sigma ^2\right) \Delta u^\epsilon \big (x_i^N(t)-x_j^N(t)+z\big )\Big ]\nonumber \\&\quad \phi (x_i^N(t))\psi (x_j^N(t))dt\Biggr |=0. \end{aligned}$$

We proceed directly to showing that Proposition 11 yields the convergence of the nonlinear term, as in [13, pp. 42–43]. Firstly, we note that we can add the terms with indices $i=j$ into the double summation of (38), without changing its conclusion. Indeed, there are at most *N* diagonal terms. With $\theta ^\epsilon (0)=0$, $|\Delta u^\epsilon (z)|\lesssim |z|^{-3}$ provided $|z|\ge 2\epsilon$ by (77), and a factor of $1/N^2$ in front, we see that as $\epsilon \rightarrow 0$ first (and *z* fixed), the whole contribution of

$$ \frac{1}{N^2}\sum _{i\in \mathcal {N}(\zeta _t^N)}\Big |R_0\theta ^\epsilon \big (x_i^N(t)-x_i^N(t)\big )-\left( \sigma _0^2+\sigma ^2\right) \Delta u^\epsilon \big (x_i^N(t)-x_i^N(t)+z\big )\Big |\lesssim N^{-1}|z|^{-3} \rightarrow 0. $$

Hence, we can start our subsequent argument with a version of (38) with full double summation:

39 $$\begin{aligned}&\lim _{|z|\rightarrow 0}\limsup _{N\rightarrow \infty }\nonumber \\&\quad \mathbb {E}\Biggl |\int _0^T\frac{1}{N^2}\sum _{i,j \in \mathcal {N}(\zeta _t^N)}\Big [R_0\theta ^\epsilon \big (x_i^N(t)-x_j^N(t)\big )-\left( \sigma _0^2+\sigma ^2\right) \Delta u^\epsilon \big (x_i^N(t)-x_j^N(t)+z\big )\Big ]\nonumber \\&\quad \phi (x_i^N(t))\psi (x_j^N(t))dt\Biggr |=0. \end{aligned}$$

Denote by $\zeta :\mathbb {R}^3\rightarrow \mathbb {R}_+$ a fixed smooth probability density function with compact support in *B*(0, 1) and denote $\zeta ^\delta (x):=\delta ^{-3}\zeta (x/\delta )$ for $\delta >0$. Then, (39) yields that

40 $$\begin{aligned}&\int _0^TR_0\left\langle \theta ^\epsilon (x_1-x_2)\phi (x_1)\psi (x_2), \mu _t^N(dx_1)\mu _t^N(x_2)\right\rangle dt \nonumber \\&=\int _0^T\left( \sigma _0^2+\sigma ^2\right) \iint _{(\mathbb {R}^3)^2}\left\langle \Delta u^\epsilon (x_1-x_2-z_1+z_2)\phi (x_1)\psi (x_2), \mu _t^N(dx_1)\mu _t^N(dx_2)\right\rangle \nonumber \\&\quad \zeta ^\delta (z_1)\zeta ^\delta (z_2)dz_1dz_2 dt \nonumber \\&\quad \quad \quad \quad +{\text {Err}}(\delta ,\epsilon ), \end{aligned}$$

where ${\text {Err}}(\delta ,\epsilon )$ is a stochastic error that vanishes in the following limit:

41 $$\begin{aligned} \lim _{\delta \rightarrow 0}\limsup _{\epsilon \rightarrow 0}\mathbb {E}|{\text {Err}}(\delta ,\epsilon )|=0, \end{aligned}$$

and may change from line to line in the sequel. In (40), we have introduced two averaging on variable $z_1,z_2$ with compact supports in $B(0,\delta )$, hence in view of (39), the rate of convergence of the error term (41) is uniformly controlled by $\delta$.

Next, we shift the argument of $\phi (x_1)$ and $\psi (x_2)$ on the right-hand side of (40) to $\phi (x_1-z_1)$ and $\psi (x_2-z_2)$ respectively, causing an error of $O(\delta )$, since $z_1,z_2$ are in the compact support of the function $\zeta ^\delta$. Then, we perform a change of variable $w_1=x_1-z_1$, $w_2=x_2-z_2$, and the right-hand side of (40) (in the sequel written without the time integral, to ease notation) now becomes

42 $$\begin{aligned}&\left( \sigma _0^2+\sigma ^2\right) \iint _{(\mathbb {R}^3)^2}\Delta u^\epsilon (w_1-w_2)\phi (w_1)\psi (w_2)\nonumber \\&\quad \left\langle \zeta ^\delta (x_1-w_1)\zeta ^\delta (x_2-w_2) , \mu _t^N(dx_1)\mu _t^N(x_2)\right\rangle \nonumber \\&\quad dw_1dw_2+{\text {Err}}(\delta ,\epsilon ). \end{aligned}$$

Next, we shift the argument of $\zeta ^\delta (x_2-w_2)$ to $\zeta ^\delta (x_2-w_1)$ in (42), causing an error on the order of $|w_2-w_1|\delta ^{-4}$ by the mean-value theorem and $|\nabla \zeta ^\delta |\lesssim \delta ^{-4}$, and the argument of $\psi (w_2)$ to $\psi (w_1)$, causing an error on the order of $|w_2-w_1|$, and (42) is equal to

43 $$\begin{aligned}&=\left( \sigma _0^2+\sigma ^2\right) \iint _{(\mathbb {R}^3)^2} \Delta u^\epsilon (w_1-w_2)\phi (w_1)\psi (w_1)\nonumber \\&\quad \left\langle \zeta ^\delta (x_1-w_1)\zeta ^\delta (x_2-w_1), \mu _t^N(dx_1)\mu _t^N(x_2)\right\rangle dw_1dw_2\nonumber \\&\quad \quad \quad +{\text {Err}}(\delta ,\epsilon )+{\text {Err}}_1(\delta ,\epsilon ), \end{aligned}$$

with an additional error term ${\text {Err}}_1$ that we need to show is also negligible. Indeed, recalling that $\mu _t^N$ is a sub-probability, we firstly have

44 $$\begin{aligned}&|{\text {Err}}_1(\delta ,\epsilon )|\lesssim \left( \sigma _0^2+\sigma ^2\right) \iint _{(\mathbb {R}^3)^2}| \Delta u^\epsilon (w_1-w_2)||\phi (w_1)||\psi (w_2)||w_1-w_2|\delta ^{-7}dw_1dw_2 \nonumber \\&\quad \quad \quad + \left( \sigma _0^2+\sigma ^2\right) \iint _{(\mathbb {R}^3)^2} |\Delta u^\epsilon (w_1-w_2)||\phi (w_1)||w_1-w_2|dw_1dw_2. \end{aligned}$$

Then, note by the cell equation (35) and since $\omega (x)=I_3-\overline{C}(x)$, we have

45 $$\begin{aligned} \left( \sigma _0^2+\sigma ^2\right) \Delta u^\epsilon (x) = \sigma ^{2}\nabla \cdot \Big (C _\epsilon (\xi x) \nabla u^{\epsilon }\left( x\right) \Big ) +R_{0} \theta ^\epsilon \left( x\right) \Big ( 1+\frac{u^{\epsilon }\left( x\right) }{N} \Big ). \end{aligned}$$

Since $C _\epsilon$ and $\theta ^\epsilon$ are compactly supported in $B(0,\epsilon /\xi )$ and $B(0,\epsilon )$ respectively, we have $\Delta u^\epsilon$ compactly supported in $B(0,\epsilon (1\vee \xi ^{-1}))$. Hence, recalling that $\Delta u^\epsilon (x) = \epsilon ^{-3}\Delta u(x/\epsilon )$, (44) can be bounded by

$$\begin{aligned} |{\text {Err}}_1(\delta ,\epsilon )|&\lesssim \iint _{(\mathbb {R}^3)^2}\bigg |\epsilon ^{-3} \Delta u\bigg (\frac{w_1}{\epsilon }\bigg )\bigg ||\phi (w_1+w_2)||\psi (w_2)||w_1|\delta ^{-7}dw_1dw_2 \\&\lesssim \epsilon \iint _{(\mathbb {R}^3)^2}\bigg |\epsilon ^{-3} \Delta u\bigg (\frac{w_1}{\epsilon }\bigg )\bigg ||\psi (w_2)|\delta ^{-7}dw_1dw_2\\&\lesssim \epsilon \int _{\mathbb {R}^3}|\Delta u(w_1)|\delta ^{-7}dw_1=O(\epsilon \delta ^{-7}), \end{aligned}$$

where since $\Delta u\in \mathsf C^{\alpha }(\mathbb {R}^3)$ and compactly supported in $B(0, 1\vee \xi ^{-1})$, it is integrable. Thus, we see that

$$\lim _{\delta \rightarrow 0}\limsup _{\epsilon \rightarrow 0}|{\text {Err}}_1(\delta ,\epsilon )|=0.$$

Using the coupling with the auxiliary free system (32), see [10, Sections 5–6] or [14, Section 6] for arguments in similar contexts, it can be shown that the sequence of laws of $\{\mu _t^N\}_N$ is weakly compact, and any subsequential limit of these laws is concentrated on those measure-valued processes that are absolutely continuous with respect to Lebesgue measure for every $t\ge 0$, with a density that is uniformly bounded by the right-hand side of (33) with $\ell =1$ (i.e. by the 1-particle density of the free particle system). Let $\{\mu _t^{N_k}\}_k$ be such a converging subsequence, and let the limit density be called *f*(*t*, *x*). Then for fixed $\delta$ we have as $N_k\rightarrow \infty$ (and $\epsilon (N_k)\rightarrow 0$),

$$\begin{aligned}  &   \left\langle \zeta ^\delta (x_1-w_1)\zeta ^\delta (x_2-w_1), \mu _t^N(dx_1)\mu _t^N(x_2)\right\rangle \rightarrow \\  &   \quad \iint _{(\mathbb {R}^3)^2} \zeta ^\delta (x_1-w_1)\zeta ^\delta (x_2-w_1)f(t, x_1)f(t, x_2)dx_1dx_2. \end{aligned}$$

Recall the definition of $\overline{\mathcal {R}}$ (25). Now, taking $\epsilon \rightarrow 0$ and then $\delta \rightarrow 0$ in (43), the error terms vanish in $L^1(\mathbb {P})$ and the main integral tends first to

$$\begin{aligned} \overline{\mathcal {R}} \int _{\mathbb {R}^{d}} \phi (w_1)\psi (w_1)\iint _{(\mathbb {R}^3)^2} \zeta ^\delta (x_1-w_1)\zeta ^\delta (x_2-w_1)f(t, x_1)f(t, x_2)dx_1dx_2dw_1, \end{aligned}$$

and then to

46 $$\begin{aligned} \overline{\mathcal {R}} \int _{\mathbb {R}^3} \phi (w_1)\psi (w_1)f(t, w_1)f(t,w_1)dw_1, \end{aligned}$$

since $\zeta ^\delta$ approximates the delta-Dirac (here one needs the apriori fact that *f* is bounded uniformly by a non-random constant, hence one can apply the dominated convergence theorem). In (46) we have obtained the desired weak formulation (in space) of the nonlinearity of (22).

In Sect. 3 we already saw that in the identity satisfied by the empirical measure, the linear terms converge easily to the desired limiting linear terms (since they are in the form of a measure integrated against a test function) and the martingale terms vanish in $L^2(\mathbb {P})$; the only issue is the convergence of the nonlinear term (31). In this section, we saw that provided Proposition 11 is proved, we can also prove the convergence of the nonlinear term to the desired limiting nonlinear term, along every weakly converging subsequence of $\{\mu _t^N\}_{t\in [0,T]}$. Thus a quite standard weak convergence argument as in e.g. [9, pp. 636–637] or [14, pp. 26–27] establishes the subsequential convergence in distribution of $\{\mu _t^N\}_{t\in [0,T]}$ to a weak solution of (22). Since it can be proved (see e.g. [9, Section 3]) that weak solutions to the pde in the sense of Definition 5 is unique hence deterministic, we conclude that the whole sequence $\{\mu _t^N\}_{t\in [0,T]}$ converges in probability. This is the proof of Theorem 6 (provided Proposition 11 is shown).

## Itô–Tanaka Procedure

In order to prove the key Proposition 11, we need to introduce a particular functional on the particle configuration that contains the macroscopic variable *z*, and by applying the generator to such a functional, we wish to make appear the pre-limit in (38). It is clear only a posteriori that it should contain an auxiliary function $v^{\epsilon ,z}(x)$ (47) that is in the form of a difference of the function $u^\epsilon (x)$, shifted by *z*.

For every fixed $\epsilon \in (0,1)$ and $z\in \mathbb {R}^3$ with $|z|\le 1/2$, denote

47 $$\begin{aligned} v^{\epsilon ,z}(x):=u^{\epsilon }(x+z)-u^{\epsilon }(x), \end{aligned}$$

where $u^{\epsilon }(x)$ is a $\mathsf C^{2, \alpha }(\mathbb {R}^3)$-solution of the $\epsilon$-dependent cell problem (35), given in (37). In particular, it is regular enough to apply Itô’s formula to. Consider the following functional on configurations

48 $$\begin{aligned} F_3\big (\zeta _t^N\big ):=\frac{1}{N^2}\sum _{i\in \mathcal {N}\big (\zeta _t^N\big )}\sum _{j\in \mathcal {N}\big (\zeta _t^N\big ): j\ne i}v^{\epsilon ,z}\big (x_i^N(t)-x_j^N(t))\phi (x_i^N(t))\psi \big (x_j^N(t)\big ), \quad t\ge 0, \end{aligned}$$

and we proceed to apply the Itô–Dynkin formula (with infinitesimal generator given in (17)) to it. We note the following property of the function *C*(*x*):

49 $$\begin{aligned} \sum _{\alpha =1}^3\partial _\alpha C^{\alpha \beta }(x) =0, \quad {\text {for all }}\beta =1,2,3, \;\; x\in \mathbb {R}^3. \end{aligned}$$

Indeed, by (15) $C(x)=\sum _{k\in \mathbb {N}}\lambda _ke_k(x)\otimes \lambda _ke_k(0)$. Since $e_k(x)$ is divergence free, for every $k\in \mathbb {N}$, we have

$$\begin{aligned} \sum _{\alpha =1}^3\partial _\alpha C^{\alpha \beta }(x)&=\sum _{k\in \mathbb {N}}\lambda _k^2\sum _{\alpha =1}^3\partial _\alpha \left( (e_k)^\alpha (x)(e_k)^\beta (0)\right) \\&=\sum _{k\in \mathbb {N}}\lambda _k^2\sum _{\alpha =1}^3\left( \partial _\alpha (e_k)^\alpha (x)\right) (e_k)^\beta (0)\\&=\sum _{k\in \mathbb {N}}\lambda _k^2\mathrm{{div}}\left( e_k\right) (e_k)^\beta (0)=0. \end{aligned}$$

The property (49) effectively implies that all the divergence forms in the expansion (50) below, are also equivalently non-divergence forms.

### Remark 12

The formula below is long, so let us first explain how one obtains it. Consider the first part of the diffusion generator $\mathcal {L}^N_D$ (18) (i.e. the part with covariance in it; the second part being more classical). It is in the form of a double sum, hence by linearity one can consider the action of each “sub-generator” $\frac{1}{2}\sigma ^2\nabla _{x_{i_0}}\cdot \big (C_\epsilon (\xi (x_{i_0}-x_{j_0}))\nabla _{x_{j_0}}F(\eta )\big )$, for each fixed pair of indices $(i_0,j_0)$. Note here that $i_0,j_0$ may not be distinct. One acts this “sub-generator” on our functional (48). Now, only those terms in (48) that contain either index $i_0$ or $j_0$ (or both) are affected by the action of this “sub-generator”. As we will exhaust all choices of $i_0, j_0$, for clarity we prefer to separate the case when $i_0=j_0$ from $i_0\ne j_0$. By the property $C_\epsilon (0)=I_3$, in the case $i_0=j_0$ we do not see the function $C_\epsilon$ appear since we have $I_3$. This is how we obtained the first 4 terms on the right-hand side of the explansion (50) below.

Similarly, the jump generator (19) is also a double sum. By linearity, one can consider the action of each “sub-generator” $\frac{1}{2}r_{i_0j_0}^N\left[ F(\eta ^{-i_0j_0})-F(\eta )\right]$, for each fixed pair of indices $(i_0,j_0)$ (here they are necessarily distinct). As one acts this “sub-generator” on our functional (48), only those terms that contain either index $i_0$ or $j_0$ (or both) are affected. Here, the effect of the action is that some terms of (48) are removed. A moment’s thought convinces oneself that triple sums may appear, as in the 6th term on the right-hand side of (50).

We get

50 $$\begin{aligned}&F_3\big (\eta _T^N\big )-F_3\big (\eta _0^N\big ) =\frac{1}{N^2}\int _0^T\sum _{i\in \mathcal {N}\big (\zeta _t^N\big )}\sum _{j\in \mathcal {N}\big (\zeta _t^N\big ): j\ne i}\frac{\sigma ^2}{2}\nonumber \\&\quad \Big [\nabla _{x_i}\cdot \big (C_\epsilon \big (\xi \big (x_i^N(t)-x_j^N(t)\big )\big )\nabla _{x_j}\big ( v^{\epsilon ,z}\big (x_i^N(t)-x_j^N(t)\big )\phi \big (x_i^N(t)\big )\psi \big (x_j^N(t)\big )\big )\big )\nonumber \\&\quad +\nabla _{x_i}\cdot \big (C_\epsilon \big (\xi \big (x_i^N(t)-x_j^N(t)\big )\big )\nabla _{x_j}\big ( v^{\epsilon ,z}\big (x_j^N(t)-x_i^N(t)\big )\phi \big (x^N_j(t)\big )\psi \big (x_i^N(t)\big )\big )\big )\Big ]dt\nonumber \\&\quad +\frac{1}{N^2}\int _0^T\sum _{i\in \mathcal {N}\big (\zeta _t^N\big )}\sum _{j\in \mathcal {N}\big (\zeta _t^N\big ): j\ne i}\frac{\sigma _0^2+\sigma ^2}{2}\Big [\Delta v^{\epsilon ,z}\big (x_i^N(t)-x_j^N(t)\big )\phi \big (x_i^N(t)\big )\psi \big (x_j^N(t)\big ) \nonumber \\&\quad +\Delta v^{\epsilon ,z}\big (x_j^N(t)-x_i^N(t)\big )\phi \big (x^N_j(t)\big )\psi \big (x_i^N(t)\big )\Big ]dt\nonumber \\&\quad +\frac{1}{N^2}\int _0^T\sum _{i\in \mathcal {N}\big (\zeta _t^N\big )}\sum _{j\in \mathcal {N}\big (\zeta _t^N\big ): j\ne i}\frac{\sigma _0^2+\sigma ^2}{2}\Big [v^{\epsilon ,z}\big (x_i^N(t)-x_j^N(t)\big )\Delta \phi \big (x_i^N(t)\big )\psi \big (x_j^N(t)\big )\nonumber \\&\quad +v^{\epsilon ,z}\big (x_j^N(t)-x_i^N(t)\big )\phi \big (x_j^N(t)\big )\Delta \psi \big (x_i^N(t)\big )\Big ]dt\nonumber \\&\quad +\frac{1}{N^2}\int _0^T\sum _{i\in \mathcal {N}\big (\zeta _t^N\big )}\sum _{j\in \mathcal {N}\big (\zeta _t^N\big ): j\ne i}\left( \sigma _0^2+\sigma ^2\right) \Big [\nabla v^{\epsilon ,z}\big (x_i^N(t)-x_j^N(t)\big )\cdot \nabla \phi \big (x_i^N(t)\big )\psi \big (x_j^N(t)\big )\nonumber \\&\quad -\nabla v^{\epsilon ,z}\big (x_j^N(t)-x_i^N(t)\big )\cdot \phi \big (x_j^N(t)\big )\nabla \psi \big (x_i^N(t)\big )\Big ]dt\nonumber \\&\quad -\int _0^T\sum _{i\in \mathcal {N}\big (\zeta _t^N\big )}\sum _{j\in \mathcal {N}\big (\zeta _t^N\big ): j\ne i}\frac{R_0}{2N}\theta ^\epsilon \big (x_i^N(t)-x_j^N(t)\big )\nonumber \\&\quad \cdot \frac{1}{N^2}\Big [v^{\epsilon ,z}\big (x_i^N(t)-x_j^N(t)\big )\phi \big (x_i^N(t)\big )\psi \big (x_j^N(t)\big )\nonumber \\&\quad +v^{\epsilon ,z}\big (x_j^N(t)-x_i^N(t)\big )\phi \big (x^N_j(t)\big )\psi \big (x_i^N(t)\big )\Big ]dt\nonumber \\&\quad -\int _0^T\sum _{i\in \mathcal {N}\big (\zeta _t^N\big )}\sum _{j\in \mathcal {N}\big (\zeta _t^N\big ): j\ne i}\frac{R_0}{2N}\theta ^\epsilon \big (x_i^N(t)-x_j^N(t)\big )\nonumber \\&\quad \cdot \frac{1}{N^2}\sum _{k\in \mathcal {N}\big (\zeta _t^N\big ):k\ne i,j}\Big [v^{\epsilon ,z}\big (x_i^N(t)-x_k^N(t)\big ) \phi \big (x_i^N(t)\big )\psi \big (x_k^N(t)\big )\nonumber \\&\quad +v^{\epsilon ,z}\big (x_k^N(t)-x_i^N(t)\big )\phi \big (x_k^N(t)\big )\psi \big (x_i^N(t)\big ) +v^{\epsilon ,z}\big (x_j^N(t)-x_k^N(t)\big )\phi \big (x_j^N(t)\big )\psi \big (x_k^N(t)\big )\nonumber \\&\quad +v^{\epsilon ,z}\big (x_k^N(t)-x_j^N(t)\big )\phi \big (x_k^N(t)\big )\psi (x_j^N(t)\big )\Big ]dt +\widetilde{M}_D^N(T)+\widetilde{M}_J^N(T), \end{aligned}$$

where $\widetilde{M}_D^N(t)$ and $\widetilde{M}_J^N(t)$ are two martingales associated with diffusion and jumps respectively.

With a tedious but straightforward computation (details omitted), we can expand and rewrite the 1st term on the right-hand side of (50) further:

$$\begin{aligned}&\frac{1}{N^2}\int _0^T\sum _{i\in \mathcal {N}\big (\zeta _t^N\big )}\sum _{j\in \mathcal {N}\big (\zeta _t^N\big ): j\ne i}\frac{\sigma ^2}{2} \big [\nabla _{x_i}\cdot \big (C_\epsilon \big (\xi \big (x_i^N(t)-x_j^N(t)\big )\big )\nabla _{x_j}\big ( v^{\epsilon ,z}\big (x_i^N(t)\\&\quad -x_j^N(t)\big )\phi \big (x_i^N(t)\big )\psi \big (x_j^N(t)\big )\big )\big )+\nabla _{x_i}\cdot \big (C_\epsilon \big (\xi \big (x_i^N(t)-x_j^N(t)\big )\big )\nabla _{x_j}\big ( v^{\epsilon ,z}\big (x_j^N(t)\\&\quad -x_i^N(t)\big )\phi \big (x^N_j(t)\big )\psi \big (x_i^N(t)\big )\big )\big )\big ]dt\\&=\frac{1}{N^2}\int _0^T\sum _{i\in \mathcal {N}\big (\zeta _t^N\big )}\sum _{j\in \mathcal {N}\big (\zeta _t^N\big ): j\ne i}\sigma ^2 \big [-\nabla _{x_i}\cdot \big (C _\epsilon \big (\xi \big (x_i^N(t)\\&\quad -x_j^N(t)\big )\big )\nabla v^{\epsilon ,z}\big (x_i^N(t)-x_j^N(t)\big )\big )\phi \big (x_i^N(t)\big )\psi \big (x_j^N(t)\big )\\&\quad - \nabla \phi \big (x_i^N(t)\big )^T C_\epsilon \big (\xi \big (x_i^N(t)-x_j^N(t)\big )\big )\nabla v^{\epsilon ,z}\big (x_i^N(t)-x_j^N(t)\big )\psi \big (x_j^N(t)\big )\\&\quad +\nabla v^{\epsilon ,z}\big (x_i^N(t)-x_j^N(t)\big )^T C_\epsilon \big (\xi \big (x_i^N(t)-x_j^N(t)\big )\big )\nabla \psi \big (x_j^N(t)\big )\phi \big (x_i^N(t)\big )\\&\quad + \nabla \phi \big (x_i^N(t)\big )^T C_\epsilon \big (\xi \big (x_i^N(t)-x_j^N(t)\big )\big )\nabla \psi \big (x_j^N(t)\big )v^{\epsilon ,z}\big (x_i^N(t)-x_j^N(t)\big )\big ]dt, \end{aligned}$$

in which we have used $C_\epsilon (-x)=C_\epsilon (x)^T$ and (49).

By the symmetry of $\theta$ and the preceding computation, we get the following simplified expansion from the action of generator to $F_3(\zeta _t^N)$:

51 $$\begin{aligned}&F_3\big (\eta _T^N\big )-F_3\big (\eta _0^N\big ) \end{aligned}$$

52 $$\begin{aligned}&=-\frac{\sigma ^2}{N^2}\int _0^T\sum _{i\in \mathcal {N}\big (\zeta _t^N\big )}\sum _{j\in \mathcal {N}\big (\zeta _t^N\big ): j\ne i}\nabla _{x_i}\cdot \big (C _\epsilon \big (\xi \big (x_i^N(t)-x_j^N(t)\big )\big )\nabla v^{\epsilon ,z}\big (x_i^N(t)-x_j^N(t)\big )\big )\nonumber \\&\quad \phi \big (x_i^N(t)\big )\psi \big (x_j^N(t)\big )dt \end{aligned}$$

53 $$\begin{aligned}&-\frac{\sigma ^2}{N^2}\int _0^T\sum _{i\in \mathcal {N}\big (\zeta _t^N\big )}\sum _{j\in \mathcal {N}\big (\zeta _t^N\big ): j\ne i} \nabla \phi \big (x_i^N(t)\big )^T C_\epsilon \big (\xi \big (x_i^N(t)-x_j^N(t)\big )\big )\nabla v^{\epsilon ,z}\nonumber \\&\quad \big (x_i^N(t)-x_j^N(t)\big )\psi \big (x_j^N(t)\big )dt \end{aligned}$$

54 $$\begin{aligned}&+\frac{\sigma ^2}{N^2}\int _0^T\sum _{i\in \mathcal {N}\big (\zeta _t^N\big )}\sum _{j\in \mathcal {N}\big (\zeta _t^N\big ): j\ne i}\nabla v^{\epsilon ,z}\big (x_i^N(t)-x_j^N(t)\big )^T C_\epsilon \big (\xi \big (x_i^N(t)\nonumber \\&-x_j^N(t)\big )\big )\nabla \psi \big (x_j^N(t)\big )\phi \big (x_i^N(t)\big )dt \end{aligned}$$

55 $$\begin{aligned}&+\frac{\sigma ^2}{N^2}\int _0^T\sum _{i\in \mathcal {N}\big (\zeta _t^N\big )}\sum _{j\in \mathcal {N}\big (\zeta _t^N\big ): j\ne i}\nabla \phi \big (x_i^N(t)\big )^T C_\epsilon \big (\xi \big (x_i^N(t)\nonumber \\&-x_j^N(t)\big )\big )\nabla \psi \big (x_j^N(t)\big )v^{\epsilon ,z}\big (x_i^N(t)-x_j^N(t)\big )dt \end{aligned}$$

56 $$\begin{aligned}&+\frac{\sigma _0^2+\sigma ^2}{N^2}\int _0^T\sum _{i\in \mathcal {N}\big (\zeta _t^N\big )}\sum _{j\in \mathcal {N}\big (\zeta _t^N\big ): j\ne i}\Delta v^{\epsilon ,z}\big (x_i^N(t)-x_j^N(t)\big )\phi \big (x_i^N(t)\big )\psi \big (x_j^N(t)\big )dt \end{aligned}$$

57 $$\begin{aligned}&+\frac{\sigma _0^2+\sigma ^2}{2N^2}\int _0^T\sum _{i\in \mathcal {N}\big (\zeta _t^N\big )}\sum _{j\in \mathcal {N}\big (\zeta _t^N\big ): j \ne i}v^{\epsilon ,z}\big (x_i^N(t)-x_j^N(t)\big )\nonumber \\&\quad \big [\Delta \phi \big (x_i^N(t)\big )\psi \big (x_j^N(t)\big )+\phi \big (x_j^N(t)\big )\Delta \psi \big (x_i^N(t)\big )\big ]dt \end{aligned}$$

58 $$\begin{aligned}&+\frac{\sigma _0^2+\sigma ^2}{N^2}\int _0^T\sum _{i\in \mathcal {N}\big (\zeta _t^N\big )}\sum _{j\in \mathcal {N}\big (\zeta _t^N\big ): j\ne i} \nabla v^{\epsilon ,z}\big (x_i^N(t)-x_j^N(t)\big )\cdot \nonumber \\&\quad \big [\nabla \phi \big (x_i^N(t)\big )\psi \big (x_j^N(t)\big )-\phi \big (x_j^N(t)\big )\nabla \psi \big (x_i^N(t)\big )\big ]dt \end{aligned}$$

59 $$\begin{aligned}&-\frac{R_0}{N^3}\int _0^T\sum _{i\in \mathcal {N}\big (\zeta _t^N\big )}\sum _{j\in \mathcal {N}\big (\zeta _t^N\big ): j\ne i}\theta ^\epsilon \big (x_i^N(t)-x_j^N(t)\big )v^{\epsilon ,z}\big (x_i^N(t)\nonumber \\&-x_j^N(t)\big )\phi \big (x_i^N(t)\big )\psi \big (x_i^N(t)\big )dt \end{aligned}$$

60 $$\begin{aligned}&-\frac{R_0}{N^3}\int _0^T\sum _{i\in \mathcal {N}\big (\zeta _t^N\big )}\sum _{j\in \mathcal {N}\big (\zeta _t^N\big ): j\ne i}\theta ^\epsilon \big (x_i^N(t)-x_j^N(t)\big )\nonumber \\&\quad \cdot \sum _{k\in \mathcal {N}\big (\zeta _t^N\big ):k\ne i,j}\big [v^{\epsilon ,z}\big (x_i^N(t)-x_k^N(t)\big )\phi \big (x_i^N(t)\big )\psi \big (x_k^N(t)\big )\nonumber \\&+v^{\epsilon ,z}\big (x_k^N(t)-x_i^N(t)\big )\phi \big (x_k^N(t)\big )\psi \big (x_i^N(t)\big )\big ]dt \end{aligned}$$

61 $$\begin{aligned}&+\widetilde{M}_D^N(T)+\widetilde{M}_J^N(T). \end{aligned}$$

We look for all the terms that contain the highest (i.e. second) partial derivatives of $v^{\epsilon ,z}$, which are (56) and (52). In a sense, they are the most dangerous in terms of regularity (not for fixed $\epsilon$ but as $\epsilon \rightarrow 0$). Their sum

$$\begin{aligned} (56)+(52)&=\frac{1}{N^2}\int _0^T\sum _{i\in \mathcal {N}\big (\zeta _t^N\big )}\sum _{j\in \mathcal {N}\big (\zeta _t^N\big ): j\ne i}\Big [\big (\sigma _0^2+\sigma ^2\big )\Delta v^{\epsilon ,z}\big (x_i^N(t)-x_j^N(t)\big )\\&\quad -\sigma ^2\nabla _{x_i}\big (C _\epsilon \big (\xi \big (x_i^N(t)-x_j^N(t)\big )\big )\nabla v^{\epsilon ,z}\big (x_i^N(t)-x_j^N(t)\big ) \big )\Big ]\\  &\qquad \phi \big (x_i^N(t)\big )\psi \big (x_j^N(t)\big )dt. \end{aligned}$$

Recall definiton of $v^{\epsilon ,z}$ in terms of $u^\epsilon$ (47), and use the cell equation (35) (which we repeat here)

$$\begin{aligned} (\sigma _0^2+\sigma ^2)\Delta u^\epsilon (x)&-\sigma ^{2}\nabla \cdot \left( C _\epsilon \left( \xi x\right) \nabla u^{\epsilon }\left( x\right) \right) =R_{0} \theta ^\epsilon \left( x\right) \Big ( 1+\frac{u^{\epsilon }\left( x\right) }{N} \Big )\\&{\text {with }}\quad x=x_i^N(t)-x_j^N(t) \end{aligned}$$

for the terms that involve $u^{\epsilon }(x_i^N(t)-x_j^N(t))$, while leaving those terms with $u^{\epsilon }(x_i^N(t)-x_j^N(t)+z)$ as they are, gives

$$\begin{aligned} (56)+(52)&=\frac{1}{N^2}\int _0^T\sum _{i\in \mathcal {N}\big (\zeta _t^N\big )}\sum _{j\in \mathcal {N}\big (\zeta _t^N\big ): j\ne i}\Big [\big (\sigma _0^2+\sigma ^2\big )\Delta u^{\epsilon }\big (x_i^N(t)-x_j^N(t)+z\big )\\&\quad -\sigma ^2\nabla _{x_i}\big (C _\epsilon \big (\xi \big (x_i^N(t)-x_j^N(t)\big )\big )\nabla u^{\epsilon }\big (x_i^N(t)-x_j^N(t)+z\big ) \big )\Big ]\\  &\qquad \phi \big (x_i^N(t)\big )\psi \big (x_j^N(t)\big )dt\\&\quad -\frac{R_0}{N^2}\int _0^T\sum _{i\in \mathcal {N}\big (\zeta _t^N\big )}\sum _{j\in \mathcal {N}\big (\zeta _t^N\big ): j\ne i}\theta ^\epsilon \big (x_i^N(t)-x_j^N(t)\big )\\&\quad \Big (1+\frac{1}{N}u^{\epsilon }\big (x_i^N(t)-x_j^N(t)\big ) \Big )\phi \big (x_i^N(t)\big )\psi \big (x_j^N(t)\big )dt. \end{aligned}$$

It turns out, a posteriori (but already noted in [13]), that a term like (59) with the same argument $x_i^N(t)-x_j^N(t)$ in both functions $\theta ^\epsilon$ and $u^\epsilon$ is not negligible, whereas if one argument is $x_i^N(t)-x_j^N(t)$ and the other is $x_i^N(t)-x_j^N(t)+z$, then such term is negligible [such as (63) and (64)]. Thus, we have to combine the above with the term (59) to “kill” a non-negligible term, and finally obtain

62 $$\begin{aligned}&(56)+(52)+(59)\nonumber \\&=\frac{1}{N^2}\int _0^T\sum _{i\in \mathcal {N}\big (\zeta _t^N\big )}\sum _{j\in \mathcal {N}\big (\zeta _t^N\big ): j\ne i} \Big [\big (\sigma _0^2+\sigma ^2\big )\Delta u^{\epsilon }\big (x_i^N(t)-x_j^N(t)+z\big )\nonumber \\&\quad -R_0\theta ^\epsilon \big (x_i^N(t)-x_j^N(t)\big )\Big ] \phi \big (x_i^N(t)\big )\psi \big (x_j^N(t)\big )dt \end{aligned}$$

63 $$\begin{aligned}&\quad -\frac{\sigma ^2}{N^2}\int _0^T\sum _{i\in \mathcal {N}\big (\zeta _t^N\big )}\sum _{j\in \mathcal {N}\big (\zeta _t^N\big ): j\ne i}\nabla _{x_i}\big (C _\epsilon \big (\xi \big (x_i^N(t)-x_j^N(t)\big )\big )\nabla u^{\epsilon }\big (x_i^N(t)-x_j^N(t)+z\big )\big )\nonumber \\&\qquad \phi \big (x_i^N(t)\big )\psi \big (x_j^N(t)\big )dt \end{aligned}$$

64 $$\begin{aligned}&\quad -\frac{R_0}{N^3}\int _0^T\sum _{i\in \mathcal {N}\big (\zeta _t^N\big )}\sum _{j\in \mathcal {N}\big (\zeta _t^N\big ): j\ne i}\theta ^\epsilon \big (x_i^N(t)-x_j^N(t)\big )u^{\epsilon }\big (x_i^N(t)\nonumber \\&\quad -x_j^N(t)+z\big )\phi \big (x_i^N(t)\big )\psi \big (x_j^N(t)\big )dt. \end{aligned}$$

The final product of all these manouvres is that in (62), we have made the pre-limit in (38) appear, and on the other hand, we can expect to show that all the rest of the whole Itô-Tanaka expansion, namely (51), (53), (54), (55), (57), (58), (60), (63), (64) as well as (65), (66) and (67) below, are all negligible in $L^1(\mathbb {P})$ in the limit first $N\rightarrow \infty$ and then $|z|\rightarrow 0$. This will be done in Sect. 6. Since the whole expansion (51)–(61) is an identity, the negligibility of all these “minor” terms will prove the desired negligibility of the “main” term in (38).

Finally, the martingales (61) should be bounded in $L^2(\mathbb {P})$ sense as follows.

$$\begin{aligned} \widetilde{M}_{D}^N(t)&:=\widetilde{M}_{D,1}^N(t)+\widetilde{M}_{D,2}^N(t)\\ \widetilde{M}_{D,1}^N(T)&:=\frac{\sigma }{N^2}\int _0^T\sum _{i\in \mathcal {N}\big (\zeta _t^N\big )}\sum _{j\in \mathcal {N}\big (\zeta _t^N\big ): j\ne i}\Big [\nabla v^{\epsilon ,z}\big (x_i^N(t)-x_j^N(t)\big )\phi \big (x_i^N(t)\big )\psi \big (x_j^N(t)\big )\\&\quad +v^{\epsilon ,z}\big (x_i^N(t)-x_j^N(t)\big )\nabla \phi \big (x_i^N(t)\big )\psi \big (x_j^N(t)\big )\\&\quad -\nabla v^{\epsilon ,z}\big (x_j^N(t)-x_i^N(t)\big )\phi \big (x_j^N(t)\big )\psi \big (x_i^N(t)\big ) \\&\quad +v^{\epsilon ,z}\big (x_j^N(t)-x_i^N(t)\big )\phi \big (x_j^N(t)\big )\nabla \psi \big (x_i^N(t)\big ) \Big ] \cdot \sum _{k\in \mathbb {N}}\lambda _ke_k^\epsilon \big (x_i^N(t)\big ) dW_k(t).\\ \widetilde{M}_{D,2}^N(T)&:=\frac{\sigma _0}{N^2}\int _0^T\sum _{i\in \mathcal {N}\big (\zeta _t^N\big )}\sum _{j\in \mathcal {N}\big (\zeta _t^N\big ): j\ne i}\Big [\nabla v^{\epsilon ,z}\big (x_i^N(t)-x_j^N(t)\big )\phi \big (x_i^N(t)\big )\psi \big (x_j^N(t)\big )\\&\quad +v^{\epsilon ,z}\big (x_i^N(t)-x_j^N(t)\big )\nabla \phi \big (x_i^N(t)\big )\psi \big (x_j^N(t)\big )\\&\quad -\nabla v^{\epsilon ,z}\big (x_j^N(t)-x_i^N(t)\big )\phi \big (x_j^N(t)\big )\psi \big (x_i^N(t)\big ) \\&\quad +v^{\epsilon ,z}\big (x_j^N(t)-x_i^N(t)\big )\phi \big (x_j^N(t)\big )\nabla \psi \big (x_i^N(t)\big ) \Big ]\cdot dB_i(t). \end{aligned}$$

Hence, by Itô isometry we can bound their quadratic variation by

65 $$\begin{aligned}&\mathbb {E}|\widetilde{M}_{D,1}^N(T)|^2\nonumber \\&\quad =\frac{\sigma ^2}{N^4}\int _0^T\sum _{k\in \mathbb {N}}\mathbb {E}\Bigg |\sum _{i\in \mathcal {N}\big (\zeta _t^N\big )}\sum _{j\in \mathcal {N}\big (\zeta _t^N\big ): j\ne i}\Big [\nabla v^{\epsilon ,z}\big (x_i^N(t)-x_j^N(t)\big )\phi \big (x_i^N(t)\big )\psi \big (x_j^N(t)\big )\nonumber \\&\quad +v^{\epsilon ,z}\big (x_i^N(t)-x_j^N(t)\big )\nabla \phi \big (x_i^N(t)\big )\psi \big (x_j^N(t)\big ) -\nabla v^{\epsilon ,z}\big (x_j^N(t)-x_i^N(t)\big )\phi \big (x_j^N(t)\big )\psi \big (x_i^N(t)\big )\nonumber \\&\quad +v^{\epsilon ,z}\big (x_j^N(t)-x_i^N(t)\big )\phi \big (x_j^N(t)\big )\nabla \psi \big (x_i^N(t)\big ) \Big ] \cdot \lambda _ke_k^\epsilon \big (x_i^N(t)\big )\Bigg |^2dt. \end{aligned}$$

66 $$\begin{aligned}&\mathbb {E}|\widetilde{M}_{D,2}^N(T)|^2\le \frac{2\sigma _0^2}{N^4}\sum _{i\in \mathcal {N}\big (\zeta _t^N\big )}\int _0^T\Bigg |\sum _{j\in \mathcal {N}\big (\zeta _t^N\big ): j\ne i}\Big [\nabla v^{\epsilon ,z}\big (x_i^N(t)-x_j^N(t)\big )\phi \big (x_i^N(t)\big )\psi \big (x_j^N(t)\big )\nonumber \\&\quad +v^{\epsilon ,z}\big (x_i^N(t)-x_j^N(t)\big )\nabla \phi \big (x_i^N(t)\big )\psi \big (x_j^N(t)\big ) -\nabla v^{\epsilon ,z}\big (x_j^N(t)-x_i^N(t)\big )\phi \big (x_j^N(t)\big )\psi \big (x_i^N(t)\big ) \nonumber \\&\quad +v^{\epsilon ,z}\big (x_j^N(t)-x_i^N(t)\big )\phi \big (x_j^N(t)\big )\nabla \psi \big (x_i^N(t)\big ) \Big ]\Bigg |^2 dt. \end{aligned}$$

Further, we can bound the second moment of the jump part of the martingale by the carré-du-champ formula cf. [6, Proposition 8.7]

67 $$\begin{aligned}&\mathbb {E}|\widetilde{M}_J^N(T)|^2\le \int _0^T\sum _{i\in \mathcal {N}\big (\zeta _t^N\big )}\sum _{j\in \mathcal {N}\big (\zeta _t^N\big ) : j\ne i}\frac{R_0}{N}\theta ^\epsilon \big (x_i^N(t)-x_j^N(t)\big )\nonumber \\&\quad \Bigg |\frac{1}{N^2}v^{\epsilon ,z} \big (x_i^N(t)-x_j^N(t)\big )\phi \big (x_i^N(t)\big )\psi \big (x_j^N(t)\big )\Bigg |^2dt \nonumber \\&\quad +\int _0^T\sum _{i\in \mathcal {N}\big (\zeta _t^N\big )}\sum _{j\in \mathcal {N}\big (\zeta _t^N\big ): j\ne i} \frac{R_0}{N}\theta ^\epsilon \big (x_i^N(t)-x_j^N(t)\big )\nonumber \\&\quad \Bigg |\frac{1}{N^2}\sum _{k\in \mathcal {N}\big (\zeta _t^N\big ): k\ne i,j} \Big [v^{\epsilon ,z}\big (x_i^N(t)-x_k^N(t)\big )\phi \big (x_i^N(t)\big ) \psi \big (x_k^N(t)\big )\nonumber \\&\quad +v^{\epsilon ,z}\big (x_k^N(t)-x_i^N(t)\big )\phi \big (x_k^N(t)\big ) \psi \big (x_i^N(t)\big )+v^{\epsilon ,z}\big (x_j^N(t)-x_k^N(t)\big ) \phi \big (x_j^N(t)\big )\psi \big (x_k^N(t)\big )\nonumber \\&\quad +v^{\epsilon ,z}\big (x_k^N(t)-x_j^N(t)\big )\phi \big (x_k^N(t)\big )\psi \big (x_j^N(t)\big )\Big ]\Bigg |^2dt \nonumber \\&=\frac{R_0}{N^5}\int _0^T\sum _{i\in \mathcal {N}\big (\zeta _t^N\big )}\sum _{j\in \mathcal {N}\big (\zeta _t^N\big ): j\ne i}\theta ^\epsilon \big (x_i^N(t) -x_j^N(t)\big )\big |v^{\epsilon ,z}\big (x_i^N(t)-x_j^N(t)\big )\phi \big (x_i^N(t)\big )\psi \big (x_j^N(t)\big )\big |^2dt \nonumber \\&\quad + \frac{4R_0}{N^5}\int _0^T\sum _{i\in \mathcal {N}\big (\zeta _t^N\big )}\sum _{j\in \mathcal {N}\big (\zeta _t^N\big ): j\ne i}\theta ^\epsilon \big (x_i^N(t)-x_j^N(t)\big )\nonumber \\&\quad \cdot \Bigg |\sum _{k\in \mathcal {N}\big (\zeta _t^N\big ): k\ne i,j}\Big [v^{\epsilon ,z}\big (x_i^N(t)-x_k^N(t)\big )\phi \big (x_i^N(t)\big )\psi \big (x_k^N(t)\big )\nonumber \\&\quad +v^{\epsilon ,z}\big (x_k^N(t) -x_i^N(t)\big )\phi \big (x_k^N(t)\big )\psi \big (x_i^N(t)\big )\Big ]\Bigg |^2dt. \end{aligned}$$

## The Negligibility of Various Terms

We set out to prove that all the terms (51), (53), (54), (55), (57), (58), (60), (63), (64), (65), (66) and (67) are negligible in the order of limits $\epsilon \rightarrow 0$ then $|z|\rightarrow 0$ (we refer to it simply as “negligible” in the sequel), which by the Itô–Tanaka identity (51)–(61) of Sect. 5 yields Proposition 38, and in turn Theorem 6.

Recall once more the $\epsilon$-dependent cell equation $u^\epsilon :\mathbb {R}^3\rightarrow \mathbb {R}$

68 $$\begin{aligned} \sigma _0^2\Delta u^\epsilon (x)+\sigma ^2\nabla \cdot \Big (\omega \Big (\frac{\xi x}{\epsilon }\Big ) \nabla u^{\epsilon }\left( x\right) \Big ) =R_{0} \theta ^\epsilon \left( x\right) \Big ( 1+\frac{u^{\epsilon }\left( x\right) }{N} \Big ), \end{aligned}$$

and the unscaled cell equation

69 $$\begin{aligned} \sigma _0^2\Delta u(x)+\sigma ^{2}\nabla \cdot \big (\omega (\xi x)\nabla u(x)\big ) =R_{0} \theta (x)\big ( 1+u(x) \big ). \end{aligned}$$

They are related by $u^\epsilon (x)=\epsilon ^{-1}u(x/\epsilon )$ for any $x\in \mathbb {R}^3$, hence whenever *u* exists, $u^\epsilon$ also exists. Crucial for our purpose are pointwise estimates of $u^\epsilon (x)$ stated in lemmas below. They are proved in [13, Lemma 3.5] in the case when the elliptic operator is $\Delta$. To generalize those estimates to our case of uniformly elliptic divergence-form operator with variable coefficients, we will derive all estimates for the $\epsilon$-independent *u*(*x*), and then transfer them to $u^\epsilon (x)$ by their scaling relation.

Denote the second-order divergence-form operator in the $\epsilon$-independent equation (69) by

$$\begin{aligned} \mathcal {A}_x&:=\sigma _0^2\Delta +\sigma ^2\nabla \cdot \left( \omega (\xi x)\nabla \right) -R_0\theta (x)\\&=\sigma _0^2\Delta +\sigma ^2{\text {Tr}}\left( \omega (\xi x)\nabla ^2 \right) -R_0\theta (x). \end{aligned}$$

(The equivalence between divergence and non-divergence form is due to (49).) It is uniformly elliptic, since for any $\xi \in \mathbb {R}^3$ and $x\in \mathbb {R}^3$

$$\begin{aligned} \xi ^T\left( \sigma _0^2I_3+\sigma ^2 \omega (\xi x)\right) \xi \ge \sigma _0^2|\xi |^2, \end{aligned}$$

where the matrix $\omega (\cdot )$ is nonnegative definite and $\sigma _0>0$.

Let us consider the parabolic operator $\partial _t-\mathcal {A}_x$, and let *p*(*t*; *x*, *y*) denote its fundamental solution (i.e. heat kernel). By parabolic regularity theory [15, Theorem 1, p. 483], *p*(*t*; *x*, *y*) and its first two spatial derivatives satisfy, for a constant *M* that depends only on $\sigma _0, \sigma , \xi , R_0$ and the $\mathsf C^\alpha$-norm of *a* and $\theta$ (these data are all given and fixed):

70 $$\begin{aligned} \begin{aligned} p(t; x,y)&\le \frac{M}{t^{3/2}}e^{-\frac{|x-y|^2}{Mt}},\\ |\nabla _x p(t; x,y)|&\le \frac{M}{t^{3/2+1/2}}e^{-\frac{|x-y|^2}{Mt}},\\ |\nabla _x^2p(t; x,y)|&\le \frac{M}{t^{3/2+1}}e^{-\frac{|x-y|^2}{Mt}}. \end{aligned} \end{aligned}$$

Since we are in $\mathbb {R}^3$, the Green function $g(x,y)>0$ (i.e. fundamental solution) of $\mathcal {A}_x$ exists and is related to the heat kernel by

$$\begin{aligned} g(x,y)=\int _0^\infty p(t;x,y)dt, \quad x, y\in \mathbb {R}^3. \end{aligned}$$

Thus, by (70) we have

71 $$\begin{aligned} g(x,y)\le \int _0^\infty \frac{M}{t^{3/2}}e^{-\frac{|x-y|^2}{Mt}}dt{\mathop {=}\limits ^{s=\frac{|x-y|^2}{t}}}M\int _0^\infty \frac{s^{-1/2}e^{-s/M}}{|x-y|}ds\le M'|x-y|^{-1}, \end{aligned}$$

for some finite constant $M'=M'(M)$, since $s^{-1/2}e^{-s/M}$ is integrable at both 0 and $\infty$. Similarly,

72 $$\begin{aligned} |\nabla _xg(x,y)|&\le \left| \int _0^\infty \nabla _x p(t;x,y)dt\right| \le \int _0^\infty |\nabla _xp(t;x,y)|dt\nonumber \\&\le \int _0^\infty \frac{M}{t^{3/2+1/2}}e^{-\frac{|x-y|^2}{Mt}}dt=M\int _0^\infty \frac{e^{-s/M}}{|x-y|^2}ds\le M'|x-y|^{-2}, \end{aligned}$$

73 $$\begin{aligned} |\nabla _x^2g(x,y)|&\le \left| \int _0^\infty \nabla _x^2 p(t;x,y)dt\right| \le \int _0^\infty |\nabla _x^2p(t;x,y)|dt\nonumber \\&\le \int _0^\infty \frac{M}{t^{3/2+1}}e^{-\frac{|x-y|^2}{Mt}}dt=M\int _0^\infty \frac{s^{1/2}e^{-s/M}}{|x-y|^3}ds\le M'|x-y|^{-3}, \end{aligned}$$

since $e^{-s/M}$ and $s^{1/2}e^{-s/M}$ are both integrable at both 0 and $\infty$.

Since (69) can be written as $\mathcal {A}_xu(x)=R_0\theta (x)$, one can find a solution given by

74 $$\begin{aligned} u(x)=-\int _{\mathbb {R}^3}g(x,y)R_0\theta (y)dy, \quad x\in \mathbb {R}^3. \end{aligned}$$

(The negative sign is just a convention, due to the fact that $\mathcal {A}_x$ is a negative operator while we take $g(x,y)>0$.) By elliptic regularity theory [17, Theorem 4.3.1], since $\theta \in \mathsf C^\alpha (\mathbb {R}^3)$ and coefficients of $\mathcal {A}_x$ are symmetric, $\mathsf C^\alpha$ and bounded, we have $u(x)\in \mathsf C^{2, \alpha }(\mathbb {R}^3)$.

### Lemma 13

There exists a finite constant $M=M(\sigma _0, \sigma , \xi , R_0, \omega , \theta )$ such that for all $x\in \mathbb {R}^3$ and $\epsilon \in (0,1)$, we have

75 $$\begin{aligned} |u^\epsilon (x)|&\le M\left( |x|\vee \epsilon \right) ^{-1}, \end{aligned}$$

76 $$\begin{aligned} |\nabla u^\epsilon (x)|&\le M\left( |x|\vee \epsilon \right) ^{-2}, \end{aligned}$$

and for all $|x|\ge 2\epsilon$ we have

77 $$\begin{aligned} |\nabla ^2 u^\epsilon (x)|&\le M|x|^{-3}. \end{aligned}$$

### Proof

We will first derive the estimate for *u*(*x*), then transfer them to $u^\epsilon (x)$. Since *u* solves $\mathcal {A}_xu(x)=R_0\theta (x)$, we can write

78 $$\begin{aligned} u(x)=-\int _{\mathbb {R}^3}g(x,y)R_0\theta (y)dy \end{aligned}$$

where *g*(*x*, *y*) is the Green function of $\mathcal {A}_x$. Recall that $\theta$ is nonnegative and has compact support in *B*(0, 1). If $|x|\ge 2$, then for any $y\in {\text {supp}}(\theta )$, $|x-y|\ge |x|-|y|\ge |x|-1\ge |x|/2$. Thus, by (71), $g(x,y)\le M'|x|^{-1}$. Hence, in this case

$$\begin{aligned} |u(x)|\le \int _{\mathbb {R}^3}g(x,y)R_0\theta (y)dy\le \int _{\mathbb {R}^3}M'|x|^{-1}R_0\theta (y)dy\le M'R_0 |x|^{-1}. \end{aligned}$$

If on the other hand, $|x|\le 2$, then for any $y\in {\text {supp}}(\theta )$, $x-y\in B(0,3)$, and hence

$$\begin{aligned} |u(x)|\le \int _{\mathbb {R}^3}g(x,y)R_0\theta (y)dy\le R_0\Vert \theta \Vert _{L^\infty } \int _{B(0,1)} M' |x-y|^{-1}dy\lesssim \int _{B(0,3)}|y|^{-1}dy\lesssim 1. \end{aligned}$$

Thus, we conclude the estimate

$$\begin{aligned} |u(x)|&\le M(|x|\vee 1)^{-1},\\ |u^\epsilon (x)|&=\epsilon ^{-1}|u(x/\epsilon )|\le M (|x|\vee \epsilon )^{-1}. \end{aligned}$$

Turning to the gradient estimate of *u*,

$$\begin{aligned} \nabla u(x) = -\int _{\mathbb {R}^3}\nabla _x g(x,y)R_0\theta (y)dy. \end{aligned}$$

Hence, if $|x|\ge 2$ then for any $y\in {\text {supp}}(\theta )$, $|x-y|\ge |x|/2$, and by (72),

$$\begin{aligned} |\nabla u(x)|&\le \int _{\mathbb {R}^3}|\nabla _x g(x,y)|R_0\theta (y)dy\le \int _{\mathbb {R}^3}M'|x|^{-2}R_0\theta (y)dy\le M'R_0 |x|^{-2}. \end{aligned}$$

If on the other hand, $|x|\le 2$, then for any $y\in {\text {supp}}(\theta )$, $x-y\in B(0,3)$, and hence

$$\begin{aligned} |\nabla u(x)|&\le \int _{\mathbb {R}^3}|\nabla _xg(x,y)|R_0\theta (y)dy\le R_0\Vert \theta \Vert _{L^\infty } \int _{B(0,1)} M' |x-y|^{-2}dy\\  &\lesssim \int _{B(0,3)}|y|^{-2}dy\lesssim 1. \end{aligned}$$

Thus, we conclude the estimate

$$\begin{aligned} |\nabla u(x)|&\le M(|x|\vee 1)^{-2},\\ |\nabla u^\epsilon (x)|&=\epsilon ^{-2}|u(x/\epsilon )|\le M (|x|\vee \epsilon )^{-2}. \end{aligned}$$

Turning to the estimate on the Hessian of *u*,

$$\begin{aligned} \nabla ^2 u(x) = -\int _{\mathbb {R}^3}\nabla _x^2 g(x,y)R_0\theta (y)dy. \end{aligned}$$

We only consider the case that $|x|\ge 2$, hence for any $y\in {\text {supp}}(\theta )$, $|x-y|\ge |x|/2$. By (73), we thus have that

$$\begin{aligned} |\nabla ^2 u(x)|&\le \int _{\mathbb {R}^3}|\nabla _x^2 g(x,y)|R_0\theta (y)dy\le \int _{\mathbb {R}^3}M'|x|^{-3}R_0\theta (y)dy\le M'R_0 |x|^{-3}. \end{aligned}$$

By scaling relation, this yields that for any *x* such that $|x|\ge 2\epsilon$,

$$\begin{aligned} |\nabla ^2 u^\epsilon (x)|&=\epsilon ^{-3}|u(x/\epsilon )|\le M|x|^{-3}. \end{aligned}$$

$$\square $$

### Lemma 14

There exists a finite constant $M=M(\sigma _0, \sigma , \xi , R_0, \omega , \theta )$ such that for all $x\in \mathbb {R}^3$ with $|x|\ge 2|z|+2\epsilon$, $\epsilon \in (0,1)$, we have

79 $$\begin{aligned} |u^\epsilon (x+z)-u^\epsilon (x)|\le M|z||x|^{-2}, \end{aligned}$$

80 $$\begin{aligned} |\nabla u^\epsilon (x+z)-\nabla u^\epsilon (x)|\le M|z||x|^{-3}. \end{aligned}$$

### Proof

We will first derive the estimate for *u*(*x*), then transfer them to $u^\epsilon (x)$. We consider only the regime $|x|\ge 2|z|+2$. By (78),

$$\begin{aligned} u(x+z)-u(x)=-\int _{\mathbb {R}^3}\left( g(x+z,y)-g(x,y)\right) R_0\theta (y)dy \end{aligned}$$

Since

$$\begin{aligned} g(x+z,y)-g(x,y)=\int _0^1\partial _sg(x+sz,y)ds=\int _0^1z\cdot \nabla _xg(x+sz,y)ds, \end{aligned}$$

we have

$$\begin{aligned} |g(x+z,y)-g(x,y)|\le \int _0^1|z\cdot \nabla _xg(x+sz,y)|ds\le |z|\sup _{s\in [0,1]}|\nabla _xg(x+sz,y)|. \end{aligned}$$

Since $|x|\ge 2|z|+2$, we have for any $y\in {\text {supp}}(\theta )$ and $s\in [0,1]$,

$$ |x+sz-y|\ge |x|-|sz|-|y|\ge |x|/2 +|x|/2-|z|-1\ge |x|/2. $$

Hence, by (72), for some finite constant *M*,

$$ |g(x+z,y)-g(x,y)|\le M|z||x|^{-2}. $$

Hence,

$$\begin{aligned} |u(x+z)-u(x)|\le \int _{\mathbb {R}^3}\left| g(x+z,y)-g(x,y)\right| R_0\theta (y)dy\le R_0M|z||x|^{-2}. \end{aligned}$$

By scaling relations, this implies that for any $|x|\ge 2|z|+2\epsilon$,

$$\begin{aligned} |u^\epsilon (x+z)-u^\epsilon (x)|=\epsilon ^{-1}|u(x/\epsilon +z/\epsilon )-u(x/\epsilon )|\lesssim \epsilon ^{-1}|z/\epsilon ||x/\epsilon |^{-2}=|z||x|^{-2}, \end{aligned}$$

where $|x/\epsilon |\ge 2|z/\epsilon |+2$ hence the above bound applies.

Turning to the estimates for the gradient, we have

$$\begin{aligned} \nabla u(x+z)-\nabla u(x)=-\int _{\mathbb {R}^3}\left( \nabla _xg(x+z,y)-\nabla _xg(x,y)\right) R_0\theta (y)dy. \end{aligned}$$

Since

$$\begin{aligned} \nabla _xg(x+z,y)-\nabla _xg(x,y)=\int _0^1\partial _s\nabla _xg(x+sz,y)ds=\int _0^1z\cdot \nabla _x^2g(x+sz,y)ds, \end{aligned}$$

we have

$$\begin{aligned} |\nabla _xg(x+z,y)-\nabla _xg(x,y)|\le \int _0^1|z\cdot \nabla _x^2g(x+sz,y)|ds\le |z|\sup _{s\in [0,1]}|\nabla _x^2g(x+sz,y)|. \end{aligned}$$

For $|x|\ge 2|z|+2$, any $y\in {\text {supp}}(\theta )$ and $s\in [0,1]$, we have $|x+sz-y|\ge |x|/2$, thus by (73) for some finite constant *M*, we have

$$ |\nabla _xg(x+z,y)-\nabla _xg(x,y)|\le M|z||x|^{-3}. $$

Hence,

$$\begin{aligned} |\nabla u(x+z)-\nabla u(x)|&\le \int _{\mathbb {R}^3}\left| \nabla _xg(x+z,y)-\nabla _xg(x,y)\right| R_0\theta (y)dy\\&\le R_0M|z||x|^{-2}\le R_0M|z||x|^{-3}. \end{aligned}$$

By scaling, this implies that for any $|x|\ge 2|z|+2\epsilon$,

$$\begin{aligned} |\nabla u^\epsilon (x+z)-\nabla u^\epsilon (x)|=\epsilon ^{-2}|\nabla u(x/\epsilon +z/\epsilon )-\nabla u(x/\epsilon )|\lesssim \epsilon ^{-2}|z/\epsilon ||x/\epsilon |^{-3}=|z||x|^{-3}. \end{aligned}$$

$$\square $$

### Lemma 15

The $\mathsf C^{2, \alpha }$-solution (74) of (69) satisfies $-1\le u(x)\le 0$ for all $x\in \mathbb {R}^3$. Hence $-N\le u^\epsilon (x)\le 0$.

### Proof

The argument is similar to [13, p. 64, Step 6]. Denote here

$$ \widetilde{\mathcal {A}}_x:=\sigma _0^2\Delta +\sigma ^2\nabla \cdot \left( \omega (\xi x)\nabla \right) $$

and (69) can be written as $\widetilde{\mathcal {A}}_xu(x)=\theta (x)(1+u(x))$. For each $\delta \in (0,1)$, let $\chi _\delta :\mathbb {R}\rightarrow [0,1]$ be smooth, identically 1 on $(-\infty , -\delta )$, identically 0 on $(0,\infty )$, and decreasing on $[-\delta ,0]$, and let

$$\begin{aligned} \phi _\delta (r) := (1+r)\chi _\delta (1+r), \end{aligned}$$

which is smooth and non-3ecreasing. Further assume that as $\delta \rightarrow 0$, $\chi _\delta \rightarrow 1_{(-\infty , 0)}$ in $\mathsf C^2$-norm. Clearly, we have the property that $|\phi _\delta (r)|\le 2r$ for $r\ge 1$ and $\phi '_\delta (r)\ge 0$.

Integration by parts in the ball *B*(0, *R*), $R\ge 1$, we have

$$\begin{aligned} \int _{B(0,R)}\phi _\delta (u(x))\widetilde{\mathcal {A}}_x u(x)du =&-\int _{B_R}\phi '_\delta (u(x))\nabla u(x)^T\left( \sigma _0^2 I_3+\sigma ^2\omega (\xi x)\right) \nabla u(x)dx\\  &+ \int _{\partial B(0,R)}\phi _\delta (u(x))\left( \sigma _0^2 I_3+\sigma ^2\omega (\xi x)\right) \nabla u(x)\cdot d\textbf{n}(x), \end{aligned}$$

where $\textbf{n}(x)$ denotes the unit outward normal vector. By (75) and (76),

$$\begin{aligned} \left| \int _{\partial B(0,R)}\phi _\delta (u(x))\left( \sigma _0^2 I_3+\sigma ^2\omega (\xi x)\right) \nabla u(x)\cdot d\textbf{n}(x)\right| \lesssim R^{-1}R^{-2}|\partial B(0,R)|=O(R^{-1}). \end{aligned}$$

Hence taking $R\rightarrow \infty$, we obtain that

81 $$\begin{aligned} \int _{\mathbb {R}^3}\phi _\delta (u(x))\widetilde{\mathcal {A}}_x u(x)du =&-\int _{\mathbb {R}^3}\phi '_\delta (u(x))\nabla u(x)^T\left( \sigma _0^2 I_3+\sigma ^2\omega (\xi x)\right) \nabla u(x)dx\le 0 . \end{aligned}$$

On the other hand,

$$\begin{aligned} \int _{\mathbb {R}^3}\phi _\delta (u(x))\widetilde{\mathcal {A}}_x u(x)du&=\int _{\mathbb {R}^3}\phi _\delta (u(x))\theta (x)\left( 1+u(x)\right) dx\\&=\int _{\mathbb {R}^3}\theta (x)\left( 1+u(x)\right) ^2\chi _\delta (1+u(x))dx\ge 0, \end{aligned}$$

hence in view of (81) the right-hand side is 0. Letting $\delta \rightarrow 0$ in (81), since $\phi '_\delta \rightarrow 1_{(-\infty , -1)}$ by construction, we get

$$\begin{aligned} \int _{\mathbb {R}^3}1_{\{u(x)\le -1\}}\nabla u(x)^T\left( \sigma _0^2 I_3+\sigma ^2\omega (\xi x)\right) \nabla u(x)dx=0. \end{aligned}$$

Since the quadratic form in the above expression is positive unless $\nabla u(x)=0$, we conclude that $\nabla u(x)=0$ on the set $\{x\in \mathbb {R}^3: u(x)\le -1\}$. Since *u* is continuous, we must have $u(x)=-1$ on this set, and so $u(x)\ge -1$ for all $x\in \mathbb {R}^3$. Further, by (74) and $g(x,y)>0$, we have $u(x)\le 0$ for all $x\in \mathbb {R}^3$. $\square$

Now we proceed to bound all terms that are expected to be negligible. We will make extensive use of the auxiliary free particle system $\{y_i^\epsilon \}_{i=1}^\infty$ (32) introduced in Sect. 3. Recall that under a natural coupling, whenever a true particle $x_i^N$ is active by time *t*, its trajectory on [0, *t*] must coincide with that of the free particle $y_i^\epsilon$. We start with the term (64).

### Proposition 16

82 $$\begin{aligned}&\lim _{|z|\rightarrow 0}\limsup _{\epsilon \rightarrow 0}\mathbb {E}\Bigg |\frac{1}{N^3}\int _0^T\sum _{i\in \mathcal {N}(\zeta _t^N)}\sum _{j\in \mathcal {N}(\zeta _t^N): j\ne i}\theta ^\epsilon (x_i^N(t)-x_j^N(t))u^{\epsilon }(x_i^N(t)\nonumber \\&\quad -x_j^N(t)+z)\phi (x_i^N(t))\psi (x_j^N(t))dt\Bigg |=0. \end{aligned}$$

### Proof

Since the limit $|z|\rightarrow 0$ is taken only after $\epsilon \rightarrow 0$, we can always assume that $|z|\ge 2\epsilon$. The pre-limit on the left-hand side of (82) can be bounded from above by

$$\begin{aligned}&\le \frac{1}{N^3}\mathbb {E}\int _0^T\sum _{i\in \mathcal {N}(\zeta _t^N)}\sum _{j\in \mathcal {N}(\zeta _t^N): j\ne i}\theta ^\epsilon (x_i^N(t)\\&\quad -x_j^N(t))|u^{\epsilon }(x_i^N(t)-x_j^N(t)+z)||\phi (x_i^N(t))||\psi (x_j^N(t))|dt. \end{aligned}$$

Since the integrand and summands are nonnegative, we can further bound it above by

$$\begin{aligned}&\le \frac{1}{N^3}\mathbb {E}\int _0^T\sum _{i\ne j=1}^N\theta ^\epsilon (y_i^\epsilon (t)-y_j^\epsilon (t))|u^{\epsilon }(y_i^\epsilon (t)-y_j^\epsilon (t)+z)||\phi (y^\epsilon _i(t))||\psi (y^\epsilon _j(t))|dt. \end{aligned}$$

Using exchangeability of the free particles, we further have that

$$\begin{aligned}&\le \frac{1}{N}\mathbb {E}\int _0^T\theta ^\epsilon (y_1^\epsilon (t)-y_2^\epsilon (t))|u^{\epsilon }(y_1^\epsilon (t)-y_2^\epsilon (t)+z)||\phi (y^\epsilon _1(t))||\psi (y^\epsilon _2(t))|dt\\&\le \frac{1}{N}\int _0^T\iint _{(\mathbb {R}^3)^2}\theta ^\epsilon (y_1-y_2)|u^{\epsilon }(y_1-y_2+z)|f_t^{12,\epsilon }(y_1,y_2)|\phi (y_1)\psi (y_2)|dy_1dy_2dt\\&\le C_0T\frac{1}{N}\iint _{(\mathbb {R}^3)^2}\theta ^\epsilon (y_1-y_2)|u^{\epsilon }(y_1-y_2+z)||\phi (y_1)\psi (y_2)|dy_1dy_2, \end{aligned}$$

where $f_t^{12,\epsilon }(y_1,y_2)$ denotes the joint density of $(y_1^\epsilon (t), y_2^\epsilon (t))$ which satisfies the bound (33). Since $|y_1-y_2|\le \epsilon$ in the support of the function $\theta ^\epsilon$, and $|z|\ge 2\epsilon$, by (75) we have

$$\begin{aligned} |u^{\epsilon }(y_1-y_2+z)| \lesssim |z|^{-1}. \end{aligned}$$

Further, $\iint _{(\mathbb {R}^3)^2}\theta ^\epsilon (y_1-y_2)|\phi (y_1)\psi (y_2)|dy_1dy_2\lesssim 1$, $N=\epsilon ^{-1}$, hence we have

$$\begin{aligned} \frac{1}{N}\iint _{(\mathbb {R}^3)^2}\theta ^\epsilon (y_1-y_2)|u^{\epsilon }(y_1-y_2+z)|dy_1dy_2\lesssim \epsilon |z|^{-1}. \end{aligned}$$

This yields (82). $$\square $$

We continue with (63).

### Proposition 17

83 $$\begin{aligned}&\lim _{|z|\rightarrow 0}\limsup _{\epsilon \rightarrow 0} \mathbb {E}\Bigg |\frac{1}{N^2}\int _0^T\sum _{i\in \mathcal {N}(\zeta _t^N)}\sum _{j\in \mathcal {N}(\zeta _t^N): j\ne i}\nabla \cdot \nonumber \\&\quad \big (C _\epsilon \big (x_i^N(t)-x_j^N(t)\big )\nabla u^{\epsilon }(x_i^N(t)-x_j^N(t)+z)\big )\phi (x_i^N(t))\psi (x_j^N(t))dt\Bigg |=0. \end{aligned}$$

### Proof

Since the limit $|z|\rightarrow 0$ is taken only after $\epsilon \rightarrow 0$, we can always assume that $|z|\ge 2\epsilon$. In view of (49), the pre-limit on the left-hand side of (83) can be bounded from above as follows

$$\begin{aligned}&=\mathbb {E}\Bigg |\frac{1}{N^2}\int _0^T\sum _{i\in \mathcal {N}\big (\zeta _t^N\big )}\sum _{j\in \mathcal {N}\big (\zeta _t^N\big ): j\ne i} \sum _{\alpha ,\beta =1}^3C _\epsilon ^{\alpha \beta }\big (x_i^N(t)-x_j^N(t)\big )\partial ^2_{\alpha \beta } u^{\epsilon }\big (x_i^N(t)-x_j^N(t)+z\big )\\&\qquad \phi \big (x_i^N(t)\big )\psi \big (x_j^N(t)\big )dt\Bigg |\\&\quad \le \frac{1}{N^2}\mathbb {E}\int _0^T\sum _{i\in \mathcal {N}\big (\zeta _t^N\big )}\sum _{j\in \mathcal {N}\big (\zeta _t^N\big ): j\ne i}\sum _{\alpha ,\beta =1}^3\big |C _\epsilon ^{\alpha \beta }\big (x_i^N(t)-x_j^N(t)\big )\big |\big |\\&\qquad \partial ^2_{\alpha \beta }u^{\epsilon }\big (x_i^N(t)-x_j^N(t)+z\big )\big |\big |\phi \big (x_i^N(t)\big )\psi \big (x_j^N(t)\big )\big |dt\\&\quad \le \frac{1}{N^2}\mathbb {E}\int _0^T\sum _{i\ne j=1}^N\sum _{\alpha ,\beta =1}^3\big |C _\epsilon ^{\alpha \beta }\big (y_i^\epsilon (t)-y_j^\epsilon (t)\big )\big |\big |\partial ^2_{\alpha \beta }u^{\epsilon }\big (y_i^\epsilon (t)\\&\qquad -y_j^\epsilon (t)+z\big )\big |\big |\phi \big (y_i^\epsilon (t)\big )\psi \big (y_j^\epsilon (t)\big )|dt\\&\quad \le \mathbb {E}\int _0^T\sum _{\alpha ,\beta =1}^3\big |C _\epsilon ^{\alpha \beta }\big (y_1^\epsilon (t)-y_2^\epsilon (t)\big )\big |\big |\partial ^2_{\alpha \beta }u^{\epsilon }\big (y_1^\epsilon (t)-y_2^\epsilon (t)+z\big )\big |\big |\phi \big (y_1^\epsilon (t)\big )\psi \big (y_2^\epsilon (t)\big )\big |dt\\&\quad \le C_0T\iint _{(\mathbb {R}^3)^2}\sum _{\alpha ,\beta =1}^3|C _\epsilon ^{\alpha \beta }\big (y_1-y_2\big )\big |\big |\partial ^2_{\alpha \beta }u^{\epsilon }(y_1-y_2+z)\big |\big |\phi (y_1)\psi (y_2)|dy_1dy_2, \end{aligned}$$

using the auxiliary free system, its exchangeability, and that $(y^\epsilon _1(t), y^\epsilon _2 (t))$ has a uniformly bounded density, see Proposition 7. Since we have assumed that $C _\epsilon (x)=C (x/\epsilon )$ for some matrix function *C* with compact support in $B(0,1)\subset \mathbb {R}^3$, we have $|y_1-y_2|\le \epsilon$ in the support of $C _\epsilon ^{\alpha \beta }$ in the above display. By (77) and since $|z| \ge 2\epsilon$, we have

$$\begin{aligned} |\partial ^2_{\alpha \beta }u^{\epsilon }(y_1-y_2+z)|&\lesssim |z|^{-3}, \quad \alpha ,\beta =1,2,3. \end{aligned}$$

Thus, we have

$$\begin{aligned}&\iint _{(\mathbb {R}^3)^2}\sum _{\alpha ,\beta =1}^3\big |\partial ^2_{\alpha \beta }u^{\epsilon }(y_1-y_2+z)\big |\big |C _\epsilon ^{\alpha \beta }(y_1-y_2)\big |\big |\phi (y_1)\psi (y_2)\big |dy_1dy_2\\&\lesssim |z|^{-3}\iint \sum _{\alpha ,\beta =1}^3\big |C _\epsilon ^{\alpha \beta }(y)\big |\big |\phi (y+y_2)\psi (y_2)\big |dydy_2\lesssim |z|^{-3}\int \sum _{\alpha ,\beta =1}^3\big |C _\epsilon ^{\alpha \beta }(y)\big |dy\\&=\epsilon ^3|z|^{-3}\int \sum _{\alpha ,\beta =1}^3\big |C ^{\alpha \beta }(y)|dy, \end{aligned}$$

where the factor $\epsilon ^3$ comes from change of variables. This yields (83) since the function *C* is smooth and compactly supported. $\square$

We continue with (57). There are two terms with similar structure, and it suffices to consider one of them.

### Proposition 18

84 $$\begin{aligned}&\lim _{|z|\rightarrow 0}\limsup _{\epsilon \rightarrow 0}\nonumber \\&\quad \mathbb {E}\Bigg |\frac{1}{N^2}\int _0^T\sum _{i\in \mathcal {N}\big (\zeta _t^N\big )}\sum _{j\in \mathcal {N}\big (\zeta _t^N\big ): j\ne i}\big [u^{\epsilon }\big (x_i^N(t)-x_j^N(t)+z\big )-u^{\epsilon }\big (x_i^N(t)-x_j^N(t)\big )\big ]\nonumber \\&\quad \Delta \phi \big (x_i^N(t)\big )\psi \big (x_j^N(t)\big )dt \Bigg |=0. \end{aligned}$$

### Proof

The pre-limit on the left-hand side of (84) can be bounded from above by

$$\begin{aligned}&\le \frac{1}{N^2}\mathbb {E}\int _0^T\sum _{i\in \mathcal {N}\big (\zeta _t^N\big )}\sum _{j\in \mathcal {N}\big (\zeta _t^N\big ): j\ne i}\big |u^{\epsilon }\big (x_i^N(t)-x_j^N(t)+z\big )-u^{\epsilon }\big (x_i^N(t)-x_j^N(t)\big )\big |\big |\\&\qquad \Delta \phi \big (x_i^N(t)\big )\psi \big (x_j^N(t)\big )\big |dt\\&\quad \le \frac{1}{N^2}\mathbb {E}\int _0^T\sum _{i\ne j=1}^N\big |u^{\epsilon }\big (y_i^\epsilon (t)-y_j^\epsilon (t)+z\big )-u^{\epsilon }\big (y_i^\epsilon (t)-y_j^\epsilon (t)\big )\big |\big |\Delta \phi \big (y_i^\epsilon (t)\big )\psi \big (y_j^\epsilon (t)\big )\big |dt\\&\quad \le \mathbb {E}\int _0^T\big |u^{\epsilon }\big (y_1^\epsilon (t)-y_2^\epsilon (t)+z\big )-u^{\epsilon }\big (y_1^\epsilon (t)-y_2^\epsilon (t)\big )\big |\big |\Delta \phi \big (y_1^\epsilon (t)\big )\psi \big (y_2^\epsilon (t)\big )\big |dt\\&\quad \le C_0T\iint _{(\mathbb {R}^3)^2}\big |u^{\epsilon }(y_1-y_2+z)-u^{\epsilon }(y_1-y_2)\big |\big |\Delta \phi (y_1)\psi (y_2)\big |dy_1dy_2\\&\quad =C_0T\iint _{(\mathbb {R}^3)^2}\big |u^{\epsilon }(y+z)-u^{\epsilon }(y)\big |\big |\Delta \phi (y+y_2)\psi (y_2)\big |dydy_2, \end{aligned}$$

using the auxiliary free system, its exchangeability, and that $(y^\epsilon _1(t), y^\epsilon _2 (t))$ has a uniformly bounded density, see Proposition 7. By (79) and (75), we have

$$\begin{aligned} \big |u^{\epsilon }(y+z)-u^{\epsilon }(y)\big |\lesssim {\left\{ \begin{array}{ll} |z||y|^{-2}, \quad {\text {if }}|y|\ge 2|z|+2\epsilon ,\\ (\epsilon \vee |y+z|)^{-1}+(\epsilon \vee |y|)^{-1}, \quad {\text {for all }}y. \end{array}\right. } \end{aligned}$$

Note that for any $y_2\in {\text {supp}}(\psi )$, $y+y_2\in {\text {supp}}(\phi )$, we have $y\in \mathbb K$ for some compact set $\mathbb {K}$ which is determined by the supports of $\phi ,\psi$. Hence, bounding $|\Delta \phi (y+y_2)|\le \Vert \phi \Vert _{C^2}$, we have

85 $$\begin{aligned} \int _{\mathbb {K}}\big |u^{\epsilon }(y+z)-u^{\epsilon }(y)\big | dy&\lesssim \int _{B(0,2|z|+2\epsilon )}(\epsilon \vee |y+z|)^{-1}dy\nonumber \\&\quad +\int _{B(0,2|z|+2\epsilon )}(\epsilon \vee |y|)^{-1}dy\nonumber \\&\quad +\int _{\mathbb {K}\backslash B(0,2|z|+2\epsilon )}|z||y|^{-2}dy \nonumber \\&\le 2\int _{B(0,3|z|+2\epsilon )}(\epsilon \vee |y|)^{-1}dy+\int _{\mathbb {K}\backslash B(0,2|z|+2\epsilon )}|z||y|^{-2}dy\nonumber \\&\lesssim (3|z|+2\epsilon )^2+|z|. \end{aligned}$$

Then we integrate in $|\psi (y_2)|dy_2$ which is finite. This yields (84). $\square$

### Remark 19

The same proof as above also yields the negligibility of the initial $F_3(\eta _0^N)$ and terminal $F_3(\eta _T^N)$ terms in (51) individually, since each of them also involve the difference of $u^\epsilon$ and the heat kernel estimates are uniform in [0, *T*]. Thus we omit discussing them.

We continue with (58). There are two terms with similar structure, and it suffices to consider one of them.

### Proposition 20

86 $$\begin{aligned}&\lim _{|z|\rightarrow 0}\limsup _{\epsilon \rightarrow 0}\nonumber \\&\quad \mathbb {E}\Bigg |\frac{1}{N^2}\int _0^T\sum _{i\in \mathcal {N}\big (\zeta _t^N\big )}\sum _{j\in \mathcal {N}\big (\zeta _t^N\big ): j\ne i}\big [\nabla u^{\epsilon }\big (x_i^N(t)-x_j^N(t)+z\big )-\nabla u^{\epsilon }\big (x_i^N(t)-x_j^N(t)\big )\big ]\cdot \nonumber \\&\quad \nabla \phi \big (x_i^N(t)\big )\psi \big (x_j^N(t)\big )dt\Bigg |=0. \end{aligned}$$

### Proof

The pre-limit on the left-hand side of (86) can be bounded from above by

$$\begin{aligned}&\le \frac{1}{N^2}\mathbb {E}\int _0^T\sum _{i\in \mathcal {N}\big (\zeta _t^N\big )}\sum _{j\in \mathcal {N}\big (\zeta _t^N\big ): j\ne i}\\&\qquad \big |\nabla u^{\epsilon }\big (x_i^N(t)-x_j^N(t)+z\big )-\nabla u^{\epsilon }\big (x_i^N(t)-x_j^N(t)\big )\big |\big |\nabla \phi \big (x_i^N(t)\big )\psi \big (x_j^N(t)\big )\big |dt\\&\quad \le \frac{1}{N^2}\mathbb {E}\int _0^T\sum _{i\ne j =1}^N\big |\nabla u^{\epsilon }\big (y_i^\epsilon (t)-y_j^\epsilon (t)+z\big )-\nabla u^{\epsilon }\big (y_i^\epsilon (t)-y_j^\epsilon (t)\big )\big |\big |\nabla \phi \big (y_i^\epsilon (t)\big )\psi \big (y_j^\epsilon (t)\big )\big |dt\\&\quad \le \mathbb {E}\int _0^T\big |\nabla u^{\epsilon }\big (y_1^\epsilon (t)-y_2^\epsilon (t)+z\big )-\nabla u^{\epsilon }\big (y_1^\epsilon (t)-y_2^\epsilon (t)\big )\big |\big |\nabla \phi \big (y_1^\epsilon (t)\big )\psi \big (y_2^\epsilon (t)\big )\big |dt\\&\quad \le C_0T\iint _{(\mathbb {R}^3)^2}\big |\nabla u^{\epsilon }(y_1-y_2+z)-\nabla u^{\epsilon }(y_1-y_2)\big |\big |\nabla \phi (y_1)\psi (y_2)\big |dy_1dy_2\\&\quad =C_0T\iint _{(\mathbb {R}^3)^2}\big |\nabla u^{\epsilon }(y+z)-\nabla u^{\epsilon }(y)\big |\big |\nabla \phi (y+y_2)\psi (y_2)\big |dy, \end{aligned}$$

using the auxiliary free system, its exchangeability, and that $(y^\epsilon _1(t), y^\epsilon _2 (t))$ has a uniformly bounded density, see Proposition 7. By (80) and (76), we have

$$\begin{aligned} \big |\nabla u^{\epsilon }(y+z)-\nabla u^{\epsilon }(y)\big |\lesssim {\left\{ \begin{array}{ll} |z||y|^{-3}, \quad {\text {if }}|y|\ge 2|z|+2\epsilon ,\\ (\epsilon \vee |y+z|)^{-2}+(\epsilon \vee |y|)^{-2}, \quad {\text {for all }}y.\end{array}\right. } \end{aligned}$$

Hence, for some $r>2|z|+2\epsilon$ to be chosen and a compact set $\mathbb {K}$ determined by the supports of $\phi ,\psi$, we have

$$\begin{aligned}&\int _{\mathbb {K}}\big |\nabla u^{\epsilon }(y+z)-\nabla u^{\epsilon }(y)\big ||\nabla \phi (y+y_2)|dy\\&\lesssim \int _{B(0,r)}(\epsilon \vee |y+z|)^{-2}dy+\int _{B(0,r)}(\epsilon \vee |y|)^{-2}dy+\int _{\mathbb {K}\backslash B(0,r)}|z||y|^{-3}dy\\&\le 2\int _{B(0,|z|+r)}(\epsilon \vee |y|)^{-2}dy+\int _{\mathbb {K}\backslash B(0,r)}|z||y|^{-3}dy\\&\lesssim (|z|+r)+|z|r^{-3}|\mathbb {K}|. \end{aligned}$$

Choosing $r=4|z|^{\frac{1}{4}}>2|z|+2\epsilon$ (we can always assume $2\epsilon \le |z|\le 1/2$) yields

87 $$\begin{aligned} \int _{\mathbb {K}}\big |\nabla u^{\epsilon }(y+z)-\nabla u^{\epsilon }(y)\big ||\nabla \phi (y+y_2)|dy\lesssim |z|^{\frac{1}{4}}. \end{aligned}$$

Then we integrate $|\psi (y_2)|dy_2$ which is finite. This yields (86). $\square$

### Remark 21

The same proof for (86) can also yield the negligibility of the terms (53) and (54), since one can simply bound the covariance terms $\max _{\alpha ,\beta =1}^3|C^{\alpha ,\beta }_\epsilon (\xi (x_i^N(t)-x_j^N(t))|\le \Vert C\Vert _{L^\infty }:=\max _{\alpha ,\beta =1}^3 \Vert C^{\alpha ,\beta }\Vert _{L^\infty (\mathbb {R}^3)}$ and then they are in the form of an averaged double sum involving the difference of $\nabla u^\epsilon$. It is not necessary to take advantage of the smallness given by the support of $C_\epsilon$. Similarly, the proof given to prove (84) can yield the negligibility of the term (55), since it also involves the difference of $u^\epsilon$, and one can bound $\max _{\alpha ,\beta =1}^3|C^{\alpha ,\beta }_\epsilon (\xi (x_i^N(t)-x_j^N(t))|\le \Vert C\Vert _{L^\infty }$.

We continue with (60). Although there are two terms there, they can be bounded in the same way, hence it suffices to consider one of them.

### Proposition 22

88 $$\begin{aligned}&\lim _{|z|\rightarrow 0}\limsup _{\epsilon \rightarrow 0}\mathbb {E}\Bigg |\frac{1}{N^3}\int _0^T \sum _{i,j,k\in \mathcal {N}\big (\zeta _t^N\big ): j\ne i, k\ne i,j}\theta ^\epsilon \big (x_i^N(t)-x_j^N(t)\big ) \nonumber \\&\quad \cdot \big [u^{\epsilon }\big (x_i^N(t)-x_k^N(t)+z\big )-u^{\epsilon }\big (x_i^N(t)-x_k^N(t)\big )\big ]\phi \big (x_i^N(t)\big )\psi \big (x_k^N(t)\big )dt\Bigg |=0. \end{aligned}$$

### Proof

The pre-limit on the left-hand side of (88) can be bounded from above by

$$\begin{aligned}&\frac{1}{N^3}\mathbb {E}\int _0^T\sum _{i,j,k\in \mathcal {N}\big (\zeta _t^N\big ): j\ne i, k\ne i,j}\theta ^\epsilon \big (x_i^N(t)-x_j^N(t)\big )\big |u^{\epsilon }\big (x_i^N(t)-x_k^N(t)+z\big )\\&\qquad -u^{\epsilon }\big (x_i^N(t)-x_k^N(t)\big )\big |\big | \phi \big (x_i^N(t)\big )\psi \big (x_k^N(t)\big )\big |dt\\&\quad \le \frac{1}{N^3}\mathbb {E}\int _0^T\sum _{i,j,k=1}^N1_{\{j\ne i, k\ne i,j\}}\theta ^\epsilon \big (y_i^\epsilon (t)-y_j^\epsilon (t)\big )\big |u^{\epsilon }\big (y_i^\epsilon (t)-y_k^\epsilon (t)+z\big )\\&\qquad -u^{\epsilon }\big (y_i^\epsilon (t)-y_k^\epsilon (t)\big )\big |\big | \phi (y_i^\epsilon (t))\psi \big (y_k^\epsilon (t)\big )\big |dt\\&\quad \le \mathbb {E}\int _0^T\theta ^\epsilon \big (y_1^N(t)-y_3^N(t)\big )\big |u^{\epsilon }\big (y_1^\epsilon (t)-y_2^\epsilon (t)+z\big )-u^{\epsilon }\big (y_1^\epsilon (t)\\&\qquad -y_2^\epsilon (t)\big )\big |\big |\phi \big (y_1^\epsilon (t)\big )\psi \big (y_2^\epsilon (t)\big )\big |dt, \end{aligned}$$

using the auxiliary free system and its exchangeability. Since the triple $(y_1^\epsilon (t), y_2^\epsilon (t), y_3^\epsilon (t))$ has a joint density $f_t^{123, \epsilon }(y_1,y_2,y_3)$ that satisfies the bound (33), we can further bound the previous display by

$$\begin{aligned}&=\int _0^T\iiint _{(\mathbb {R}^3)^3}\theta ^\epsilon (y_1-y_3)\big |u^{\epsilon }(y_1-y_2+z)-u^{\epsilon }(y_1-y_2)\big |\big |\\&\qquad \phi (y_1)\psi (y_2)\big |f_t^{123,\epsilon }(y_1,y_2,y_3)dy_1dy_2dy_3dt\\&\quad \le C_0T\iiint _{(\mathbb {R}^3)^3}\theta ^\epsilon (y_1-y_3)\big |u^{\epsilon }(y_1-y_2+z)-u^{\epsilon }(y_1-y_2)\big |\big |\phi (y_1)\psi (y_2)\big |dy_1dy_2dy_3\\&\quad =c_0T\iint _{(\mathbb {R}^3)^2}\big |u^{\epsilon }(y_1-y_2+z)-u^{\epsilon }(y_1-y_2)\big |\big |\phi (y_1)\psi (y_2)\big |dy_1dy_2\\&\quad =C_0T\iint _{(\mathbb {R}^3)^2}\big |u^{\epsilon }(y+z)-u^{\epsilon }(y)\big |\big |\phi (y+y_2)\psi (y_2)\big |dy, \end{aligned}$$

where $\int \theta ^\epsilon (y_1-y_3)dy_3=1$ is used. The last expression is bounded by $\lesssim \epsilon ^2+(3|z|+2\epsilon )^2+|z|$ as already analyzed in (85). This yields (88). $\square$

We continue with (65). By the elementary inequality $(a+b+c)^2\le 3(a^2+b^2+c^2)$, it can be seen that it suffices to control two type of terms, which are treated in the next two propositions. Recall that $v^{\epsilon ,z}$ is a shorthand (47).

### Proposition 23

89 $$\begin{aligned}&\lim _{|z|\rightarrow 0}\limsup _{\epsilon \rightarrow 0}\frac{1}{N^4}\int _0^T\sum _{k\in \mathbb {N}}\mathbb {E}\Bigg |\sum _{i\in \mathcal {N}\big (\zeta _t^N\big )}\sum _{j\in \mathcal {N}\big (\zeta _t^N\big ): j\ne i}v^{\epsilon ,z}\big (x_i^N(t)-x_j^N(t)\big )\nabla \phi \big (x_i^N(t)\big )\cdot \nonumber \\&\quad \lambda _ke_k^\epsilon \big (x_i^N(t)\big )\psi \big (x_j^N(t)\big )\Bigg |^2dt=0. \end{aligned}$$

### Proof

The pre-limit on the left-hand side of (89) can be expanded as

$$\begin{aligned}&\frac{1}{N^4}\int _0^T\sum _{k\in \mathbb {N}}\mathbb {E}\Bigg |\sum _{i\in \mathcal {N}\big (\zeta _t^N\big )}\sum _{j\in \mathcal {N}\big (\zeta _t^N\big ): j\ne i}v^{\epsilon ,z}\big (x_i^N(t)-x_j^N(t)\big )\nabla \phi \big (x_i^N(t)\big )\cdot \lambda _ke_k^\epsilon \big (x_i^N(t)\big )\psi \big (x_j^N(t)\big )\Bigg |^2dt\\&=\frac{1}{N^4}\int _0^T\sum _{k\in \mathbb {N}}\mathbb {E}\sum _{i\ne m} \sum _{j\ne n}v^{\epsilon ,z}\big (x_i^N(t)-x_m^N(t)\big )\psi \big (x_m^N(t)\big )\nabla \phi \big (x_i^N(t)\big )^T \lambda _ke_k^\epsilon \big (x_i^N(t)\big )\otimes \lambda _ke_k^\epsilon \big (x_j^N(t)\big )\\&\quad \cdot \nabla \phi \big (x_j^N(t)\big )\psi \big (x_n^N(t)\big ) v^{\epsilon ,z}\big (x_j^N(t)-x_n^N(t)\big )dt\\&=\frac{1}{N^4}\int _0^T\mathbb {E}\sum _{i\ne m} \sum _{j\ne n}v^{\epsilon ,z}\big (x_i^N(t)-x_m^N(t)\big )\psi \big (x_m^N(t)\big )\nabla \phi \big (x_i^N(t)\big )^TC_\epsilon \big (x_i^N(t)-x_j^N(t)\big )\\&\quad \cdot \nabla \phi \big (x_j^N(t)\big ) \psi \big (x_n^N(t)\big )v^{\epsilon ,z}\big (x_j^N(t)-x_n^N(t)\big )dt\\&=\frac{1}{N^4}\int _0^T\mathbb {E}\sum _{i=j\ne m,n}v^{\epsilon ,z}\big (x_i^N(t)-x_m^N(t)\big )\psi \big (x_m^N(t)\big ) \nabla \phi \big (x_i^N(t)\big )^T\nabla \phi \big (x_i^N(t)\big )\\&\qquad \psi \big (x_n^N(t)\big )v^{\epsilon ,z}\big (x_i^N(t)-x_n^N(t)\big )dt\\&\quad +\frac{1}{N^4}\int _0^T\mathbb {E}\sum _{i\ne j, i\ne m, j\ne n}v^{\epsilon ,z}\big (x_i^N(t)\\&\quad -x_m^N(t)\big )\psi \big (x_m^N(t)\big )\nabla \phi \big (x_i^N(t)\big )^TC_\epsilon \big (x_i^N(t)-x_j^N(t)\big )\\&\quad \cdot \nabla \phi \big (x_j^N(t)\big ) \psi \big (x_n^N(t)\big )v^{\epsilon ,z}\big (x_j^N(t)-x_n^N(t)\big )dt\\&\le \frac{1}{N^4}\int _0^T\mathbb {E}\sum _{i=j\ne m,n}\big |v^{\epsilon ,z}\big (x_i^N(t)-x_m^N(t)\big )\big |\big | v^{\epsilon ,z}\big (x_i^N(t)-x_n^N(t)\big )\big |\phi \big (x_i^N(t)\big )^2\big |\psi \big (x_m^N(t)\big )\psi \big (x_n^N(t)\big )\big |dt\\&\quad +\frac{9}{N^4}\int _0^T\mathbb {E}\sum _{i\ne j, i\ne m, j\ne n}\big |v^{\epsilon ,z}\big (x_i^N(t)-x_m^N(t)\big )\big |\big \Vert C_\epsilon \big (x_i^N(t)-x_j^N(t)\big )\big \Vert _\infty \\&\quad \cdot \big |v^{\epsilon ,z}\big (x_j^N(t)-x_n^N(t)\big )\big |\big |\phi \big (x_i^N(t)\big )\phi \big (x_j^N(t)\big )\psi \big (x_m^N(t)\big )\psi \big (x_n^N(t)\big )\big |dt, \end{aligned}$$

where recall (34) notation for maximum of matrix entries. By the bound (75) and the coupling with the auxiliary free system, we further bound the above by

$$\begin{aligned}&\lesssim \frac{\epsilon ^{-1}}{N^4}\int _0^T\mathbb {E}\sum _{i\ne m,n}\big |v^{\epsilon ,z}\big (x_i^N(t)-x_m^N(t)\big )\big |\phi \big (x_i^N(t)\big )^2\big |\psi \big (x_m^N(t)\big )\psi \big (x_n^N(t)\big )\big |dt\\&\quad +\frac{\epsilon ^{-1}}{N^4}\int _0^T\mathbb {E}\sum _{i\ne j, i\ne m, j\ne n}\big |v^{\epsilon ,z}\big (x_i^N(t)\\&\quad -x_m^N(t)\big )\big |\big \Vert C_\epsilon \big (x_i^N(t)-x_j^N(t)\big )\big \Vert _\infty \big |\phi \big (x_i^N(t)\big )\phi \big (x_j^N(t)\big )\psi \big (x_m^N(t)\big )\psi \big (x_n^N(t)\big )\big | dt\\&\le \frac{1}{N^3}\int _0^T\mathbb {E}\sum _{i\ne m,n}\big |v^{\epsilon ,z}\big (y_i^\epsilon (t)-y_m^\epsilon (t)\big )\big |\phi \big (y_i^\epsilon (t)\big )^2\big |\psi \big (y_m^\epsilon (t)\big )\psi \big (y_n^\epsilon (t)\big )\big |dt\\&\quad +\frac{1}{N^3}\int _0^T\mathbb {E}\sum _{i\ne j, i\ne m, j\ne n}\big |v^{\epsilon ,z}\big (y_i^\epsilon (t)-y_m^\epsilon (t)\big )\big |\big \Vert C_\epsilon \big (y_i^\epsilon (t)-y_j^\epsilon (t)\big )\\&\quad \big \Vert _\infty \big |\phi (y_i^\epsilon (t)\big )\phi \big (y_j^\epsilon (t)\big )\psi \big (y_m^\epsilon (t)\big )\psi \big (y_n^\epsilon (t)\big )\big | dt. \end{aligned}$$

By exchangeability of the free system, we can further write the above as

$$\begin{aligned}&\le \int _0^T\mathbb {E}\big |v^{\epsilon ,z}\big (y_1^\epsilon (t)-y_2^\epsilon (t)\big )\big |\phi \big (y_1^\epsilon (t)\big )^2\big |\psi \big (y_2^\epsilon (t)\big )\psi \big (y_3^\epsilon (t)\big )\big |dt\\&\quad +N\int _0^T\mathbb {E}\big |v^{\epsilon ,z}\big (y_1^\epsilon (t)-y_3^\epsilon (t)\big )\big |\big \Vert C_\epsilon \big (y_1^\epsilon (t)-y_2^\epsilon (t)\big )\big \Vert _\infty \\&\quad \big |\phi \big (y_1^\epsilon (t)\big )\phi \big (y_2^\epsilon (t)\big )\psi \big (y_3^\epsilon (t)\big )\psi \big (y_4^\epsilon (t)\big )\big | dt. \end{aligned}$$

Recall that a $\ell$-tuple $\big (y_1^\epsilon (t),..., y_\ell ^\epsilon (t)\big )$ of free particles, $\ell =3,4$, have a uniformly bounded joint density, see (33). Hence we may rewrite the above as

$$\begin{aligned}&=\int _0^T\iint _{(\mathbb {R}^3)^3}|v^{\epsilon ,z}(y_1-y_2)|f_t^{123,\epsilon }(y_1,y_2,y_3)\phi (y_1)^2|\psi (y_2)\psi (y_3)|dy_1dy_2dt\\&\quad \quad +N\int _0^T\iiint _{(\mathbb {R}^3)^4}|v^{\epsilon ,z}(y_1-y_3)|\Vert C_\epsilon (y_1-y_2)\\&\quad \Vert _\infty f_t^{1234,\epsilon }(y_1,y_2,y_3, y_4)\phi (y_1)\phi (y_2)\psi (y_3)\psi (y_4)dy_1dy_2dy_3dy_4dt\\&\le C_0T\int _{\mathbb {K}}|v^{\epsilon ,z}(y)|dy+C_0T N\iint _{\mathbb {K}^2}|v^{\epsilon ,z}(y_3)| \Vert C(y_2/\epsilon )\Vert _\infty dy_2dy_3\\&\le C_0T\int _{\mathbb {K}}|v^{\epsilon ,z}(y)|dy+C_0T N\epsilon ^3\int _{\mathbb {K}}|v^{\epsilon ,z}(y)|dy\int _{\mathbb {R}^3} \Vert C(z)\Vert _\infty dz, \end{aligned}$$

where $\mathbb {K}\subset \mathbb {R}^3$ is a compact set determined by the supports of $\phi ,\psi$. Note that $N\epsilon ^3=\epsilon ^2$ and we already analyzed in (85) that $\int _{\mathbb {K}}|v^{\epsilon ,z}(y)|dy\lesssim \epsilon ^2+(3|z|+2\epsilon )^2+|z|$. This yields (89). $\square$

### Proposition 24

90 $$\begin{aligned}&\lim _{|z|\rightarrow 0}\limsup _{\epsilon \rightarrow 0}\frac{1}{N^4}\int _0^T\sum _{k\in \mathbb {N}}\mathbb {E}\Bigg |\sum _{i\in \mathcal {N}\big (\zeta _t^N\big )}\sum _{j\in \mathcal {N}\big (\zeta _t^N\big ): j\ne i}\phi \big (x_i^N(t)\big )\nabla v^{\epsilon ,z}\big (x_i^N(t)-x_j^N(t)\big )\cdot \nonumber \\&\quad \lambda _ke_k^\epsilon \big (x_i^N(t)\big )\psi \big (x_j^N(t)\big )\Bigg |^2dt=0. \end{aligned}$$

### Proof

As the in the last proposition, the pre-limit on the left-hand side of (90) can be expanded as

$$\begin{aligned}&\frac{1}{N^4}\int _0^T\sum _{k\in \mathbb {N}}\mathbb {E}\Bigg |\sum _{i\in \mathcal {N}\big (\zeta _t^N\big )}\sum _{j\in \mathcal {N}\big (\zeta _t^N\big ): j\ne i}\phi \big (x_i^N(t)\big )\nabla v^{\epsilon ,z}\big (x_i^N(t)-x_j^N(t)\big )\cdot \lambda _ke_k^\epsilon \big (x_i^N(t)\big )\psi \big (x_j^N(t)\big )\Bigg |^2dt\\&=\frac{1}{N^4}\int _0^T\mathbb {E}\sum _{i\ne m} \sum _{j\ne n}\phi \big (x_i^N(t)\big )\psi \big (x_m^N(t)\big )\nabla v^{\epsilon ,z}\big (x_i^N(t)-x_m^N(t)\big )^TC_\epsilon \big (x_i^N(t)-x_j^N(t)\big )\\&\quad \cdot \nabla v^{\epsilon ,z}\big (x_j^N(t)-x_n^N(t)\big )\phi \big (x_j^N(t)\big ) \psi \big (x_n^N(t)\big )dt\\&\le \frac{1}{N^4}\int _0^T\mathbb {E}\sum _{i=j\ne m,n}\big |\nabla v^{\epsilon ,z}\big (x_i^N(t)-x_m^N(t)\big )\big |\big |\nabla v^{\epsilon ,z}\big (x_i^N(t)\\&\quad -x_n^N(t)\big )\big |\phi \big (x_i^N(t)\big )^2\big |\psi \big (x_m^N(t)\big )\psi \big (x_n^N(t)\big )\big |dt\\&+\frac{1}{N^4}\int _0^T\mathbb {E}\sum _{i\ne j, i\ne m, j\ne n}\big |\nabla v^{\epsilon ,z}\big (x_i^N(t)-x_m^N(t)\big )\big |\big \Vert C_\epsilon \big (x_i^N(t)-x_j^N(t)\big )\big \Vert _\infty \\&\quad \cdot \big |\nabla v^{\epsilon ,z}\big (x_j^N(t)-x_n^N(t)\big )\big |\big |\phi \big (x_i^N(t)\big )\phi \big (x_j^N(t)\big )\psi \big (x_m^N(t)\big )\psi \big (x_n^N(t)\big )\big |dt, \end{aligned}$$

We bound the two terms separately. First we treat the first term and we divide it further according to $m=n$ or $m\ne n$. In case $i=j$, $m=n$ we have

$$\begin{aligned}&\frac{1}{N^4}\int _0^T\mathbb {E}\sum _{i=j\ne m=n}\big |\nabla v^{\epsilon ,z}\big (x_i^N(t)-x_m^N(t)\big )\big |\big |\nabla v^{\epsilon ,z}\big (x_i^N(t)-x_m^N(t)\big )\big |\phi \big (x_i^N(t)\big )^2\psi \big (x_m^N(t)\big )^2dt\\&\le \frac{1}{N^4}\int _0^T\mathbb {E}\sum _{i=j\ne m=n}\big |\nabla v^{\epsilon ,z}\big (y_i^\epsilon (t)-y_m^\epsilon (t)\big )\big |\big |\nabla v^{\epsilon ,z}\big (y_i^\epsilon (t)-y_m^\epsilon (t)\big )\big |\phi \big (y_i^\epsilon (t)\big )^2\psi \big (y_m^\epsilon (t)\big )^2dt\\&\le \frac{1}{N^2}\int _0^T\mathbb {E}\big |\nabla v^{\epsilon ,z}\big (y_1^\epsilon (t)-y_2^\epsilon (t)\big )\big |\big |\nabla v^{\epsilon ,z}\big (y_1^\epsilon (t)-y_2^\epsilon (t)\big )\big |\phi \big (y_1^\epsilon (t)\big )^2\psi (y_2^\epsilon (t))^2dt\\&\lesssim \frac{\epsilon ^{-1}}{N^2}\int _0^T\mathbb {E}\big |\nabla v^{\epsilon ,z}\big (y_1^\epsilon (t)-y_2^\epsilon (t)\big )\big |\phi \big (y_1^\epsilon (t)\big )^2\psi \big (y_2^\epsilon (t)\big )^2dt, \end{aligned}$$

and we have analysed in (87) that such a term (even without the 1/*N* factor) is negligible. In case $i=j$, $m\ne n$, we have

$$\begin{aligned}&\frac{1}{N^4}\int _0^T\mathbb {E}\sum _{i=j\ne m\ne n}\big |\nabla v^{\epsilon ,z}\big (x_i^N(t)-x_m^N(t)\big )\big |\big |\nabla v^{\epsilon ,z}\big (x_i^N(t)\\&\quad -x_n^N(t)\big )\big |\phi \big (x_i^N(t)\big )^2\big |\psi \big (x_m^N(t)\big )\psi \big (x_n^N(t)\big )\big |dt\\&\le \frac{1}{N^4}\int _0^T\mathbb {E}\sum _{i=j\ne m\ne n}\big |\nabla v^{\epsilon ,z}\big (y_i^\epsilon (t)-y_m^\epsilon (t)\big )\big |\big |\nabla v^{\epsilon ,z}\big (y_i^\epsilon (t)\\&\quad -y_n^\epsilon (t)\big )\big |\phi \big (y_i^\epsilon (t)\big )^2\big |\psi \big (y_m^\epsilon (t)\big )\psi \big (y_n^\epsilon (t)\big )\big |dt\\&\le \frac{1}{N}\int _0^T\mathbb {E}\big |\nabla v^{\epsilon ,z}\big (y_1^\epsilon (t)-y_2^\epsilon (t)\big )\big |\big |\nabla v^{\epsilon ,z}\big (y_1^\epsilon (t)-y_3^\epsilon (t)\big )\big |\phi \big (y_1^\epsilon (t)\big )^2\big |\psi \big (y_2^\epsilon (t)\big )\psi \big (y_3^\epsilon (t)\big )\big |dt. \end{aligned}$$

Recall that the joint density of a triple of free particles $\left( y_1^\epsilon (t), y_2^\epsilon (t), y_3^\epsilon (t)\right)$ has the bound (33), hence we have (even without the 1/*N* factor)

$$\begin{aligned}&\int _0^T\mathbb {E}\big |\nabla v^{\epsilon ,z}\big (y_1^\epsilon (t)-y_2^\epsilon (t)\big )\big |\big |\nabla v^{\epsilon ,z}\big (y_1^\epsilon (t)-y_3^\epsilon (t)\big )\big |\phi \big (y_1^\epsilon (t)\big )^2\big |\psi \big (y_2^\epsilon (t)\big )\psi \big (y_3^\epsilon (t)\big )\big |dt\\&=\int _0^T\iiint _{(\mathbb {R}^3)^3}\big |\nabla v^{\epsilon ,z}(y_1-y_2)\big |\big |\nabla v^{\epsilon ,z}(y_1\\&\quad -y_3)\big |\phi (y_1)^2\big |\psi (y_2)\psi (y_3)\big |f_t^{123,\epsilon }(y_1,y_2,y_3)dy_1dy_2dy_3\\&\le TC_0\iiint _{(\mathbb {R}^3)^3}\big |\nabla v^{\epsilon ,z}(y_1-y_2)\big |\big |\nabla v^{\epsilon ,z}(y_1-y_3)\phi (y_1)^2\big |\psi (y_2)\psi (y_3)\big |dy_1dy_2dy_3\\&\lesssim \left( \int _{\mathbb {K}}\big |\nabla v^{\epsilon ,z}(y)\big |dy\right) ^2, \end{aligned}$$

where $\mathbb {K}$ is a compact set determined by the supports of $\phi ,\psi$. Since from (87) we know that $\int _{\mathbb {K}}|\nabla v^{\epsilon ,z}(y)|dy$ is negligible, so is the whole term.

To treat the second main term involving the covariance $C_\epsilon$, we proceed as in the proof of Proposition 23, using the bound (76) and coupling with the auxiliary system

$$\begin{aligned}&\frac{1}{N^4}\int _0^T\mathbb {E}\sum _{i\ne j, i\ne m, j\ne n}\big |\nabla v^{\epsilon ,z}\big (x_i^N(t)-x_m^N(t)\big )\big |\big \Vert C_\epsilon \big (x_i^N(t)-x_j^N(t)\big )\big \Vert _\infty \\&\quad \cdot \big |\nabla v^{\epsilon ,z}\big (x_j^N(t)-x_n^N(t)\big )\big |\big |\phi \big (x_i^N(t)\big )\phi \big (x_j^N(t)\big )\psi \big (x_m^N(t)\big )\psi \big (x_n^N(t)\big )\big |dt\\&\lesssim \frac{\epsilon ^{-2}}{N^4}\int _0^T\mathbb {E}\sum _{i\ne j, i\ne m, j\ne n}\big |\nabla v^{\epsilon ,z}\big (x_i^N(t)-x_m^N(t)\big )\big | \big \Vert C_\epsilon \big (x_i^N(t)\\&\quad -x_j^N(t)\big )\big \Vert _\infty |\phi \big (x_i^N(t)\big )\phi \big (x_j^N(t)\big )\psi \big (x_m^N(t)\big )\psi \big (x_n^N(t)\big )\big | dt\\&\le \epsilon ^{-2}\int _0^T\mathbb {E}\big |\nabla v^{\epsilon ,z}\big (y_1^\epsilon (t)-y_2^\epsilon (t)\big )\big |\big \Vert C_\epsilon \big (y_1^\epsilon (t)-y_3^\epsilon (t)\big )\big \Vert _\infty |\\&\quad \phi \big (x_1^N(t)\big )\phi \big (x_2^N(t)\big )\psi \big (x_3^N(t)\big )\psi \big (x_4^N(t)\big )\big | dt\\&\lesssim \epsilon ^{-2}\epsilon ^3\int _{\mathbb {K}}|\nabla v^{\epsilon ,z}(y)|dy\int _{\mathbb {R}^3} \Vert C(z)\Vert _\infty dz. \end{aligned}$$

Notice that we have a factor of $\epsilon$ and in addition $\int _{\mathbb {K}}|\nabla v^{\epsilon ,z}(y)|dy$ is negligible. This completes the proof of (90). $\square$

We continue with (66). By the elementary inequality $(a+b)^2\le 2(a^2+b^2)$, it suffices to control separately two terms in the following proposition.

### Proposition 25

91 $$\begin{aligned}&\lim _{|z|\rightarrow 0}\limsup _{\epsilon \rightarrow 0}\frac{1}{N^4}\sum _{i\in \mathcal {N}\big (\zeta _t^N\big )}\int _0^T \mathbb {E}\Bigg |\sum _{j\in \mathcal {N}\big (\zeta _t^N\big ): j\ne i}\nabla v^{\epsilon ,z}\big (x_i^N(t)-x_j^N(t)\big )\phi \big (x_i^N(t)\big )\psi \big (x_j^N(t)\big )\Bigg |^2 dt\nonumber \\  &\quad =0.\nonumber \\&\lim _{|z|\rightarrow 0}\limsup _{\epsilon \rightarrow 0}\frac{1}{N^4}\sum _{i\in \mathcal {N}\big (\zeta _t^N\big )}\int _0^T \mathbb {E}\Bigg |\sum _{j\in \mathcal {N}\big (\zeta _t^N\big ): j\ne i}v^{\epsilon ,z}\big (x_i^N(t)-x_j^N(t)\big )\nabla \phi \big (x_i^N(t)\big )\psi \big (x_j^N(t)\big )\Bigg |^2 dt\nonumber \\  &\quad =0. \end{aligned}$$

### Proof

The proof of the two statements are similar and for brevity we only treat the first one (91). The pre-limit of the left-hand side of (91) can be bounded by

$$\begin{aligned}&\frac{1}{N^4}\sum _{i\in \mathcal {N}\big (\zeta _t^N\big )}\int _0^T \mathbb {E}\Bigg |\sum _{j\in \mathcal {N}\big (\zeta _t^N\big ): j\ne i}\nabla v^{\epsilon ,z}\big (x_i^N(t)-x_j^N(t)\big )\phi \big (x_i^N(t)\big )\psi \big (x_j^N(t)\big )\Bigg |^2 dt\\&=\frac{1}{N^4}\sum _{i\in \mathcal {N}\big (\zeta _t^N\big )}\int _0^T \mathbb {E}\sum _{\begin{array}{c} j, m\in \mathcal {N}\big (\zeta _t^N\big ): \\ j, m\ne i \end{array}}\nabla v^{\epsilon ,z}\big (x_i^N(t)-x_j^N(t)\big )\cdot \nabla v^{\epsilon ,z}\big (x_i^N(t)\\&\quad -x_m^N(t)\big )\phi \big (x_i^N(t)\big )^2\psi \big (x_j^N(t)\big )\psi \big (x_m^N(t)\big ) dt\\&\le \frac{1}{N^4}\sum _{i\in \mathcal {N}\big (\zeta _t^N\big )}\int _0^T \mathbb {E}\sum _{j, m\in \mathcal {N}\big (\zeta _t^N\big ): j=m\ne i} \big |\nabla v^{\epsilon ,z}\big (x_i^N(t)-x_j^N(t)\big )\big |\big |\nabla v^{\epsilon ,z}\big (x_i^N(t)\\&\quad -x_j^N(t)\big )\big |\phi \big (x_i^N(t)\big )^2\psi \big (x_j^N(t)\big )^2dt\\&\quad +\frac{1}{N^4}\sum _{i\in \mathcal {N}\big (\zeta _t^N\big )}\int _0^T \mathbb {E}\sum _{\begin{array}{c} j, m\in \mathcal {N}\big (\zeta _t^N\big ):\\ j\ne m, \, j,m\ne i \end{array}}\big |\nabla v^{\epsilon ,z}\big (x_i^N(t)-x_j^N(t)\big )\big |\big |\nabla v^{\epsilon ,z}\big (x_i^N(t)\\&\quad -x_m^N(t)\big )\big |\phi \big (x_i^N(t)\big )^2\psi \big (x_j^N(t)\big )\psi \big (x_m^N(t)\big ) dt. \end{aligned}$$

However, notice that this type of terms are already treated in the proof of Proposition 24. $\square$

We continue with (67). There are two terms and we bound them separately in the next two propositions.

### Proposition 26

92 $$\begin{aligned}&\lim _{|z|\rightarrow 0}\limsup _{\epsilon \rightarrow 0}\mathbb {E}\Bigg |\frac{1}{N^5}\int _0^T\sum _{i\in \mathcal {N}\big (\zeta _t^N\big )}\sum _{j\in \mathcal {N}\big (\zeta _t^N\big ): j\ne i}\theta ^\epsilon \big (x_i^N(t) -x_j^N(t)\big )\big |v^{\epsilon ,z}\big (x_i^N(t)\nonumber \\&\quad -x_j^N(t)\big )\phi \big (x_i^N(t)\big )\psi \big (x_j^N(t)\big )\big |^2dt\Bigg |=0. \end{aligned}$$

### Proof

By (75) and coupling with the auxiliary free system, the pre-limit on the left-hand side of (92) can bounded by

$$\begin{aligned}&\le (\epsilon ^{-1})^2\frac{1}{N^5}\int _0^T\mathbb {E}\sum _{i\in \mathcal {N}\big (\zeta _t^N\big )}\sum _{j\in \mathcal {N}\big (\zeta _t^N\big ): j\ne i}\theta ^\epsilon \big (x_i^N(t) -x_j^N(t)\big )\phi \big (x_i^N(t)\big )^2\psi \big (x_j^N(t)\big )^2dt\\&\le \frac{1}{N^3}\int _0^T\mathbb {E}\sum _{i\ne j=1}^N\theta ^\epsilon \big (y_i^\epsilon (t) -y_j^\epsilon (t)\big )\phi \big (y_i^\epsilon (t)\big )^2\psi \big (y_j^\epsilon (t)\big )^2dt\\&\le \frac{1}{N}\int _0^T\mathbb {E}\; \theta ^\epsilon \big (y_1^\epsilon (t) -y_2^\epsilon (t)\big )\phi \big (y_1^\epsilon (t)\big )^2\psi \big (y_2^\epsilon (t)\big )^2dt \\&= \frac{1}{N}\int _0^T\iint _{(\mathbb {R}^3)^2} \theta ^\epsilon (y_1-y_2)\phi (y_1)^2\psi (y_2)^2f_t^{12,\epsilon }(y_1,y_2)dy_1dy_2dt=O(N^{-1}), \end{aligned}$$

where $\int \theta ^\epsilon =1$ is used. This yields (92). $\square$

### Proposition 27

93 $$\begin{aligned}&\lim _{|z|\rightarrow 0}\limsup _{\epsilon \rightarrow 0} \frac{1}{N^5}\mathbb {E}\int _0^T\sum _{i\in \mathcal {N}\big (\zeta _t^N\big )}\sum _{j\in \mathcal {N}\big (\zeta _t^N\big ): j\ne i}\theta ^\epsilon \big (x_i^N(t)-x_j^N(t)\big )\nonumber \\&\quad \cdot \Bigg |\sum _{k\in \mathcal {N}\big (\zeta _t^N\big ): k\ne i,j}v^{\epsilon ,z}\big (x_i^N(t)-x_k^N(t)\big )\phi \big (x_i^N(t)\big )\psi \big (x_k^N(t)\big )\Bigg |^2dt=0. \end{aligned}$$

### Proof

Th prelimit on the left-hand side of (93) can be bounded by

$$\begin{aligned}&=\frac{1}{N^5}\mathbb {E}\int _0^T\sum _{i\ne j}\sum _{k, \ell \ne i,j}\theta ^\epsilon \big (x_i^N(t)-x_j^N(t)\big )v^{\epsilon ,z}\big (x_i^N(t)-x_k^N(t)\big )v^{\epsilon ,z}\big (x_i^N(t)\\&\quad -x_\ell ^N(t)\big )\phi \big (x_i^N(t)\big )^2\psi \big (x_k^N(t)\big )\psi \big (x_\ell ^N(t)\big )dt\\&\lesssim \epsilon ^{-1}\frac{1}{N^5}\mathbb {E}\int _0^T\sum _{i\ne j; k,\ell \ne i,j}\theta ^\epsilon \big (x_i^N(t)-x_j^N(t)\big )\big |v^{\epsilon ,z}\big (x_i^N(t)\\&\quad -x_k^N(t)\big )\big |\phi \big (x_i^N(t)\big )^2\big |\psi \big (x_k^N(t)\big )\psi \big (x_\ell ^N(t)\big )\big |dt, \end{aligned}$$

where we have used (75). Using the auxiliary free system, its exchangeability, and that $(y^\epsilon _1(t),..., y^\epsilon _4 (t))$ has a uniformly bounded density, see Proposition 7, we further bound the above by

$$\begin{aligned}&\le \frac{1}{N^4}\int _0^T\sum _{i\ne j; k,\ell \ne i,j}\theta ^\epsilon \big (y_i^\epsilon (t)-y_j^\epsilon (t)\big )\big |v^{\epsilon ,z}\big (y_i^\epsilon (t)-y_k^\epsilon (t)\big )\big |\phi \big (y_i^\epsilon (t)\big )^2\big |\psi \big (y_k^\epsilon (t)\big )\psi \big (y_\ell ^\epsilon (t)\big )\big |dt\\&\le \int _0^T \theta ^\epsilon \big (y_1^\epsilon (t)-y_2^\epsilon (t)\big )\big |v^{\epsilon ,z}\big (y_1^\epsilon (t)-y_3^\epsilon (t)\big )\big |\phi \big (y_1^\epsilon (t)\big )^2\big |\psi \big (y_3^\epsilon (t)\big )\psi \big (y_4^\epsilon (t)\big )\big |dt\\&\le \int _0^T\iiint _{(\mathbb {R}^3)^4}\theta ^\epsilon (y_1-y_2)\big |v^{\epsilon ,z}(y_1-y_3)\phi (y_1)^2\big |\psi (y_3)\psi (y_4)\big |f_t^{1234,\epsilon }(y_1,y_2,y_3, y_4)dt\\&\lesssim \int _{\mathbb {K}}|v^{\epsilon ,z}(y_3)|dy_3, \end{aligned}$$

where $\mathbb {K}$ is a compact set determined by the supports of $\phi ,\psi$, and we used that $\int _{\mathbb {R}^3}\theta ^\epsilon (y_1-y_2)dy_2=1$ for any $y_1$. We already analyzed in (85) that $\int _{\mathbb {K}}|v^{\epsilon ,z}(y_3)|dy_3\lesssim \epsilon ^2+(3|z|+2\epsilon )^2+|z|$. This yields (93). $\square$

This completes the verification that all the terms (51), (53), (54), (55), (57), (58), (60), (63), (64), (65), (66) and (67) are negligible in the sense that $\lim _{|z|\rightarrow 0}\limsup _{\epsilon \rightarrow 0}\mathbb {E}|(*)|=0$, where $(*)$ stands for each of these terms.

## Potential Theory and Consequence for Particle Coalescence

Let us summarize the result of our main theorem by saying that we have introduced a non-inertial model for particle coalescence, driven by a common noise, and we have obtained in the scaling limit a pde for the limit density with a mean coalescence rate $\overline{\mathcal {R}}$ given by

$$ \overline{\mathcal {R}}=\overline{\mathcal {R}}\left( \xi , \sigma ^2,R_{0}\right) :=R_{0}\int _{\mathbb {R}^3}\theta \left( x\right) \left( 1+u\left( x\right) \right) dx $$

where $u:\mathbb {R}^3\rightarrow \mathbb {R}$ solves the auxiliary cell-equation

$$ \sigma _{0}^{2}\Delta u(x)+\sigma ^2\nabla \cdot \left( \omega (\xi x)\nabla u(x)\right) =R_{0}\theta (x)\left( 1+u(x)\right) . $$

Let us develop a few potential theory preliminaries and deduce properties of $\overline{\mathcal {R}}\left( \xi , \sigma ^2,R_{0}\right)$.

Given a uniformly elliptic, bounded and $\mathsf C^{1, \alpha }$ matrix-valued function $A:\mathbb {R}^3\rightarrow \mathbb {R}^{3\times 3}$, for some $\alpha \in (0,1)$, call $G_{A}\left( x,y\right)$ the associated Green kernel. Given a non-negative integrable $\mathsf C^\alpha$ function $\theta :\mathbb {R} ^3\rightarrow \mathbb {R}$ with compact support in *B*(0, 1), for every $\gamma >0$ consider the elliptic problem

94 $$\begin{aligned} \nabla \cdot \left( A\left( x\right) \nabla u^{\gamma }\left( x\right) \right) =\gamma \theta \left( x\right) \left( 1+u^{\gamma }\left( x\right) \right) . \end{aligned}$$

There exists a unique $\mathsf C^2(\mathbb {R}^3)$ solution $u^\gamma$ to (94), in the class of those *u* with $|u(x)|\rightarrow 0$ as $|x|\rightarrow \infty$. The proof is a modification of [13, Theorem 6.1]. Here we only repeat the proof of a weaker result, namely uniqueness within the class of those $u\in \mathsf C^2$ such that $|u(x)|=O(|x|^{-1})$ and $|\nabla u(x)|=O(|x|^{-2})$. In (74) we constructed explicitly a solution $u^\gamma$ to (94) that satisfies these decay conditions cf. Lemma 13 (but we use a different Green function there). To prove this claim, consider $u_1,u_2$ two solutions of (94) in this class and let $\widetilde{u}=u_1-u_2$. Then

$$ \nabla \cdot \left( A\left( x\right) \nabla \widetilde{u}\left( x\right) \right) =\gamma \theta \left( x\right) \widetilde{u}\left( x\right) . $$

Multiplying $\widetilde{u}$ on both sides and integrating by parts in the ball *B*(0, *R*), we get

$$ -\int _{B(0,R)}\nabla \widetilde{u}(x)^TA(x)\nabla \widetilde{u}(x)dx +\int _{\partial B(0,R)}u(x)A(x)\nabla \widetilde{u}(x) \cdot d\textbf{n}(x)= \int _{B(0,R)}\gamma \theta (x)\widetilde{u}(x)^2, $$

where $\textbf{n}(x)$ denotes the unit outward normal vector. Since *A*(*x*) is component-wise bounded, by the decay assumption,

$$ \left| \int _{\partial B(0,R)}u(x)A(x)\nabla \widetilde{u}(x) \cdot d\textbf{n}(x)\right| \lesssim R^{-1}R^{-2}|\partial B(0,R)|=O(R^{-1}). $$

By the positive-definiteness of *A*, taking $R\rightarrow \infty$ we conclude that

$$ \int _{\mathbb {R}^3}\nabla \widetilde{u}(x)^TA(x)\nabla \widetilde{u}(x)dx=\int _{\mathbb {R}^3} \gamma \theta (x)\widetilde{u}(x)^2 =0 $$

This forces $\widetilde{u}(x)=u_1(x)-u_2(x)=0$ for all $x\in \mathbb {R}^3$.

We proceed to define capacity for our divergence-form operator, a generalization of classical capacity in the case of $\Delta$. Given a compact set $K\subset \mathbb {R}^3$, consider the problems:

$$\begin{aligned}&C_{1}\left( K,A\right) \\  &\quad :=\inf \left\{ \int _{\mathbb {R}^3}\nabla \psi \left( x\right) ^{T}A\left( x\right) \nabla \psi \left( x\right) dx; \; \psi \left( x\right) {\text { is smooth, }}\psi \ge 1{\text { in a neighborhood of }}K\right\} \\&C_{2}\left( K,A\right) \\  &\quad :=\sup \left\{ \mu \left( K\right) ;\mu {\text { measure supported in }}K {\text { s.t. }}\int _{\mathbb {R}^3}G_{A}\left( x,y\right) \mu \left( dy\right) \le 1{\text { for all }}x\in \mathbb {R}^3\right\} . \end{aligned}$$

By [2, Theorem 5.5.5 (i), Lemma 5.5.2, Definition 5.4.1] and [18, Theorem 4.1 and Eq. (6.5)], $C_{1}\left( K,A\right) =C_{2}\left( K,A\right)$ and thus both can serve as equivalent definitions of capacity associated with operator *A*. Call it ${\text {Cap}}\left( K,A\right)$.

### Lemma 28

If $K_{\theta }\subset \mathbb {R}^3$ is the support of $\theta$, then

$$ \lim _{\gamma \rightarrow \infty }\gamma \int _{\mathbb {R}^3}\theta \left( x\right) \left( 1+u^{\gamma }\left( x\right) \right) dx=\textrm{Cap}\left( K_{\theta },A\right) . $$

### Proof

Using the equivalent definition $C_2(K, A)$, the proof is the same as that of [13, Theorem 6.2]. $\square$

Coming back to the problem of understanding $\overline{\mathcal {R}}\left( \xi ,\sigma ^2,R_{0}\right)$, the previous results imply:

### Corollary 29

For every $$\sigma _{0},\sigma >0$$,

$$ \lim _{R_{0}\rightarrow \infty }\overline{\mathcal {R}}\left( \xi ,\sigma ^2,R_{0}\right) =\textrm{Cap}\left( K_{\theta },\left[ \sigma _{0}^{2}I_3 +\sigma ^2\omega \left( \xi \cdot \right) \right] \right) . $$

Call “hard sphere coalescence” rate the number

$$ \overline{\mathcal {R}}_{\infty }\left( \xi ,\sigma ^2\right) :={\text {Cap}}\left( K_{\theta },\left[ \sigma _{0}^{2}I_3+\sigma ^2\omega \left( \xi \cdot \right) \right] \right) , $$

over which we put our main attention.

### Remark 30

We call it hard sphere coalescence, since as $R_0\rightarrow \infty$, the microscopic coalescence rate (20) tends to infinity, thus two particles that do interact (i.e. are $\epsilon$-close) surely annihilate. That is, in this limit, our stochastic interaction rule becomes deterministic. This reminds us of deterministic collision as in Boltzmann’s theory of hard spheres. While we do not claim there is any essential connection to that area, see [24] for a study of particle approximation to Boltzmann equation with stochastic collision rate, where a rate similar to (20) appears.

Call ${\text {Cap}}\left( K_{\theta }\right)$ the classical capacity of $K_{\theta }$ (i.e. the one associated with $\Delta$). Our main result for the theory of turbulent coalescence is:

### Theorem 31

$$ \lim _{\xi \rightarrow 0}\overline{\mathcal {R}}_{\infty }\left( \xi , \sigma ^2\right) =\sigma _{0}^{2}\textrm{Cap}\left( K_{\theta }\right) $$

$$ \lim _{\xi \rightarrow \infty }\overline{\mathcal {R}}_{\infty }\left( \xi , \sigma ^2 \right) =\left( \sigma _{0}^{2}+\sigma ^2\right) \textrm{Cap}\left( K_{\theta }\right) . $$

### Proof

Using the equivalent definition $C_{1}(K,A)$, the claims follow from the monotonicity of quadratic form in terms of the matrix ordering, since $\lim _{\xi \rightarrow \infty }\omega (\xi x)=I_3$ and $\lim _{\xi \rightarrow 0}\omega (\xi x)=0$ for every *x*. $\square$

The first claim says that the coalescence rate becomes very small as the scale ratio $\xi$ tends to zero, since $\sigma _{0}^{2}$ is assumed very small with respect to the other constants. The second claim is expressed as a limit as $\xi \rightarrow \infty$, in order to have a sharp mathematical result but from the viewpoint of physical interpretation it must be understood as a property just for non-small $\xi$. It clearly states that $\overline{\mathcal {R}}_{\infty }\left( \xi , \sigma ^2\right)$ increases with $\sigma ^2$, which was the other one of our desired properties.

## Data Availability

Not applicable (no associated data).
